# DNA sequence analysis landscape: a comprehensive review of DNA sequence analysis task types, databases, datasets, word embedding methods, and language models

**DOI:** 10.3389/fmed.2025.1503229

**Published:** 2025-04-08

**Authors:** Muhammad Nabeel Asim, Muhammad Ali Ibrahim, Arooj Zaib, Andreas Dengel

**Affiliations:** ^1^German Research Center for Artificial Intelligence GmbH, Kaiserslautern, Germany; ^2^Intelligentx GmbH (intelligentx.com), Kaiserslautern, Germany; ^3^Department of Computer Science, Technical University of Kaiserslautern, Kaiserslautern, Germany

**Keywords:** computational biology, computational genomics, DNA sequence analysis, artificial intelligence, deep learning

## Abstract

Deoxyribonucleic acid (DNA) serves as fundamental genetic blueprint that governs development, functioning, growth, and reproduction of all living organisms. DNA can be altered through germline and somatic mutations. Germline mutations underlie hereditary conditions, while somatic mutations can be induced by various factors including environmental influences, chemicals, lifestyle choices, and errors in DNA replication and repair mechanisms which can lead to cancer. DNA sequence analysis plays a pivotal role in uncovering the intricate information embedded within an organism's genetic blueprint and understanding the factors that can modify it. This analysis helps in early detection of genetic diseases and the design of targeted therapies. Traditional wet-lab experimental DNA sequence analysis through traditional wet-lab experimental methods is costly, time-consuming, and prone to errors. To accelerate large-scale DNA sequence analysis, researchers are developing AI applications that complement wet-lab experimental methods. These AI approaches can help generate hypotheses, prioritize experiments, and interpret results by identifying patterns in large genomic datasets. Effective integration of AI methods with experimental validation requires scientists to understand both fields. Considering the need of a comprehensive literature that bridges the gap between both fields, contributions of this paper are manifold: It presents diverse range of DNA sequence analysis tasks and AI methodologies. It equips AI researchers with essential biological knowledge of 44 distinct DNA sequence analysis tasks and aligns these tasks with 3 distinct AI-paradigms, namely, classification, regression, and clustering. It streamlines the integration of AI into DNA sequence analysis tasks by consolidating information of 36 diverse biological databases that can be used to develop benchmark datasets for 44 different DNA sequence analysis tasks. To ensure performance comparisons between new and existing AI predictors, it provides insights into 140 benchmark datasets related to 44 distinct DNA sequence analysis tasks. It presents word embeddings and language models applications across 44 distinct DNA sequence analysis tasks. It streamlines the development of new predictors by providing a comprehensive survey of 39 word embeddings and 67 language models based predictive pipeline performance values as well as top performing traditional sequence encoding-based predictors and their performances across 44 DNA sequence analysis tasks.

## 1 Introduction

Deoxyribonucleic acid (DNA) functions as the blueprint of life as it contains essential instructions for the development, operation, growth, and reproduction of all living organisms (1). Organisms utilize cell division process to grow from fertilized egg to a multicellular adult. Throughout an organism's lifespan, the health of tissues and organs is maintained through a continuous cycle of cell replacement. In this cycle, worn-out or damaged cells are systematically replaced with new, healthy cells. When a cell divides, each new cell requires an exact copy of the DNA to function correctly (1). DNA replication and repair processes ensure that each daughter cell receives the same genetic information as the parent cell, which is essential for the survival and proper functioning of all living organisms (2). DNA sequence changes occur through two fundamental mechanisms: germline mutations inherited from parents and somatic mutations acquired during an individual's lifetime (3). Germline mutations are present in all cells and can be passed to offspring, underlying hereditary conditions. Somatic mutations occur post-conception and can be caused by various factors including internal factors such as cellular metabolites, replication errors, and spontaneous chemical changes and external factors such as ionizing radiation, chemical mutagens, environmental pollutants, and lifestyle factors (3, 4). Understanding these distinct mutation types is crucial as they require different analytical approaches. Germline mutation analysis typically involves comparing an individual's sequence to population databases, while somatic mutation analysis often requires comparing affected tissue to unaffected tissue from the same individual. Regardless of type, mutations in genetic information can lead to complex diseases and disorders such as cancer (1). To detect susceptibility, initiation, and progression of such diseases at early stages, scientists perform large-scale DNA sequence analysis (5). Through DNA sequence analysis, scientists can decode the intricate genetic data by uncovering the origins of genetic mutations and disorders (6). In addition, this analysis is crucial for the development of targeted therapies and the advancement of personalized medicine (1).

DNA sequence analysis through traditional wet-lab experiments is expensive and time-consuming (7, 8). This is because wet-lab experiments require specialized equipment, e.g., PCR machines, and costly reagents (e.g., enzymes and chemicals). Detailed experiments on multiple patient samples may take weeks or even months. Moreover, experimentation requires careful execution and validation to prevent incorrect interpretations of genetic mutations due to errors or inconsistencies. The influx of next-generation sequencing and high-throughput approaches has given rise to huge sequences data. This abundance of genomic information has created both opportunities and challenges for comprehensive analysis. To expedite genomics sequence analysis, researchers are analyzing publicly available sequences data by harnessing the capabilities of Artificial Intelligence (AI) methods. It is important to mention that AI approaches serve to augment rather than replace experimental methods in DNA sequence analysis. For example, in precision medicine, AI models trained on large genomic databases can help to interpret patient-specific data by identifying relevant patterns and potential functional impacts. However, patient-specific experimental data remain essential, particularly for understanding unique aspects of individual cases such as tumor mutations. Thus, AI methods provide a valuable tool for generating hypotheses and guiding experimental design while working in concert with traditional molecular biology approaches.

While DNA sequence analysis encompasses a broad range of computational approaches in bioinformatics, from genome assembly and variant detection to evolutionary analysis and microbiome studies, this review focuses specifically on DNA sequence analysis tasks that involve pattern recognition and prediction, where artificial intelligence approaches can be effectively applied. These tasks include predicting functional elements, identifying regulatory regions, and classifying sequence types applications where AI can learn complex sequence patterns that may not be apparent through traditional computational methods.

**Figure 1:**
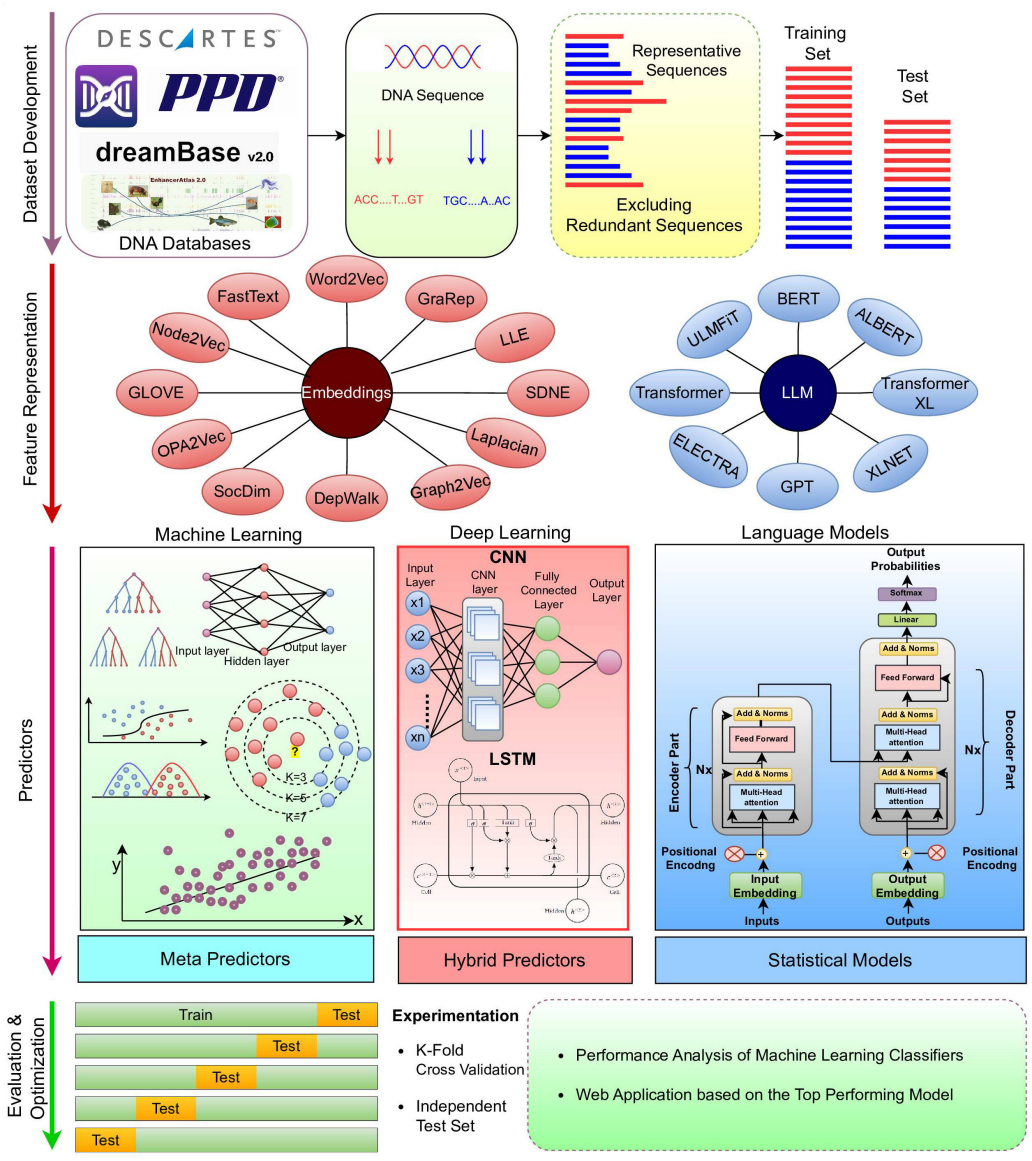
Predictive pipeline of DNA sequence analysis tasks.

Most of the AI-based genomics sequence analysis methods fall under the hood of regression and classification paradigms (9–11). Figure 1 illustrates a unified workflow of AI-based predictive pipelines for genomics sequence analysis tasks. It is evident in the Figure that, overall, AI predictive pipelines can be divided into **4** different stages (12). First stage emphasizes on the collection and development of quality benchmark datasets using public databases (13). Second stage focuses on the characterization of raw DNA sequences in terms of statistical vectors using different kinds of sequence encoders (14–16). This is primarily done to address the inherent dependency of AI predictive pipelines on statistical vectors (17–19). In entire predictive pipeline, this stage is the most crucial one because highly informative and discriminative statistical vectors help the predictors to learn comprehensive useful patterns for accurate prediction (14–16). It is widely accepted that with quality statistical vectors, even simple machine learning predictors can produce promising performance. On contrary, with less informative and discriminative statistical representations, even sophisticated deep learning predictors fail to produce decent performance (17–19).

There is a marathon of developing powerful sequence encoders for generating highly informative and discriminative statistical vectors of raw sequences. To date, hundreds of sequence encoding methods have been developed (12) that can be broadly classified into four categories: Physico-chemical properties based methods, statistical methods (12, 20), neural word embedding methods (21), and language models (22). While physico-chemical properties based methods generate statistical vectors of raw sequences using pre-computed physical and chemical values of nucleotides, statistical methods rely on occurrence frequencies of individual or group of nucleotides with DNA sequences (12). Physico-chemical properties based and statistical methods capture the intrinsic characteristics of biological sequences, such as nucleotide composition and distributional information. However, these methods lack to capture complex relationships of nucleotides such as long range interactions of nucleotides in the sequences (12, 23). In addition, these methods may not fully capture the semantic and functional similarities between sequences (12, 23). Neural word embedding methods learn distributed representations of nucleotides in the continuous vector space. These methods capture the syntactic and semantic similarities of nucleotides by mapping them to vectors in a high-dimensional space. This enables the representation of residues with similar contexts to be closer together in the vector space. Neural word embeddings methods efficiently capture semantic and contextual information of nucleotides. However, these methods lack to efficiently handle different contexts of same nucleotides (21). Language models also learn representation of individual nucleotides or groups of nucleotides (k-mers) in an unsupervised fashion by predicting masked nucleotides based on the context of surrounding nucleotides. Language models based methods capture complex nucleotide relations; however, these methods require large amount of sequence data for training and hyperparameter optimization (22).

Third stage includes predictors that make best use of statistical vectors produced by second stage to extract informative patterns for creating decision boundaries. Overall, these predictors can be classified into two categories: machine learning and deep learning (12). Machine learning predictors require less data and computational power for training. However, these predictors lack to capture comprehensive complex relationships of nucleotide (12), whereas deep learning predictors (24) are capable to learn highly complex relationships of nucleotide. However, these predictors require a huge amount of training data and computational power (12). In fourth stage, comprehensive evaluation of predictors using different experimental settings and evaluation measures is performed (24).

AI researchers have been endeavoring to complement wet-lab-based DNA sequence analysis methods by incorporating more innovative sequence encoders at second stage and predictors at third stage of predictive pipeline. However, there is still ample room for the development of more powerful predictive pipelines. Different fields such as Natural Language Processing (NLP), Energy, and Computer Vision have seen substantial progress in the development of diverse predictive pipelines. Whereas, the DNA sequence analysis field is known for its wide range of tasks, still the progress of AI applications in this area is hindered mainly due to the lack of integration between molecular biologist and AI experts. For instance, the field of NLP has made strides with multi-task learning predictors. However, the DNA sequence analysis field lags behind due to AI experts limited understanding of the diverse range of DNA analysis tasks that could support the development of multi-task learning predictors. Furthermore, the efficacy of AI applications hinges on the availability of benchmark datasets. Although developing datasets in DNA sequence analysis is relatively straightforward due to abundance of public databases which contain raw biological sequences along with associated labels, there is a tendency among researchers to overlook existing benchmark datasets, develop new benchmark datasets, and neglect comprehensive performance comparisons with existing predictors. This oversight often complicates the determination of the most effective predictors for specific tasks. For example, up to date, according to our best of knowledge, approximately 127 predictive models have been developed and published in 59 different conferences and journals for widely studied 44 different DNA sequence analysis tasks. To enhance the performance of predictive models developed for diverse DNA sequence analysis tasks, researchers need to conduct a comprehensive examination of existing literature to find most effective algorithms for different stages of new predictive pipelines. With an aim to expedite progress in the development of fair and robust AI applications for DNA sequence analysis, numerous review articles have emerged. However, these reviews typically focus on isolated tasks rather than providing a holistic overview. Considering the need and significance of a comprehensive study that bridges the gap between AI specialists and biologists, this paper makes manifold contributions:

- It bridges the gap between DNA sequence analysis and artificial intelligence fields by presenting a diverse range of DNA analysis tasks and AI methodologies.
- It empowers AI researchers by equipping them with essential biological knowledge related to 44 distinct DNA sequence analysis tasks. It categorizes 44 different DNA sequence analysis tasks into 8 different categories on the basis of sequence analysis goals. This categorization provides a structured overview to biologists and AI researchers in navigating the complex landscape of genomics studies more efficiently.
- It streamlines the integration of AI into DNA sequence analysis by consolidating information of 36 diverse biological databases being used to develop benchmark datasets for 44 different DNA sequence analysis tasks.
- It sheds light on the nature of 44 different DNA sequence analysis tasks and categorizes them into three primary categories: regression, classification, and clustering, and three secondary categories: binary classification, multi-class classification, and multi-label classification. This categorization assists computer scientists in efficient selection of most suitable algorithms for each task category, development of more effective and specialized computational frameworks, and to significantly accelerate advancements in AI-driven genomic research.
- It provides insights of 140 benchmark datasets related to 44 distinct DNA sequence analysis tasks to ensure performance comparisons between new and existing AI predictors.
- It presents word embeddings and language models applications for 44 distinct DNA sequence analysis tasks.
- It streamlines the development of new predictors by providing a comprehensive survey of current top predictors, their performances across 44 DNA sequence analysis tasks, and their public accessibility. This comprehensive overview serves as a valuable resource for researchers developing and validating predictive pipelines in computational genomics.

It is important to note that our categorization of 44 DNA sequence analysis tasks emerges from the AI and computational biology literature rather than representing a definitive biological taxonomy. We have organized these tasks into biologically relevant groupings based on their functional and analytical similarities, while recognizing that many tasks span multiple biological domains. This organization aims to bridge the gap between computational methodologies and biological applications, although we acknowledge that future refinements with deeper domain expert input would further enhance this framework.

## 2 Research methodology

**Figure 2:**
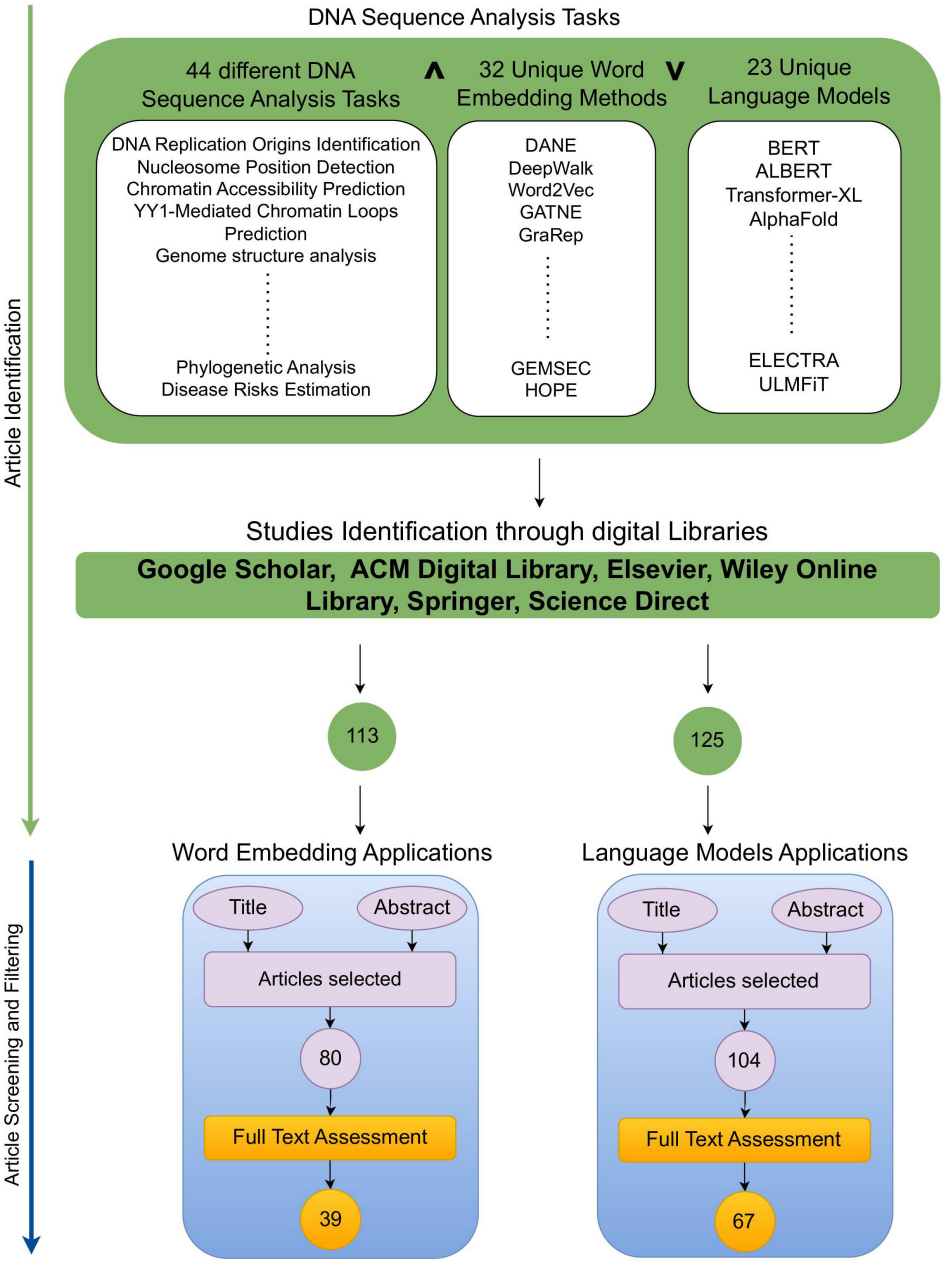
Research methodology.

This section provides a detailed overview of the research methodology used to identify articles focused on word embeddings and large language models applications in DNA sequence analysis landscape (10, 11). Figure 2 illustrates two stage processes for article identification and selection.

### 2.1 Article searching

To identify a wide range of relevant scholarly articles, initial stage involves formulation of quality search queries using different keywords. In Figure 2, article identification module contains keywords cell of three different categories, namely, DNA tasks, word embedding methods, and Language models. To formulate quality search queries, keywords within same category are combined using OR ∨ operator, while keywords of different categories are combined using AND ∧ operator. For instance, few sample search queries include DNA Replication Origins Identification using BERT language model, DNA Replication Origins Identification using DeepWalk word embedding method, etc. To acquire relevant papers, formulated search queries are executed on academic search engines such as Google Scholar,^1^ ACM Digital Library,^2^ Elsevier,^3^ IEEEXplore,^4^ Wiley Online Library,^5^ Springer,^6^ and ScienceDirect.^7^ In addition, snowballing method is employed to explore sources referenced in extracted papers to identify more research articles. This technique is particularly useful in research contexts where access to resources is limited, such as niche topics or hard-to-reach communities, as it expands the pool of resources for a study. Execution of queries across multiple academic databases acquired approximately 238 research articles which are screened and filtered in second stage.

### 2.2 Article screening and filtering

Second stage selects most relevant articles in two steps. In the first step, titles and abstracts of 113 word embeddings and 125 large language models related studies were reviewed. This review analysis identified 80 word embeddings and 104 language models related relevant articles. Second step involves full-text assessment of articles selected in first step, resulting in 39 word embeddings and 67 language models related articles.

Our selection criteria focused on DNA sequence analysis tasks where (1) raw DNA sequence data serve as the primary input, (2) AI methods extract patterns from these sequences, and (3) the analysis predicts specific biological properties or functions. This allowed us to examine AI's impact on genomic sequence interpretation while acknowledging that bioinformatics encompasses many other types of analyses not covered here.

## 3 Biological foundations of DNA sequence analysis goals and tasks

**Figure 3:**
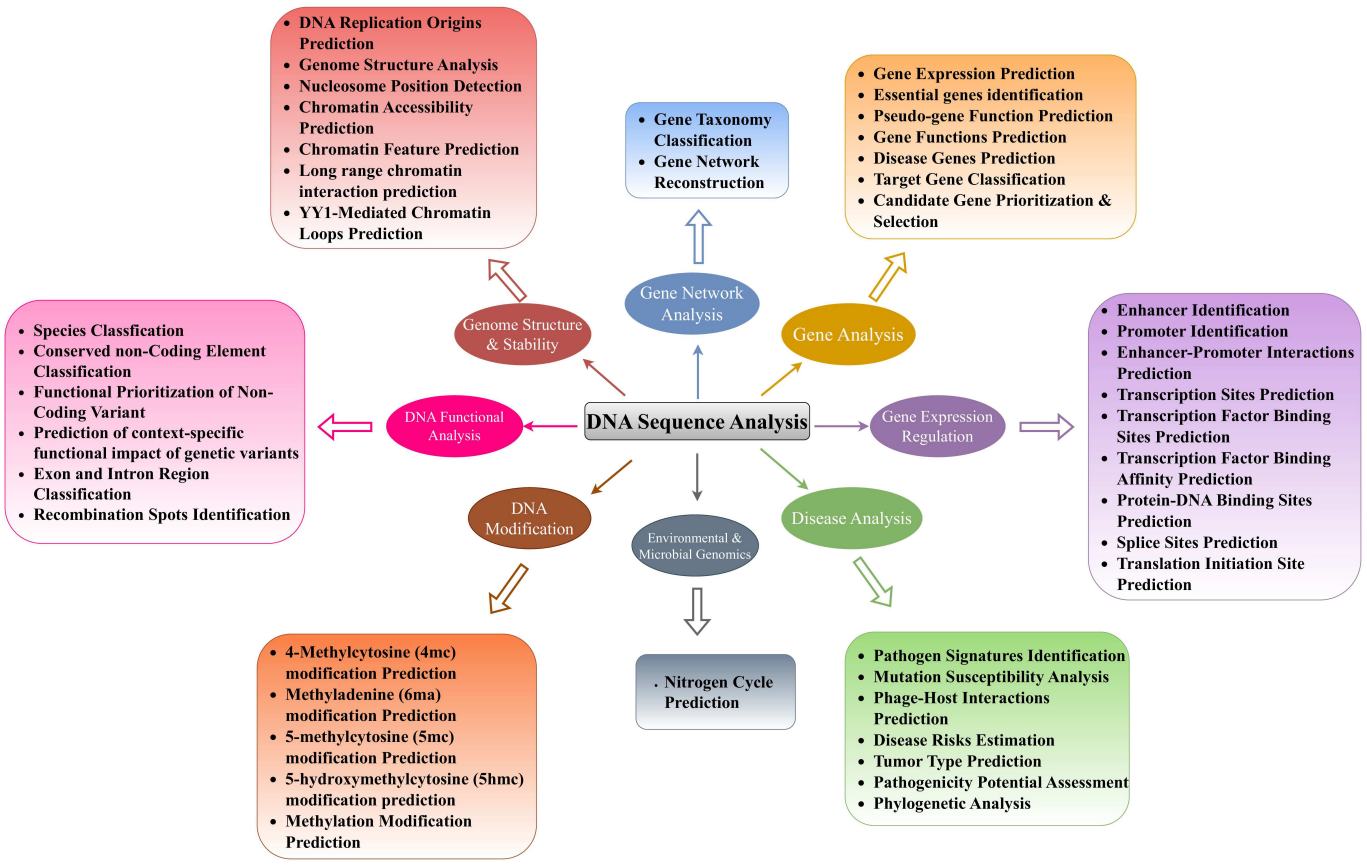
Precise classification of 44 unique DNA sequence analysis tasks in 8 major biological goals.

With an aim to find molecular basis of diseases initiation and progression, their effective detection at early stages, and development of potent drugs, researchers are trying to understand DNA sequence language by performing a variety of sequence analysis tasks. Every unique DNA sequence analysis task aims to enhance the understanding of one specific aspect of DNA, and a bunch of tasks can enhance the understanding of specific major biological goal. To summarize the biological background of 44 distinct DNA sequence analysis tasks, we have categorized them into 8 major biological goals. Figure 3 depicts the biological categorization of 44 unique DNA sequences analysis tasks into 8 different goals, namely, genome structure and stability, gene expression regulation, gene analysis, gene network analysis, DNA modification prediction, DNA functional analysis, environmental and microbial genomics, and disease analysis. This biologically informed organization was developed by analyzing both the computational biology literature and aligning with biological processes in genomics research. While computational researchers often approach these tasks through the lens of AI methodologies, we have endeavored to categorize them according to their biological relevance and function. Our categorization into 8 major biological goals represents an attempt to bridge computational approaches with biological understanding. Although we recognize the inherent complexity and interconnectedness of biological systems which indicates that many tasks could reasonably be classified in multiple categories, thus, this categorization represents one of several possible ways to organize these tasks. This categorization reflects the diverse biological applications where AI-based sequence analysis has made significant contributions. However, we recognize that DNA sequence analysis in bioinformatics extends beyond these pattern recognition tasks to include other critical applications such as genome assembly, variant detection, and population genetics studies. We specifically examine how modern AI approaches are transforming our ability to extract meaningful biological insights from sequence data through pattern-based prediction tasks.

In living organisms, DNA is packaged at multiple levels to condense vast genetic information into a well-organized structure within the cell nucleus (1). At the first level, DNA is wrapped around histone octamers also known as nucleosomes. These nucleosomes further assemble into chromatin, which then folds and condenses into an even more compact structure known as the genome (1). The exploration of genome structure and stability is pivotal in understanding the biological intricacies and potential therapeutic avenues. Genome structure can affect how genes are accessed and used. Disruptions in this structure, such as missing or misplaced DNA sections, or changes in how tightly DNA is wrapped around histone octamers, or irregularities in nucleosomes positions can lead to genes being turned on or off at the wrong times or in the wrong amounts (1). This can cause various diseases and biological disorders. DNA is an instruction manual that controls biological functioning within living organisms. If genome gets unstable, the manual gets messed up such as typos and missing sections. It can lead to uncontrolled growth of the cells (cancer) and improper working of the genes (many diseases) (1). In a nutshell, a stable genome possesses clear, complete instruction manual, essential for keeping biological functions working smooth. To better understand genome structure and stability, it is essential to explore various tasks such as DNA Replication Origins Prediction (25, 26), Genome Structure Analysis (27, 28), Nucleosome Position Detection (29, 30), Chromatin Accessibility Prediction (31–33), Chromatin Feature Prediction (31, 34, 35), Long-range Chromatin Interaction Prediction (36, 37), and YY1-Mediated Chromatin Loops Prediction (38, 39). These tasks are crucial for comprehending the intricate mechanisms governing genetic information processing and regulation within cells (40).

DNA replication origin prediction is fundamental as accurate replication of the genome is vital for maintaining genomic stability (25). The prediction of replication origins involves calculating DNA structural properties to identify sites crucial for initiating DNA replication (25). Understanding where these sites are located and how they are specified is essential for comprehending DNA replication and ensuring genome integrity (41). Genome structure analysis plays a pivotal role in deciphering the organization and arrangement of genetic material within the cell (27). By analyzing the structural features of the genome, researchers can gain insights into the functional and spatial organization of chromosomes, aiding in the identification of genomic elements involved in gene regulation and phenotypic variations (27, 42). Furthermore, nucleosome position detection is essential for understanding how nucleosomes, the basic units of genome, are arranged along the DNA strand (29, 43). This information is crucial for elucidating gene regulation mechanisms and chromatin dynamics within the cell (29, 43). Chromatin accessibility prediction is a key task that involves determining the regions of chromatin that are accessible for transcription factors and other regulatory proteins to bind (31–33). Prediction of chromatin accessibility across different cellular contexts provides valuable insights into gene regulation and chromatin dynamics (31–33). Chromatin feature prediction complements accessibility prediction by identifying specific chromatin features and epigenetic markers that influence gene expression and regulatory processes (31, 34, 35, 44). These features include transcription factor (TF) binding sites, DNase I-hypersensitive sites (DHS), and histone marks (HM). By understanding these features, researchers can unravel the mechanisms underlying chromatin regulation and gene expression (34). Long-range chromatin interactions make bridges between distant enhancers and promoters. These interactions enable interactions between enhancers and promoters by bringing them closer to each other (36, 37). YY1-mediated chromatin loop prediction provides comprehensive understanding about gene regulation (38, 39, 45). YY1 is a protein that makes loop between enhancers and promoters. These loops are essential for gene regulation, and by predicting these loops, we can see which genes can be controlled through YY1 protein (38, 39, 45). This knowledge is valuable for understanding diseases where gene regulation goes wrong. To sum up, only through multi-dimensional exploration of genome structure and stability, researchers can discriminate healthy cellular processes from malfunctioned processes, find the root causes of diseases, and develop potent therapies.

Another major goal of molecular biologists behind is gene expression regulation. Gene expression regulation provides fundamental insights into how genes are activated or repressed in response to various cellular cues (46). Specifically, researchers are trying to unravel the intricate mechanisms that control when and up to what extent specific genes are turned on or off in different cells and tissues (46). This knowledge forms the basis for understanding the functional behavior of genes in different biological contexts and sets the stage for further analyses. Hence, it holds immense promise for scientists and pharmaceutical industries. This helps scientists to detect irregularities in normal gene expression regulation, the way diseases develop at the molecular level, and identify potential drug targets (46). Furthermore, this understanding can assist pharmaceutical industries to develop improved diagnostic tools, innovative personalized therapies, and targeted interventions, which will ultimately contribute to advancements in personalized healthcare (46). In addition, it can provide a deeper understanding of biological systems which can lead to breakthroughs in biotechnology (46). For better understanding of gene expression regulation, researchers are performing nine different DNA sequence analysis tasks, namely, enhancer identification (47), promoter identification (48), enhancer-promoter interactions prediction (49), transcription site prediction (50), transcription factor binding site prediction (51), transcription factor binding affinity prediction (52), protein-DNA binding site prediction (53), splice site prediction (53), and translation initiation site prediction (54). Enhancers (47, 55–75) and promoters identification (48, 76–81), along with their interactions (82–86) prediction are important to decipher a complex control panel for gene expression (47–49). Enhancers are known as distant switches of genes, while promoters are the landing sites where gene activation starts. Identification of these elements and predicting how they loop together provide a comprehensive understanding of gene regulation, including which genes are activated or repressed, the intensity of their expression, and the specific cell types involved (87, 88). This knowledge reveals the intricate regulatory code that governs gene expression and offers valuable insights into the mechanisms underlying normal cellular function as well as the dysregulation that may contribute to various diseases.

Furthermore, prediction of different genomic sites including transcription sites (50), transcription factor binding sites (89–93), transcription factor binding site affinity (52), protein-DNA binding site (53, 94–96), splice site (93, 97–100), and translation initiation site (50, 101) provide deep insights into gene expression regulation. A transcription site refers to the specific location on the DNA where the process of transcription takes place. Transcription is the synthesis of RNA from a DNA template, and the transcription site represents the region where the RNA polymerase enzyme binds and initiates the transcription process, whereas transcription factor binding sites are specific DNA sequences where transcription factors (proteins), that regulate gene expression, bind. These binding sites are typically located near the transcription start site and are recognized by transcription factors to control the initiation or repression of transcription. In contrast, transcription factor binding site affinity refers to the strength or affinity with which a transcription factor binds to its specific binding site on DNA. It represents the likelihood of a transcription factor binding to its target site and influencing gene expression. A protein-DNA binding site refers to any region on the DNA where a protein binds. This can include transcription factors, as mentioned earlier, as well as other proteins involved in various cellular processes such as DNA replication, repair, and chromatin remodeling. Splice sites are specific sequences within a gene's DNA that mark the boundaries of introns and exons. During the process of RNA splicing, introns are removed from the pre-mRNA molecule, and exons are joined together to form the mature mRNA. Splice sites are essential for the accurate and precise splicing of RNA. Translation initiation site (TIS) is the specific location on the mRNA molecule where the process of translation begins. TIS prediction seems like a RNA sequence analysis task; however, in molecular biology research, to study gene expression, researchers are synthesizing complementary DNA (cDNA) data from messenger RNA (mRNA) template through a process called reverse transcription. In the context of cDNA data, the translation initiation site (TIS) represents the position where the ribosome, the cellular machinery responsible for protein synthesis, binds to the mRNA to initiate translation. The TIS is typically identified by the presence of specific start codons, such as AUG, which serve as signals for the ribosome to start protein synthesis.

To better understand gene functions and their roles in disease initiation, researchers are exploring various aspects such as gene expression prediction (102, 103), identification of essential (104–109) and disease-specific genes (110), gene function prediction (111, 112), pseudo-gene function prediction (111), target gene classification (113), and candidate gene prioritization (114). Overall together, these tasks provide a comprehensive platform for disease diagnosis and development of treatment strategies by uncovering disease mechanisms, identifying potential therapeutic targets, and organizing genes into functional categories. Specifically, gene expression prediction provides useful information about the level of gene activity in different cells or tissues (115). This task is vital for understanding the molecular mechanisms underlying complex diseases such as cancer and identifying potential therapeutic targets. Essential gene identification is another critical task in gene analysis that helps researchers pinpoint genes that are crucial for an organism's survival and development (116, 117). This task is particularly important in understanding gene function and the genetic basis of various disorders. Gene function prediction elucidates the roles of genes in different pathways and biological processes and provides valuable insights into disease mechanisms and potential therapeutic interventions.

Apart from gene function prediction, pseudo-gene function prediction has gained a lot of attention as a critical task in gene analysis (111). Pseudogenes were once thought to be useless DNA because they cannot code for proteins due to mutations that happened over time. However, recent studies have shown that pseudogenes actually play important roles in controlling genes, especially in cancer. For instance, the pseudogene PTENP1 helps to regulate the tumor suppressor gene PTEN in various cancer conditions, showing that pseudogenes can have important functions. Pseudogene function prediction offers numerous advantages, including better understanding of gene regulation, disease mechanisms, evolutionary biology, and the potential for new biomarkers and drug targets. In addition, disease gene prediction is a pivotal task in gene analysis that focuses on identifying genes associated with specific diseases or disorders (118). By pinpointing disease-related genes, researchers can unravel the genetic basis of diseases, discover novel biomarkers for diagnosis and prognosis, and develop targeted therapies. This task is instrumental in precision medicine approaches, where understanding the genetic underpinnings of diseases is crucial for personalized treatment strategies. Target gene classification involves categorizing genes based on their functions, interactions, or regulatory mechanisms (119). By classifying target genes, researchers can better understand gene networks, signaling pathways, and biological processes. This task is essential for deciphering the complex relationships between genes and their roles in health and disease. Candidate gene prioritization and selection are critical tasks in gene analysis that aim to identify genes with the highest likelihood of being involved in a particular biological process or disease (120). By prioritizing candidate genes, researchers can focus their efforts on studying genes that are most likely to have significant effects, accelerating the discovery of novel gene functions and disease mechanisms. This task is crucial for efficiently allocating research resources and maximizing the impact of genetic studies. Aforementioned seven DNA sequence analysis tasks are essential for advancing our understanding of genes and their roles in health and disease. By leveraging these tasks, researchers can unravel the complexities of the genome, uncover novel gene functions, and pave the way for innovative diagnostic and therapeutic strategies in various fields of biology and medicine.

Furthermore, gene network analysis is a promising goal that seeks to comprehend the intricate interactions and relationships between genes within a biological system. Two primary tasks within Gene Network Analysis are Gene Taxonomy Classification and Gene Network Reconstruction. Gene Taxonomy Classification (121–123) involves categorizing genes based on their evolutionary relationships and functional similarities, providing a structured framework for organizing genetic information. Gene Taxonomy Classification plays a crucial role in gene network analysis by offering a foundational structure for understanding the evolutionary history and functional relationships between genes. By classifying genes into taxonomic groups based on shared characteristics and evolutionary relatedness, researchers can infer valuable insights into the origins and evolutionary trajectories of genes within a network (124). This classification allows for the identification of core genes that have remained conserved throughout evolution, providing a basis for inferring phylogenetic relationships and understanding the fundamental building blocks of gene networks. Moreover, Gene Taxonomy Classification enables researchers to utilize existing knowledge about gene functions and evolutionary relationships to guide Gene Network Reconstruction. By categorizing genes into taxonomic groups, researchers can pinpoint gene clusters with similar functions or evolutionary origins, facilitating the identification of modules within gene networks that exhibit coordinated activity (125). This classification serves as a roadmap for exploring the functional roles of genes within a network and understanding how these roles have evolved over time. On the other hand, Gene Network Reconstruction (126–128) involves creating a detailed map of the interactions and regulatory relationships between genes within a cell or an organism. The primary input for gene network reconstruction is gene expression data obtained through high-throughput techniques such as RNA sequencing (RNA-seq) or microarrays. This task is pivotal for understanding how genes work together to control various biological functions and processes (129). By reconstructing gene networks, researchers can uncover key regulatory hubs involving highly connected genes, clusters of closely interacting genes, pathways, and interactions that steer cellular functions and responses to external stimuli (130).

DNA modification prediction is also a crucial goal where researchers aim is to decipher how tiny tweaks to the DNA code can lead to big changes in cellular functions (131–133). In DNA modifications, distinct chemical groups are added to specific locations on the DNA molecule. These additions do not change the actual sequence of nucleotides (A, C, G, T) but can alter the physical properties of DNA sequence. Understanding these modifications, such as 4-Methylcytosine (4mc) (134–143), Methyladenine (6ma) (144–151), 5-methylcytosine (5mc) (152, 153), 5-hydroxymethylcytosine (5hmc) (154–157), and methylation modifications (146, 154–159), are essential for advancing our comprehension of epigenetic regulation (160–162). Specifically, methylation modifications that occur due to the addition of methyl groups to DNA molecules play a pivotal role in regulating gene expression and maintaining genomic integrity. Similarly, methyladenine modifications, such as DNA N6-methyladenine (6mA), occur due to the addition of a methyl group to the adenine base of DNA. DNA 6mA modifications dynamically influence DNA thermal stability, curvature, and transcription factor interactions, impacting gene expression in a heritable manner. Understanding the prediction of 6mA sites is pivotal for both basic and clinical research as it aids in the identification of gene expression patterns and potential epigenetic changes induced by environmental factors. These predictions enhance our ability to study the role of 6mA modifications in diseases and could lead to improved therapeutic strategies, highlighting the relevance of accurate prediction methods in unraveling the complexities of DNA modifications. Moreover, 5-methylcytosine (5mc) modification occurs due to the addition of a methyl group to the cytosine base of DNA, whereas 5-hydroxymethylcytosine (5hmc) modification is an oxidized derivative of 5mc, where an additional hydroxyl group (-OH) is added to the methyl group of 5mc. Prediction of 5-methylcytosine (5mc) and 5-hydroxymethylcytosine (5hmc) modifications is essential for decoding their roles in gene regulation, developmental processes, and disease states. These critical epigenetic modifications are dynamically regulated by enzymes and influence gene expression crucial for neuronal differentiation and cellular proliferation. Abnormal levels of these modifications have been linked to diseases such as cancer. Precise prediction of 5mc and 5hmc sites is useful for the development of targeted therapies and improved prognostic assessments.

Functional genomics is also a critical goal that encompasses multiple sub-tasks including species classification (44), conserved non-coding element (NCE) classification (163), functional prioritization of non-coding variants (34), prediction of context specific functional impact of genetic variants (36), exon and intron region classification (164), and recombination spots identification (165). Each of these tasks plays a vital role in unraveling the complexities of genetic regulation and molecular mechanisms within the genome. In biomedical research, understanding the genetic similarities and differences between humans and other species is crucial for modeling diseases and studying genetic disorders. Majority of the genome is conserved across different species which makes it difficult to distinguish humans and non-human species. Despite very high genetic similarity across species (< 10% sequence divergence), small differences are extremely valuable and they have significant biological implications. Species classification determines the source species of genetic sequences based on such differences and pave way for better modeling diseases and studying genetic disorders (44). Conserved non-coding element classification is another critical task in functional genomics that focuses on identifying and understanding non-coding regions of the genome that are evolutionarily conserved across different species (163). It is essential for advancing our understanding of gene regulation, evolutionary biology, and the genetic basis of diseases. By elucidating the functions of these non-coding regions, researchers can gain insights into the intricate regulatory networks that govern gene expression and cellular processes and contribute to the development of targeted therapies.

Functional prioritization of non-coding variants (34) is another crucial task for making sense of the vast amount of genetic data generated by modern sequencing technologies. By identifying which variants have significant biological impacts, researchers can gain a deeper understanding of the genetic architecture of complex diseases, uncover novel therapeutic targets, and advance the field of precision medicine. This prioritization is essential for translating genomic research into practical health benefits and ultimately improving patient outcomes and advancing our knowledge of human biology (34). As functional prioritization of non-coding variants task involves identifying which non-coding variants among millions are likely to have functional consequences, it does not account for the specific context in which these variants might exert their effects, whereas prediction of context-specific functional impact of genetic variants aims to provide a detailed understanding of how specific variants influence gene function in different contexts (e.g., specific tissue) (36). This is particularly important for genetic studies that seek to uncover the mechanisms by which variants contribute to disease phenotypes. Unlike functional prioritization of non-coding variants task which only filters the variants that are most likely to have functional significance. Prediction of context-specific functional impact of genetic variants provides a finer level of detail by predicting the actual effect of a variant on gene expression or other functional outcomes in specific tissues. This granularity is essential for precisely understanding the specific biological mechanisms and for developing targeted therapies (36).

Exon and intron region classification is crucial for understanding gene structure and function within the genome. Exons are coding regions that are translated into proteins, while introns are non-coding regions that are spliced out during mRNA processing. By classifying exons and introns, researchers can describe gene boundaries, identify functional elements, and elucidate the mechanisms of gene expression regulation (166). This task is essential for deciphering the genetic code and unraveling the complexities of gene regulation in health and disease. Recombination spots identification is a pivotal task in functional genomics that focuses on mapping regions of the genome where genetic recombination events occur. Genetic recombination is a natural process where DNA segments are exchanged between two chromosomes during cell division. Recombination plays a vital role in generating genetic diversity, ensuring proper chromosome segregation, and driving evolution (167). By identifying recombination hot spots, researchers can gain insights into the mechanisms underlying genetic diversity and genome evolution, shedding light on the processes that shape genetic variation and adaptation in populations. In conclusion, the tasks related to functional genomics, including species classification, conserved non-coding element classification, functional prioritization of non-coding variant, prediction of context-specific functional impact of genetic variants, exon and intron region classification, and recombination spots identification, are essential for advancing our understanding of genetic regulation, molecular mechanisms, and disease pathogenesis. By delving into these tasks, researchers can unravel the complexities of the genome, decipher the genetic basis of diseases, and pave the way for precision medicine and personalized healthcare interventions tailored to an individual's genetic profile.

Another goal of researchers is to study overlap between two distinct fields namely environmental science and microbial genomics (27). This interdisciplinary study enables researchers to explore how environmental factors such as pollution, climate change, and agricultural practices affect on function and diversity of microbial communities (27). A key area of focus in this field is the nitrogen cycle prediction. By examining the genomes of microbes involved in nitrogen fixation, nitrification, and denitrification, scientists can predict how these processes might respond to environmental changes (168). This prediction provides understanding about potential impacts of environmental shifts on ecosystem health (169) and nitrogen availability, which are essential for plant growth and overall biogeochemical cycles (170).

From all eight different biological goals, disease analysis goal has received huge attention in scientific community as it aims to understand, diagnose, and treat various illnesses. Within this field, several tasks play a vital role in enhancing our comprehension of diseases. One such task is Pathogen Signatures Identification (171), which involves identifying specific markers or characteristics of pathogens that can aid in their detection and classification (172). By pinpointing these signatures, researchers can develop targeted diagnostic tools and therapies, ultimately improving disease management and control. Mutation Susceptibility Analysis (173) is another essential task in disease analysis. This task focuses on investigating the genetic variations that make individuals more prone to developing certain diseases (174). Understanding mutation susceptibility can aid in personalized medicine approaches, where individuals at higher risk can be identified early for preventive interventions or closer monitoring. Phage-Host Interactions Prediction (175–177) is a task that delves into the relationships between bacteriophages and their host bacteria (178). By predicting these interactions, researchers can gain insights into how phages influence bacterial populations, which is crucial for developing phage-based therapies to combat bacterial infections and antibiotic resistance. Disease Risks Estimation (90) is a fundamental aspect of disease analysis that involves assessing the likelihood of an individual developing a particular condition based on various factors such as genetics, lifestyle, and environmental exposures (179). Accurately estimating disease risks enables healthcare providers to offer targeted interventions and counseling to high-risk individuals, potentially preventing the onset or progression of diseases. Tumor Type Prediction (180) is a significant task in disease analysis that focuses on identifying the specific type of tumor a patient may have based on various characteristics such as genetic markers, imaging features, and histopathological findings (181). Predicting tumor types is essential for determining the most effective treatment strategies and prognostic outcomes for patients with cancer. Pathogenicity Potential Assessment (27) is a critical task that involves evaluating the ability of pathogens to cause disease in a host. By assessing the pathogenicity potential of different microorganisms, researchers can prioritize the development of interventions against the most virulent pathogens, thereby improving disease prevention and control strategies. Phylogenetic Analysis (21) is a key component of disease analysis that involves studying the evolutionary relationships between different strains of pathogens or tumor cells. Phylogenetic analysis provides insights into the origins, spread, and diversification of diseases, aiding in the development of targeted interventions and understanding disease transmission dynamics.

## 4 A look on DNA sequence analysis tasks from the perspective of computer scientists

**Figure 4:**
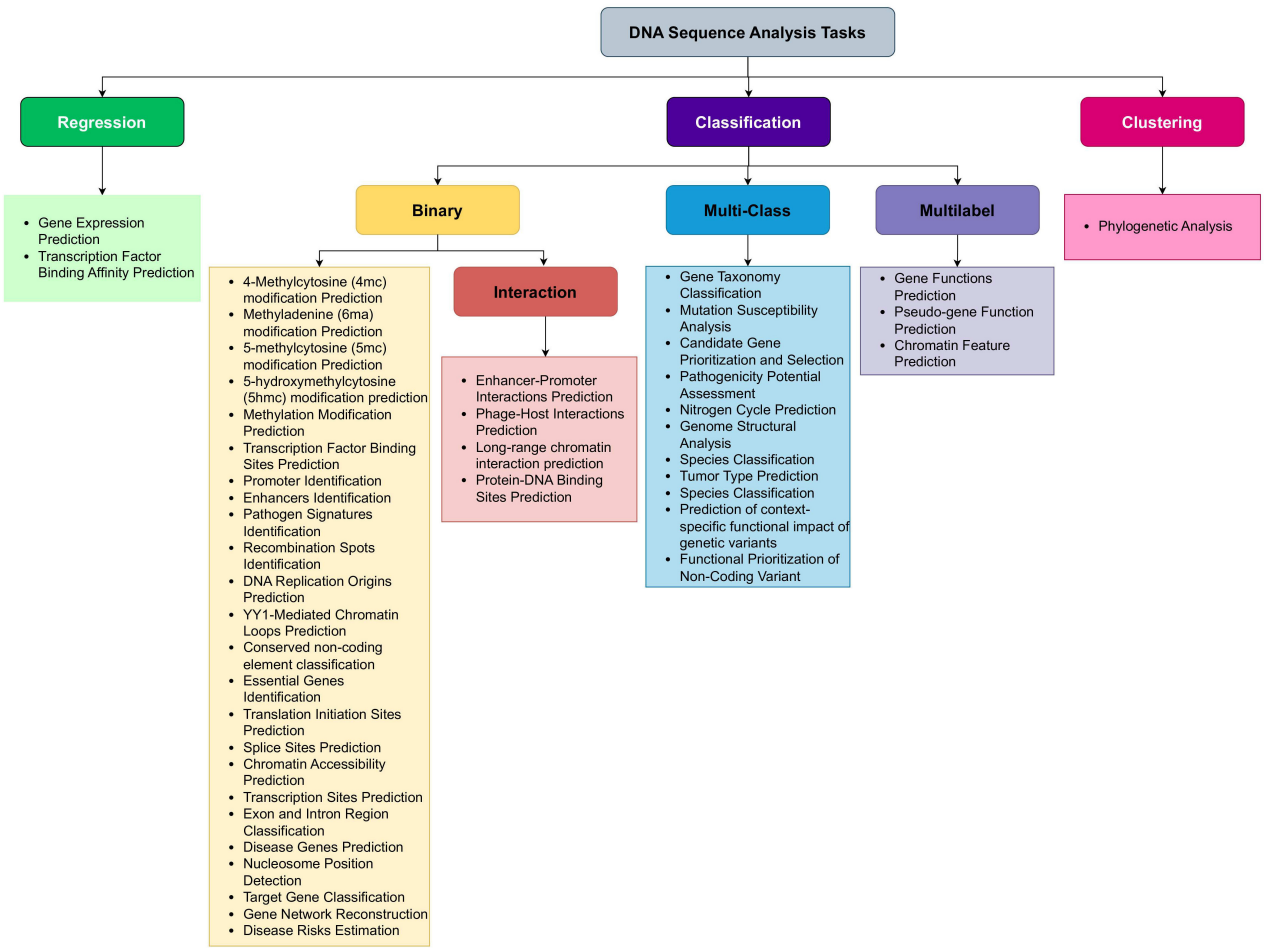
DNA sequence analysis task representation for computer scientist perspective.

While Section 3 presents a biologically motivated categorization of DNA sequence analysis tasks, this section reframes these same tasks from a computational perspective. This dual categorization approach (biological and computational) aims to facilitate interdisciplinary understanding between life scientists and AI researchers. With the influx of biological data and rise of AI, researchers are increasingly applying AI in diverse areas of molecular biology. Development of large scale AI applications requires a good understanding of variety of sequence analysis tasks. However, there exist a huge domain gap between computer scientists and molecular biologists. Molecular biologists know the need, biological importance, and pharmaceutical worth of different sequence analysis tasks. However, they do not know which machine or deep learning models are most appropriate to use to either replace or complement experimental work. Similarly, computer scientists know which Artificial Intelligence predictive pipeline can potentially perform better with specific type of data; however, they do not know the nature of biological sequence analysis tasks. For instance, DNA sequence analysis tasks such as gene function prediction, gene network reconstruction, gene expression prediction, and disease risk estimation can be challenging for computer scientists to grasp. However, a comprehensive literature review that explains the basics of these tasks can bridge this gap. For example, gene function prediction is a multi-label classification tasks, gene expression prediction is a regression task, while gene network reconstruction and disease risk estimation are binary classification tasks. With this foundational understanding, computer scientists can more easily develop predictive pipelines for these binary, multi-label classification, and regression tasks. To empower AI experts, we have presented 44 DNA sequence analysis tasks in computer scientist language in Figure 4. A simple look on Figure 4 reveals that nature of DNA sequence analysis tasks can be categorized into three primary types: regression, clustering, and classification where classification can be further divided into three secondary types: binary classification, multi-class classification, and multi-label classification. Let us mathematically formulate the possible natures of DNA sequence analysis tasks.

In binary classification, researchers aim to predict the outcome of a binary variable (0 or 1). Given a dataset with features *X* ∈ ℝ^*nxd*^, binary labels *y* ∈ 0, 1, and training dataset (*x*_1_, *y*_1_), (*x*_2_, *y*_2_), …, our goal is to learn a decision function *f*:*X* → *Y* that maps inputs to binary outputs 0, 1 on the basis of hypothesis function *h*(*x*) learned from the training data.


(1) $$f(x)=\{\begin{array}{ll} 1 & ifh(x)\;⩾\;0.5\\ 0 & otherwise \end{array}$$

In multi-class classification, researchers aim to predict the outcome from more than two classes. Specifically, given a dataset having sequences *X* ∈ ℝ^*nxd*^, labels *y* ∈ 1, 2, …, *K* where *K* is the number of classes, and training dataset (*x*_1_, *y*_1_), (*x*_2_, *y*_2_), …, (*x*_*n*_, *y*_*n*_) where *x*_*i*_ ∈ *X* and *y*_*i*_ ∈ *Y*, our goal is to learn a decision function *f*:*X*→*Y* that assigns inputs to one of the classes.


(2) $$\begin{array}{l} f\left(x\right)=argmax_{k}h_{k}\left(x\right) \end{array}$$

where *h*_*k*_(*x*) is the hypothesis function for class *k* learned from the training data. On the other hand, in multi-label classification, each input can be assigned to multiple classes simultaneously. Given a dataset with features *X* ∈ ℝ^*nxd*^, labels *y* ∈ 1, 2, …, *K* where *K* is the number of classes, and training dataset (*x*_1_, *y*_1_, *y*_2_, ..), (*x*_2_, *y*_1_, *y*_4_, …), …, (*x*_*n*_, *y*5, *y*_*n*_, ….) where *x*_*i*_ ∈ *X* and *y*_*i*_ ∈ *Y*, our goal is to learn a decision function *f*:*X* → 0, 1^*K*^ that assigns inputs to multiple classes simultaneously using hypothesis function *h*_*k*_(*x*) for class *k* learned from the training data.


(3) $$\begin{array}{l} f\left(x\right)=\left(h_{1}\left(x\right),h_{2}\left(x\right),...,h_{K}\left(x\right)\right) \end{array}$$

Furthermore, in regression, researchers goal is to predict a continuous outcome variable. Given a dataset with sequences *X* ∈ ℝ^*nxd*^, labels *y* ∈ ℝ, and training dataset (*x*_1_, *y*_1_), (*x*_2_, *y*_2_), …, (*x*_*n*_, *y*_*n*_) where *x*_*i*_ ∈ *X* and *y*_*i*_ ∈ *Y*, our goal is to learn a function *f*:*X* → ℝ that predicts continuous outputs using hypothesis function *h*(*x*) learned from the training data.


(4) $$\begin{array}{l} f\left(x\right)=h\left(x\right) \end{array}$$

In clustering, the goal is to group similar data points into same clusters. Given a dataset with data points *X* = *x*_1_, *x*_2_, …, *x*_*n*_, where each $x_{i}\in {\mathbb{R}}^{d}$, our goal is to find a partition of the data into clusters *C* = *C*_1_, *C*_2_, …, *C*_*K*_. This is done on the basis of a distance metric *d*(*x*, μ_*c*_) between data point *x* and the centroid μ_*c*_ of cluster *c*.


(5) $$\begin{array}{l} f\left(x\right)={\text{argmin}}_{c}d\left(x,\mu_{c}\right) \end{array}$$

## 5 DNA sequence analysis databases

This section provides a comprehensive overview of various databases employed to develop benchmark datasets for development of AI-based applications for 44 distinct DNA sequence analysis tasks. A total of 45 DNA sequence databases have been identified from 127 existing studies. Among these, 36 databases are publicly accessible, while the remaining 9 databases are either inaccessible or no longer exist. To ease the lives of researchers and practitioners, Table 1 summarizes accessible databases in terms of their release year, types of inherent genetic data (DNA, RNA, protein), details of species and organisms, statistics of raw sequences, and supported data formats.

**Table 1:** Summary of publicly accessible biological databases, their inherent data types, species diversity, and statistics of raw sequences related to different genomic and proteomic data.

**Database name**	**Release date**	**Type of data (DNA/RNA/ Protein)**	**Organism name**	**Species name**	**Sequences statistics**	**Data format**
Descartes	2020	DNA, RNA	Animals	Mus musculus, Homo sapiens	Human Gene Expression During Development: 4M Cells, 121 Tissues, 15 Organs; Human Chromatin Accessibility During Development: 720K Cells, 53 Tissues, 15 Organs; Mouse: ~2M Cells, 61 Embryos	.RDS
PPD	2020	DNA, RNA	Bacteria, Archaea	63 species	129,148 Promoter Sequences, 63 Species with 74 strains	.csv
EnhancerAtlas 2.0	2019	DNA	Animals, Bacteria	Homo sapiens, Mus musculus, Drosophila melanogaster, Caenorhabditis elegans, Danio rerio, Rattus norvegicus, Gallus gallus, Sus scrofa, Saccharomyces cerevisiae	13,494,603 Enhancers, 586 tissue	.csv, BED, R Object
DREAM Base	2018	DNA, RNA, Protein	Animals	Homo sapiens	scRNA-Seq Data: 93,3704 cells; RNA-Seq Data: ~18,196; ChiP-Seq Data: ~10,000; RNA Modification Data: ~500; ribo-Seq Data: 1,570; DNase-Seq Data: 599; CLIP-Seq Data: 568	.excel, .txt
EmExplorer database	2018	DNA, Protein	Animals	Bos taurus, Homo sapiens, Mus musculus, Rattus norvegicus, Sus scrofa	158,000 items that contain more than 32,000 development-related Genes under 306 related pathways	.txt
COSMIC	2018	DNA	Animals	Homo sapiens	Total Genomic variants = 24,599,940; Genomic non-coding variants = 16,748,366,406; Genomic mutations within Exons = 768; Genomic mutations within Intronic and other intragenic regions = 9,217,664; Samples = 1,531,613; Fusions = 19,428; Gene expression variants = 9,215,470; Differentially Methylated CpGs = 7,930,489	.FASTA, .tsv
DisGeNet	2015	DNA	Animals	Homo sapiens	1,134,942 GDAs between 21,671 Genes, 30,170 diseases and traits; 369,554 VDAs between 194,515 variants and 14,155 diseases and traits	.txt, RDF, SQL Dump
genomAD	2014	DNA	Animals	Homo sapiens	730,947 Exomes, 76,215 whole Genomes	VCF, Hail Table
ClinVar	2013	DNA, RNA, Protein	Animals	Homo sapiens	Records = 4391341, Total Genes = 92225	.xml, VCF, .tsv
HOCOMOCO Human v11 database	2013	DNA	Animals	Mus musculus, Homo sapiens	1,443 TF binding models including secondary motif subtypes for 949 human TFs and 720 Mouse orthologs	PWM, PFM, PCM, Flat text files
DeOri	2012	DNA	Animals	Homo sapiens, Mus musculus, Arabidopsis thaliana, Kluyveromyces lactis, Schizosaccharomyces pombe, Drosophila melanogaster	189,743 entries	.FASTA
BioLip	2012	DNA, RNA, Protein	Animals	Homo sapiens	873,925 Entries, 448,816 regular ligands, 191,485 mental ligands, 37,492 Peptide ligands, 43,448 DNA ligands, 152,684 RNA ligands, 873,925 binding affinity data, 451,485 Protein receptors	.FASTA
DeOri6.0	2011	DNA	Animals, Plants, Fungi	17 species	189,740 eukaryotic replication origins	.FASTA
GWAS	2008	DNA	Animals	Homo sapiens	146,394 TASs	.tsv, OWL/RD
Broad DepMap	2008	DNA	Animals	Homo sapiens	2,000 Human cancer cell lines	.csv, .txt
CCLE	2008	DNA, RNA, Protein	Animals	Homo sapiens	1,019 RNA cell lines, 954 microRNA expression profiles, 899 Protein lines, 897 Genome-wide histone modifications, 843 DNA methylation, 329 whole Genome Sequencing, 326 whole exome Sequencing	.csv
GENCODE	2006	DNA	Animals	Homo sapiens, Mus musculus	Homo sapiens: Total genes = 63,086, Total transcripts = 254,070, Total distinct Translations = 65,650; Mus musculus: Total Genes = 57,132, Total Transcripts = 149,138, Total distinct Translations = 44,819	.txt
Consensus Coding Sequence Database	2005	DNA	Animals	Homo sapiens, Mus musculus	35,608 CCDS IDs that correspond to 19,107 Genes, with 48,062 Protein Sequences	.FASTA
MSigDB	2005	DNA	Animals	Homo sapiens, Mus musculus	8,380 Gene set	.gct, .res, .pcl, .txt, .cls, .gmx, .gmt, .grp, .xml, .chip, .rnk
Gene Ontology	2004	DNA	Animals, Bacteria, Fungi, Plants	Escherichia coli, Homo sapiens, Oryza sativa, Saccharomyces cerevisiae, Schizosaccharomyces pombe, Mus musculus, many more	Annotated Gene products = 1,536,921; Annotated Species = 5,409; Annotated Species with over 1,000 annotations = 183	OBO, OWL, GAF, GPAD, GPI, JSON
JASPAR	2004	DNA, RNA, Protein	Fungi, Insects, Nematoda, Plants, Urochordata, Vertebrata	34 species	4279 Profiles	.txt
Database of Essential Genes	2004	DNA, RNA, Protein	Bacteria, Archaea, Eukaryotes	51 species	53,885 essential Genes, 786 essential non-coding Sequences	.csv, DAT
ENCODE	2003	DNA, RNA, Protein	Animals	Homo sapiens, Mus musculus	_	.FASTA, BAM, BigWig, BED, VCF
DataBase of Transcriptional Start Sites	2002	DNA	Animals	Homo sapiens, Mus musculus	491M TSS tag Sequences from a total of 20 tissues and 7 cell cultures	.FASTA, .csv, .xlsx, .txt
MGC	2001	DNA	Animals	Mus musculus, Homo sapiens, Rat, Bovine	Total MGC Full ORF Clones: Homo sapiens: 29,818; Mus musculus: 27,285; Rat: 6,763; Bovine: 9,104; Non-redundant Genes: Homo sapiens: 17,592; Mus musculus: 17,701; Rat: 6,486; Bovine: 8,724	_
GEO	2000	DNA, RNA, Protein	Animals	20 species	Samples = 7,209,691	SOFT, MINiML, .txt
Exon-Intron Database	1999	DNA, RNA	Animals, Plants	Homo sapiens, Mus musculus, Drosophila melanogaster, C. Elegans, S. Pombe	42,460 Genes (243,589 Exons)	.FASTA
Ensembl	1999	DNA, RNA, Protein	Animals	Homo sapiens, Mus musculus, Danio rerio, Sus scrofa	Genomes = 44,048, Ensembl Fungi = 1,014 Genomes, Ensembl Metazoa = 78 Genomes (Invertebrate species), Genomes for vertebrate Species = 236, Ensembl Plants = 67 Genomes, Ensembl Protists = 237 Genomes	.FASTA, GTF, GFF, MySQL Dump
RegulonDB	1998	DNA, RNA, Protein	Bacteria	Escherichia coli	4,748 Genes, 2,590 Operons, 287 Regulons, 3,718 Transcription Unit, 4050 Promoters	.tsv, .csv
EPD2	1998	DNA	Animals, Plant, Fungi, Protists	139 species	4,806 Promoters	.FASTA, EMBL
dbSNP	1998	DNA	Human	Homo sapiens	Nearly 2 billion submissions representing more than 675 million distinct variants; 23.7 million refSNP entries (14.5 million validated)	ASN.1, FASTA, XML
KEGG	1995	DNA, RNA, Protein	Animals, Plants, Fungi, Protists, Bacteria, Archaea	Euryarchaeota Candidatus, Thermoplasmatota, Thermoproteota, Chordata, Echinodermata, Hemichordata, Ascomycota, Basidiomycota, Atribacterota Candidatus, Saccharibacteria	Genes = 53,674,741, Addendum Proteins = 4,181, Viral Genes=688,823, Viral mature Peptides = 377	KGML, .FASTA, .txt
NCBI	1988	DNA, RNA, Protein	Multiple organisms	Multiple species	Hub of databases	.FASTA, XML
Eukaryotic Promoter Database	1986	DNA	Animals, Plants, Fungi, Invertebrates	Homo sapiens, Macaca mulatta, Mus musculus, Rattus norvegicus, Gallus gallus, Canis familiaris, Drosophila melanogaster, Apis mellifera, Danio rerio, Caenorhabditis elegans, Arabidopsis thaliana; Zea mays, Saccharomyces cerevisiae, Schizosaccharomyces pombe, Plasmodium falciparum	192,586 Promoters, 163,676 Genes	.FASTA, EMBL
GenBank	1982	DNA	Animals, Archaea, Bacteria, Fungi, Plants, Virus	557000 species	3,213,818,003,787 Bases, 250,803,006 Sequences	.gb
OMIM	1960	DNA	Animals	Homo sapiens	17,290 Gene descriptions, 18 Gene and Phenotypes, 8,361 Phenotype description, 1,736 Phenotypes suspected mendelian	.txt

A holistic view of the Table 1 reveals that 12 databases provide RNA and protein sequences as well in addition to providing DNA sequences. As word embeddings methods and large language models are trained in unsupervised fashion and when they are trained on large sequence data usually, they produce better representations. To efficiently train word embedding methods and large language models, raw data can be acquired from these databases. To facilitate researchers, we have categorized 36 databases into three different categories on the basis of volume of raw sequences: low sequence facilitators, medium sequence facilitators, and high sequence facilitators. Specifically, 13 low sequence facilitators, namely, HOCOMOCO Human v11 database (182), Consensus Coding Sequence Database (183), MSigDB (184), Broad DepMap (185), JASPAR (186), Database of Essential Genes (187), ENCODE (188), MGC (189), Exon-Intron Database (190), Ensembl (191), RegulonDB (192), EPD2 (193), offer up to 100,000 DNA sequences each, while 9 medium sequence facilitators, namely, PPD (194), DREAM (195), EmExplorer database (196), GenomAD (197), DeOri (198), BioLip (199), DeOri6.0 (198), GWAS (200), Eukaryotic Promoter Database (193), provide up to 1 million DNA sequences. In contrast, 13 high sequence facilitators such as Descartes (201), EnhancerAtlas 2.0 (202), COSMIC (203), DisGeNet (204), ClinVar (205), CCLE (206), GENCODE (207), Gene Ontology (208), DataBase of Transcriptional Start Sites (209), GEO (210), KEGG (211), NCBI (212), GenBank (213), and dbSNP (214, 215) offer more than 1 million DNA sequences each. These databases predominantly house DNA sequences from a diverse array of species, including humans, mice, plants, bacteria, and fungi. A comprehensive analysis reveals that approximately 22 databases, namely, Descartes (201), DREAM (195), EmExplorer database (196), COSMIC (203), DisGeNet (204), GenomAD (197), ClinVar (205), HOCOMOCO Human v11 database (182), DeOri (198), BioLip (199), GWAS (200), Broad DepMap (185), CCLE (206), GENCODE (207), Consensus Coding Sequence Database (183), MSigDB (184), ENCODE (188), DataBase of Transcriptional Start Sites (209), MGC (189), GEO (210), Ensembl (191), and OMIM (216), focus on animal DNA sequences, 4 databases including PPD (194), Database of Essential Genes (187), RegulonDB and (192) on bacterial sequences, and JASPAR (186) on plant DNA sequences. EnhancerAtlas 2.0 (202) is the only database that facilitates with both animal and bacterial DNA sequences, while 4 databases namely DeOri6.0 (198), Exon-Intron Database (190), EPD2 (193), and Eukaryotic Promoter Database (193) focus on animal and plant DNA sequences, whereas Gene Ontology (208), KEGG (211), and GenBank (213) provide DNA sequences for animal, plant, and bacteria. In addition, sequences from other organisms such as eukaryotes, invertebrates, fungi, and various microorganisms are also well-represented. Some databases encompass a broad spectrum of species. For instance, the EDP2 (193) database includes genomics data for 139 species, GenBank (213) houses sequences for 557,000 species, and PPD (194) has genomics data of 63 species.

Moreover, Table 1 includes data formats utilized by various databases to manage and provide access to DNA sequences. TXT and FASTA format are universally accepted by almost all DNA sequence analysis programs. Each entry in both format types contains at least two lines: First line or header includes accession number, species name, or identification details, while next line contains nucleotide sequences. CSV and TSV are text-based formats in which values in rows are separated by commas or tabs, respectively. In both file formats, first row specifies headers which defines names of columns (“SeqID”, “SeqName”, “Type”, “Function”) and subsequent rows represent data. In VCF format, first row specifies headers which defines names of columns, but this format is specifically used to store genetic variation data including single nucleotide polymorphisms (SNPs), insertions, deletions, and structural variants. In addition, XLSX formats represent complex datasets that contain information computed with various formulas across multiple columns, whereas EMBL format includes structured sections for sequence data, feature annotations (genes and other biological features), organism information, references, and other details. An extensive analysis of Table 1 reveals that most widely used data formats are FASTA, TXT, CSV, XLSX, and EMBL in DNA sequence analysis.

A rigorous analysis of Table 1 reveals that out of 36 publicly accessible databases, several key categories of data emerge. Four databases, namely, Broad DepMap (185), genomAD, COSMIC, and MGC, provide data for DNA functional analysis tasks such as prediction of context-specific functional impact of genetic variants and conserved non-coding element classification. Seven databases, namely, BioLip, HOCOMOCO Human v11, GWAS, EnhancerAtlas 2.0, DataBase of Transcriptional Start Sites, Exon-Intron Database, and Eukaryotic Promoter Database, offer data on gene expression regulation. Three databases, namely, PPD, CCLE, and EmExplorer, focus on DNA modification data including methylcytosine and methyladenine modifications. In addition, DeOri, Descartes, DeOri6.0, and JASPAR provide information on gene structure and stability, including chromatin accessibility prediction, YY1-mediated chromatin loop identification, and DNA replication origins identification. GENCODE, Consensus Coding Sequence Database, MSigDB, Gene Ontology, DisGeNet, Database of Essential Genes, KEGG, and NCBI offer comprehensive gene analysis data. Furthermore, eight other databases, namely, EPD, ENCODE, RegulonDB, GEO, Ensembl, ClinVar, GenBank, and OMIM, provide a range of data on gene expression regulation, DNA modification prediction, genome structure and stability, DNA functional analysis, disease information, and gene analysis.

## 6 DNA sequence analysis benchmark datasets

The quality and quantity of datasets utilized in AI-driven DNA sequence analysis applications are vital determinants of their effectiveness and functionality. This section aims to provide a comprehensive overview of datasets relevant to 44 distinct DNA sequence analysis tasks. Overall, these datasets fall into two primary categories: publicly available datasets and in-house datasets. This categorization serves to illuminate the significance of dataset accessibility and its implications for the advancement of AI-driven DNA sequence analysis. Specifically, publicly available datasets are accessible to the wider research community and are commonly employed in the development of AI-based predictive models. They serve as foundational resources that facilitate the advancement of AI-driven DNA sequence analysis pipelines by ensuring accessibility, reusability, and transparency in research endeavors. Furthermore, the utilization of publicly available datasets fosters collaboration and knowledge exchange within the scientific community, thereby contributing to the overall progress of the field. In contrast, in-house datasets are proprietary in nature and are developed within specific research laboratories or institutions. These datasets often contain sensitive data tailored to particular research objectives. As in-house datasets cannot be shared publicly, their proprietary nature may limit broader access, reproducibility, and applicability of findings.

Rigorous assessment of **127** existing studies reveals that a total of **242** benchmark datasets related to 44 distinct DNA sequence analysis tasks are constructed or acquired from existing literature. Specifically, among these 242 benchmark datasets, **199** are publicly available and **43** are in-house datasets. Table 2 provides the distribution of public and in-house datasets for 44 distinct DNA sequence analysis tasks. It provides information about which of these datasets are used by word embeddings, large language models, nucleotide composition, and positional information-based predictive pipelines.

**Table 2:** Overview of 199 public and 43 in-house datasets used across 44 different DNA sequence analysis tasks.

**Task name**	**Task type**	**Datasets used in language models**		**Datasets used in word embeddings**		**Datasets used in other methods**	
		**Public**	**In-house**	**Public**	**In-house**	**Public**	**In-house**
DNA replication origins identification	Binary classification	_	Gao et al. *(A. thaliana)* (25)	_	Wu et al. datasets (*S. cerevisiae* Dataset, *S. pombe* Dataset, *K. lactis* Dataset, *P. pastoris* Dataset) (329)	_	_
Nucleosome position detection	Binary classification	Gangi et al. datasets (CE, DM, YS, HM, DM-5U, DM-PM, DM-LC, HM-5U, HM-LC, HM-PM, YS-PM) (330)	_	_	_	_	_
Chromatin accessibility prediction	Binary classification	DeepSEA datasets (TF, DHS) (32), DNase-Seq experiment data (31)	_	_	_	_	_
YY1-Mediated chromatin loops prediction	Binary classification	_	_	Dao et al. DeepYY1 datasets (HCT116, K562) (39)	_	Zhang et al. DeepYY1 datasets (HCT116, K562) (38)	_
Genome structure analysis	Multi-class classification	NCycDB dataset (27)	_	_	_	_	_
Chromatin feature prediction	Multi-label classification	Logo919 (34), Logo2002 (34), Logo3357 (34)	_	_	_	_	_
Long-range chromatin interaction prediction	Interaction	Chip-seq dataset (36)	_	_	_	_	_
Enhancers identification	Binary classification	Liu et al. dataset (57), Liao et al. datasets (HEK293, NHEK, K652, GM12878, HMEC, HSMM, NHLF, HUVEC) (58)	_	Liu et al. dataset (57)	_	DiseaseEnhancer (55), EnDisease (55), CancerEnD (55)	_
Promoter identification	Binary classification	Yang et al. dataset (34), Ji et al. dataset (90), Xiao et al. dataset (331)	_	Wang et al. dataset [*H. Sapiens*-I (TATA-containing), *H. Sapiens-II* (TATA-less), *R. Norvegicus-I* (TATA-containing), *R. Norvegicus-II* (TATA-less), *D. melanogaster-I* (TATA-containing), *D. melanogaster-II* (TATA-less), *Z. mays-I* (TATA-containing), *Z. mays-II* (TATA-less)] (236), Zhang et al. dataset (K562, GM12878, HeLa-S3, HUVEC) (76), Xiao et al. dataset (331)	_	Yang et al. dataset (34), Xiao et al. (331)	_
Enhancer-promoter interactions prediction	Interaction/binary classification	Yang et al. datasets (FoeT, Mon, nCD4, tB, tCD4, tCD8) (249)	_	Whalen et al. dataset (GM12878, HUVEC, HeLa-S3, IMR90, K562, NHEK) (329)	_	Whalen et al. dataset (GM12878, HUVEC, HeLa-S3, IMR90, K562, NHEK) (329)	Zhang et al. (GM12878 cell line, HeLa cell line) (332)
Transcription sites prediction	Binary classification	Clauwaert et al. dataset (50)	_	_	_	_	_
Transcription factor binding sites prediction	Binary classification	ChIP-Seq dataset (94), TSSs dataset (91), 497 TF ChIP-Seq dataset (90), 690 ChIP-Seq (51)	_	Shen et al. datasets (A549 dataset, MCF-7 Dataset, H1-HESCDataset, HUVEC dataset) (92)	_	_	_
Transcription factor binding affinity prediction	Multi-class classification	Weirauch et al. dataset (PBM Dataset), Jolma et al. dataset (HT-SELEX Dataset) (52)	_	_	_	_	_
Protein-DNA binding sites prediction	Interaction/binary classification	690 ChIP-Seq dataset (96), Patiyal et al. dataset (53), Xia et al. (dataset 2) (53), Liu and Tian (dataset 1, dataset 2) (95)	_	_	_	_	_
Splice sites prediction	Binary classification	Wang et al. dataset (100), Ji et al. dataset (93)	_	_	_	Splice-junction gene sequence dataset (97), Degroeve et al. (98), Liu et al. Datasets [*O. sativa* (Acceptor, Donor), *A. Thaliana* (Acceptor, Donor), *H. sapiens* (Acceptor, Donor)] (99)	_
Translation initiation sites	Binary classification	Clauwaert et al. dataset (50)	_	_	_	Kalkatawi et al. TIS (101)	_
Essential genes identification	Binary classification	Ma et al. datasets (*S. cerevisiae, E. coli, H. sapiens, D. melanogaster*) (109)	_	Ma et al. datasets (*S. cerevisiae, E. coli, H. sapiens, D. melanogaster*) (109), Campos et al. datasets (*D. melanogaster, M. maripaludis, H. sapiens, C. elegans*) (333), Zhang et al. (106), Xiao et al. dataset (107)	_	Campos et al. datasets (*D. melanogaster, M. maripaludis, H. sapiens, C. elegans*) (333), Sharma et al. (104)	_
Disease genes prediction	Binary classification	Nunes et al. dataset (110)	_	_	_	_	_
Pseudogene function prediction	Interaction/binary classification	_	_	Fan et al. dataset (CC, MF, BP) (111)	_	_	_
Target gene classification	Multi-class classification	_	_	Arango et al. dataset (113)	_	_	_
Candidate gene prioritization & Selection	Multi-class classification	_	Toufiq et al. dataset (114)	_	_	_	_
Gene functions prediction	Multi-label classification	Hu et al. (Human gene set from gene ontology) (112)	_	GTEx (CC, MF, BP) (111)	_	_	_
Gene expression prediction	Regression	Reddy et al. dataset (Jurkat, K-562, THP-1) (334)	_	Al Taweraqi et al., dataset (103)	_	_	_
Gene taxonomy classification	Multi-class classification	Mock et al. dataset (123)	_	Verma et al. dataset (121)	CAMI2 Airway dataset (122)	_	_
Gene network reconstruction	Multi-class classification	_	_	SynTReN dataset (128) DREAM5 dataset (128)	_	_	Pio et al. dataset (126), Schaffter et al. datasets (DREAM4 10, DREAM4 100) (127), Jozefczuk et al. datasets (*E.coli* cold, *E.coli* head, *E.coli* oxidative) (127)
4mc-Methyl-cytosine modification prediction	Binary classification	Xu et al. datasets (*C. elegans, D. Malenogaster, A. Thaliana, E.coli, G. subterraneus, G. pickeringii*) (141), Chen et al., datasets (*C. elegans, D. Malenogaster, A. Thaliana, E.coli, G. subterraneus, G. pickeringii*) (135)	_	Khanal et al. datasets (*F. vesca, R. chinensis*) (138), Zulfiqar et al. dataset (136), Zeng et al. dataset (137)	_	Khanal et al. (138), Chen et al., Datasets (*C. elegans, D. Malenogaster, A. Thaliana, E.coli, G. subterraneus, G. pickeringii*) (135)	Manavalan et al. (139)
5mc-Methyl-Cytosine modification prediction	Binary classification	Wang et al. dataset (282), Stanojevic et al. dataset (GM24385, NA12878, NA19240, H1ESc, K562, HX1) (152)	_	Wang et al. dataset (282), *Hyb*_2021 (*C. elegans, D. Malenogaster, A. Thaliana, E.coli, G. subterraneus, G. pickeringii*) (142)	_	Xu et al. (141), Rao Zeng et al. (140), Saha et al. (135)	Nguyen-Vo et al. (139)
5hmc-hydroxy-methylcytosine modification prediction	Binary classification	_	_	Lv et al. dataset (*M. musculus, H. sapiens*) (156)	_	_	_
6mA-methyladenine modification prediction	Binary classification	Abbas et al. dataset (*A. thaliana, H. sapiens, M. musculus, S. cerevisiae*) (335), DNA 6 mA dataset (281)	_	Lv et al. dataset (156)	_	Zhou et al. dataset (*A. thaliana, C. elegans, C. equisetifolia, D. melanogaster, F. vesca, H. sapiens, R. chinensis, S. cerevisiae, T. thermophile*, Xos. BLS256) (148), Fan et al. dataset (1, D.malenogaster, 3, 4, 5) (149)	_
Methylation modification prediction	Binary classification	Lv et al. dataset 6mA (*T. thermophile, A. thaliana, H. sapiens*, Xos. BLS256, *D. melanogaster, C. elegans, C. equisetifolia, S. cerevisiae*, Tolypocladium, *F. vesca, R. chinensis*) 5hmC (*M. musculus, H. sapiens*) 4mC (*F. vesca, Tolypcladium, S. cerevisiae, C. equisetifolia*) (156)	_	_	_	_	_
Conserved non-coding element classification	Binary classification	_	_	_	Polychronopoulos et al. dataset (163)	_	_
Functional prioritization of non-coding variants	Multi-class classification	Logo919 (34) Logo2002 (34) Logo3357 (34)	_	_	_	_	_
Exon & Intron region classification	Binary classification	Akalin et al. dataset (164)	_	_	_	_	_
Recombination spots identification	Binary classification	_	_	Liu et al. dataset (165)	_	_	_
Species classification	Multi-class classification	Mouse enhancers (44) Coding vs. intergenomic (44) human vs. worm (44) Human enhancers cohn (44) human enhancers ensembl (44) Human Regulatory (44) human nontata promoter (44) human OCR ensembl ()	_	_	_	_	_
Prediction of context-specific functional impact of genetic variants	Multi-class classification	eQTLs dataset (36)	_	_	_	_	_
Nitrogen cycle prediction	Multi-class classification	_	NCycDB (27)	_	_	_	_
Pathogen signature identification	Binary classification	_	_	_	DS500 dataset (171)	_	_
Phage-host interactions prediction	Interaction/binary classification	_	_	ESKAPE dataset (177), Wang et al. dataset (176)	_	Qiu et al. dataset (Kingdom, Phylum, Class, Order, Family, Genus) (175)	_
Mutation susceptibility analysis	Multi-class classification	_	_	_	Yilmaz et al. dataset (Human, Mouse) (173)	_	_
Tumor type prediction	Multi-class classification	TCGA pan-cancer dataset (180)	_	_	_	_	_
Pathogenicity potential assessment	Multi-class classification	_	E-K12 (27), CARD-A (27), CARD-D (27), CARD-R (27), VFDB (27), ENZYME (27), PATRIC (27), NCycDB (27)	_	_	_	_
Phylogenetic analysis	Clustering	_	_	_	Ren et al. datasets (111)	_	_
Disease risks estimation	Binary classification	HSCR-RET (90), HSCR-RET-Long (90)	_	_	_	_	_

For each DNA sequences analysis task, public and in-house datasets are distributed as DNA Replication Origins Identification (0, 5), Nucleosome Position Detection (11, 0), Chromatin Accessibility Prediction (2, 0), YY1-Mediated Chromatin Loop Prediction (4, 0), Genome structure analysis (0, 1), Chromatin Feature Prediction (3, 0), Long-range chromatin interaction prediction (1, 0), Enhancers Identification (12, 0), Promoter Identification (15, 0), Enhancer-Promoter Interactions Prediction (18, 2), Transcription Site Prediction (1, 0), Transcription Factor Binding Site Prediction (4, 4), Transcription Factor Binding Affinity Prediction (2, 0), Protein-DNA Binding Site Prediction (5, 0), Splice Site Prediction (10, 0), Translation Initiation Sites (1, 1), Essential Gene Identification (6, 5), Disease Gene Prediction (1, 0), Pseudogene Function Prediction (3, 0), Target Gene Classification (1, 0), Candidate Gene Prioritization/ Identification (0, 1), Gene Function Prediction (4, 0), Gene Expression Prediction (4, 0), Gene Taxonomy Classification (2, 1), Gene Network Reconstruction (2, 6), 4mc-Methylcytosine Site Prediction (16, 0), 6mA-Methyladenine Site Prediction (5, 0), 5mc-Methylcytosine Site Prediction (24, 1), 5hmc-Methylcytosine Site Prediction (2, 0), Methylation Site Prediction (17, 0), Conserved Non-Coding Elements Classification (0, 1), Functional Priorizitation of non-coding variants (3, 0), Exon and Intron Region Classification (0, 1), Recombination Spots Identification (1, 0), Species Classification (8, 0), Prediction of context-specific functional impact of genetic variant (1, 0), Nitrogen Cycle Prediction (0, 1), Pathogen Signatures Identification (0, 1), Phage-Host Interactions Prediction (8, 0), Mutation Susceptibility Analysis (0, 2), Tumor Type Prediction (1, 0), Pathogenicity Potential Assessment (0, 8), Phylogenetic Analysis (0, 1), and Disease Risks Estimation (2, 0). First entry in brackets refers to count of public datasets, and second entry indicates total number of in-house datasets for a particular task. For example, in “Essential Gene Identification (6, 5)” task, 6 refers to public datasets while 5 represents in-house datasets.

A holistic view of Table 2 reveals 110 public and 18 in-house datasets are employed to develop both word embeddings and language models based predictive pipelines for 12 DNA sequence analysis tasks, namely, DNA replication origins identification, enhancers identification, promoters identification, enhancer-promoter interaction prediction, transcription factor binding site prediction, essential gene identification, gene function prediction, gene expression prediction, gene taxonomy classification, 4mC-methyl cytosine modification prediction, 5mC-methl cytosine modification, and 6mA-methyl modification prediction. Notably, both types of predictive pipelines have utilized 1 common dataset to evaluate the performance of predictive models developed for three tasks, namely, enhancer identification, essential gene identification, and 5mC-methyl cytosine modification prediction.

Furthermore, 112 public and 15 in-house datasets are used to develop both word embedding and nucleotide compositional and positional information-based predictive pipelines for 11 DNA sequence analysis tasks including essential gene identification, gene network reconstruction, 4mC-methyl cytosine modification prediction, 5mC-modification prediction, 6mA-methyl adenine modification prediction, and phage-host interaction prediction. However, both predictive pipelines have used 9 common pubic dataset for only three tasks. Specifically, six public datasets for enhancer-promoter interactions prediction, one public data for essential gene identification, and two public datasets for 4mC-Methyl cytosine modification prediction are commonly employed by both predictive pipelines.

Moreover, Table 2 highlights that 107 public and 9 in-house datasets are utilized by 9 DNA sequence analysis tasks, namely, enhancers identification, promoters identification, enhancer-promoter interaction prediction, splice site prediction, translation initiation sites identification, essential gene identification, 4mC-methyl cytosine modification prediction, 5mC-methl cytosine modification, and 6mA-methyl modification prediction for developing both language models and nucleotide compositional and positional information-based predictive pipelines. Merely, 7 public datasets are used commonly by both predictive pipelines for two tasks: one for promoter identification and six for 4mC-methyl cytosine modification prediction.

Although all three different types of representation learning-based predictive pipelines are employed across six different DNA sequence analysis tasks, namely, enhancers identification, promoters identification, enhancer-promoter interaction prediction 4mC-methyl cytosine modification prediction, 5mC-methl cytosine modification, and 6mA-methyl modification prediction, only on one task, namely, promoters identification, all three kinds of predictive pipelines are evaluated on one common dataset. These statistics reveal that researchers have focused on creating new datasets for each kind of predictive pipelines instead of using existing datasets. Consequently, this domain lacks a fair performance comparison between different kinds of predictive pipelines.

## 7 A brief look on representation learning methods and predictors used in DNA sequence analysis predictive pipelines

This section dives into 12 most commonly used word embedding approaches, 8 large language models, 9 machine learning, 8 deep learning, and 3 statistical algorithms that are used in development of predictive pipelines for 44 different DNA sequence analysis tasks.

### 7.1 DNA sequence representation learning using word embeddings

**Figure 5:**
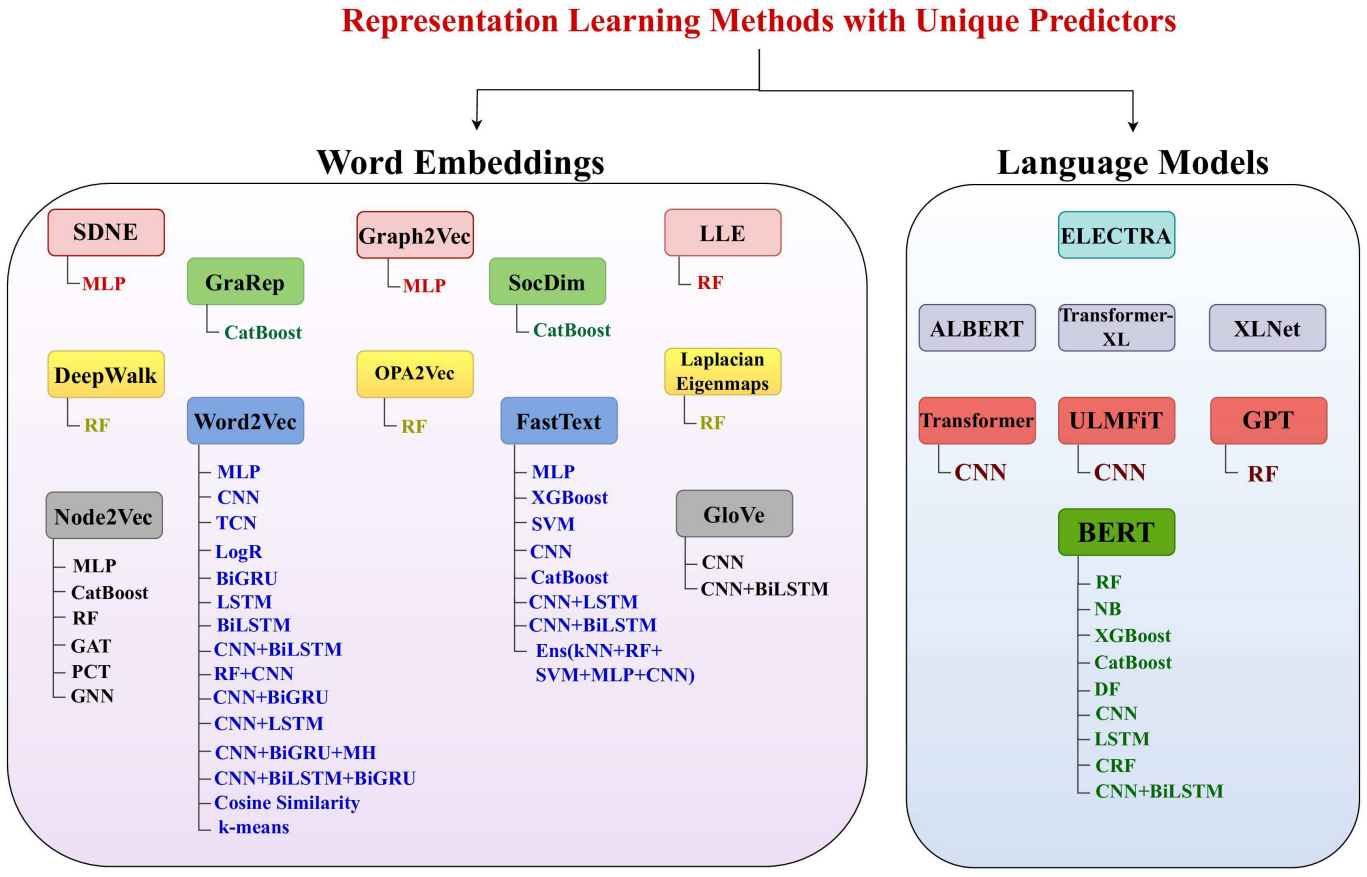
Utilization of 12 different word embedding approaches and 8 large language models in diverse DNA sequence analysis pipelines based on a variety of machine and deep learning predictors such that RF, random forest; DF, deep forest; SVM, support vector machine; LogR, logistic regression; NB, Naive Bayes; kNN, k-nearest neighbors; MLP, multilayer perceptron; CNN, convolutional neural network; GNN, graph neural network; GCN, graph convolutional network; TCN, temporal convolutional network; GAT, graph attention network; LSTM, long short-term memory; BiLSTM, bidirectional Llong short-term memory; BiGRU, bidirectional gated recurrent unit; PCT, predictive clustering tree; CRF, conditional random field; FGM, fast gradient method. All language models are used with self-classifiers and few language models like transformer, ULMFiT, GPT, and BERT are also used with separate standalone or hybrid algorithms.

In the domain of natural language processing (NLP), the introduction of word embedding techniques represented a significant advancement by enabling the development of more accurate machine and deep learning predictive models. These approaches assign statistical vectors to words by capturing contextual representations of words within extensive, unlabelled corpora (217, 218). The primary objective is to assign comparable vectors to semantically similar words and distinct vectors to dissimilar words (217, 218). Leveraging transfer learning strategies, these contextual word representations have empowered data-hungry deep learning models to achieve exceptional performance, even with limited training data. Following the success of word embeddings in various NLP tasks (217–220), researchers have adopted these approaches for genomic and proteomic sequence analysis tasks, which share similarities with NLP tasks. This section offers a comprehensive overview of 12 distinct word embedding approaches that are utilized in DNA sequence analysis predictive pipelines. Figure 5 visually illustrates the utilization of various word embedding methods in conjunction with different machine and deep learning algorithms.

These word embedding approaches leveraged for DNA sequence analysis tasks can be categorized into two types: (1) non-graph-based methods and (2) graph-based methods. Non-graph-based methods segregate DNA sequences into overlapping or non-overlapping k-mers. Specifically, overlapping k-mers are generated by sliding a fixed-size window over sequence with a smaller stride as compared to window size. For instance, for ACGTG sequence with a window size of 4 and a stride of 1, the k-mers generated are ACGT and CGTA. Alternatively, in non-overlapping k-mers generation, window and stride size must be equal in size. For same sequence used in the overlapping case, this non-overlapping approach generates only one k-mer, such as ACGT. The length of the k-mer is determined by the window size. Researchers often create pre-trained embeddings with different k-mer sizes and then select the size which yields best performance in downstream tasks. Once k-mers are generated, these k-mers sequences are passed to traditional word embedding models (Word2vec, FastText, GloVe) to generate representation.

A high level overview of Figure 5 indicates that various studies have explored the potential of the Word2Vec embedding method in combination with 13 different machine and deep learning algorithms as well as 2 statistical algorithms. The era of word embedding approaches begin in 2013 with introduction of Word2Vec (221). Word2vec has two different embeddings generation paradigm: (1) SkipGram and (2) Continuous Bag of Words (CBoW). SkipGram learns representations of k-mers by predicting surrounding k-mers for every k-mers of corpus. The number of surrounding k-mers is a hyper-parameter that can be adjusted according to available data. Contrarily, CBoW model learns k-mers representations by predicting single k-mer based on the context of its surrounding k-mers. Similar to SkipGram model, here context of surrounding k-mers is a hyper-parameter. Word2vec architecture is comprised of input layer, hidden layer, and an output layer. At input layer, a random d-dimensional vector is initialized for each k-mer, while the hidden layer extracts relationships between k-mers. These relationships are further passed to output layer, which predicts probabilities of output k-mers based on the context of input k-mers. The predicted probabilities are passed to loss function which computes loss value. To facilitate readers, Equation 10 embodies mathematical expressions for computing loss values of both variants.


(6) $$\{\begin{array}{c} E_{SkipGram}=-\frac{1}{N}\sum_{i=1}^{N}log(P(w_{i}|W_{s}))\\ E_{CBoW}=-\frac{1}{N}\sum_{i=1}^{N}\sum_{w_{j}\epsilon W_{s}}log(P(w_{j}|w_{i}))) \end{array}$$

In above expression, N refer to number of k-mers, *w*_*i*_ indicates target k-mers, *w*_*j*_ is one of k-mers within contextual window, and *W*_*s*_ refers to set of k-mers in contextual windows of k-mers *w*_*i*_. After computing loss, weights are updated during back propagation which eventually helps in generating similar vectors for similar k-mers and distinct vectors for dissimilar k-mers.

Pennington et al. (222) proposed another k-mers embedding approach named Global Vectors (GloVe) which generates k-mers vectors by capturing both global and local contextual information of k-mer within corpora. It can be seen in Figure 5, in the context of DNA sequence analysis, the potential of Glove k-mers embedding method is explored with two distinct deep learning methods. Primarily, this embedding generation method computes local and global contextual information by incorporating occurrence frequencies of k-mer pairs into an objective function shown in Equation 7.


(7) $$\begin{array}{l} J=\underset{\forall \left(w_{i},w_{j}\right)}{\sum }{\left(w_{i}w_{j}+b_{i}+b_{j}-log\left(f\left(C_{ij}\right)\right)\right)}^{2},\left(w_{i},w_{j}\right)\;\epsilon \;GeneratedPairs \end{array}$$

In above expression, *w*_*i*_ and *w*_*j*_ are k-mers within a pair, *b*_*i*_ and *b*_*j*_ are corresponding biases, and *f*(*C*_*ij*_) is a weighted function to normalize co-occurrence matrix values and eradicate biases and their impact of noise on k-mers embeddings.

Figure 5 shows that Word2Vec is the most commonly explored word embedding method, followed by FastText. Mikolov et al. (223) proposed FastText approach by extending the working paradigm of Word2Vec model. Primarily, this approach handles out-of-vocabulary (OOV) k-mers by discretizing k-mers into sub k-mers. After generating sub k-mers. it takes average of sub k-mers vectors to generate k-mers vectors and passed them to word2vec model. During back propagation, it updates vectors of both k-mers and sub k-mers. Through this strategy, vectors are generated for both k-mers and sub k-mers.

Furthermore, in NLP domain, with an aim to generate more comprehensive vectors of k-mers by capturing k-mers informative patterns from textual corpora, researchers have proposed different graph-based methods. These approaches include DeepWalk (224), Node2Vec (225), Graph2Vec (226), SDNE (227), SocDim (228), GraRep (229), Laplacian Eigenmaps (230), Locally Linear Embedding (231), and OPA2Vec (232). Figure 5 highlights that within the context of DNA sequence analysis, the potential of the 7 graph-based methods is less explored compared to the two foundational word embedding methods, Word2vec and FastText. In addition, among the graph-based methods, Node2Vec (225) has been investigated more extensively than DeepWalk (224), Graph2Vec (226), SDNE (227), SocDim (228), GraRep (229), Laplacian Eigenmaps (230), Locally Linear Embedding (231), and OPA2Vec (232). Similar to non-graph-based methods, graph-based methods segregate sequences into k-mers and generate k-mers pairs by sliding a 2 size window over k-mers sequences. By using k-mers paris, a graph is formed where nodes represent k-mers, and edges represent relationships between the k-mers. For example, to generate a graph from the DNA sequence ACTGCA with k = 3, first, overlapping k-mers (ACT, CTG, TGC, GCA) are generated. By sliding a window of size 2 over these k-mers sequence, k-mers pairs [(ACT, CTG), (CTG, TGC), and (TGC, GCA)] are created. These pairs form edges of graph, with k-mers serving as nodes. Perrozi et al. (224) proposed DeepWalk approach that utilizes graphical space to generate new sequences by capturing extensive relationships between k-mers. After generating new sequences, it makes use of Word2Vec model for generation of k-mers vectors. In contrast, Grover et al. (225) proposed Node2Vec approach that utilizes a distinct strategy for generation of new sequences. Primarily, Node2Vec employs second order random walk sampling strategy which reaps the benefits of breath first search (BFS) and depth first search (DFS) algorithms. This strategy computes probability of visiting next node depending on the previously visited nodes rather than just randomly selecting one of neighboring nodes. Naeayanan et al., (226) introduced another embedding generation approach namely Graph2Vec. It extracts root node, its sub-graph, and degree of intended sub-graph to generate a sorted list of nodes which is then passed to SkipGram with negative sampling (SGNS) model.

Matrix factorization embedding approaches extend graph-based embedding approaches by using adjacency matrix rather than generating new sequences directly from graph. Adjacency matrix encodes the relationships between nodes within the graph which is then decomposed using matrix factorization methods namely SVD and NMF. These approaches also decompose adjacency matrix of graph into lower-dimensional matrices which represents node embeddings. These embeddings extract nodes latent features and relationships between them. Mainly, matrix factorization methods aim to minimize reconstruction error between original adjacency matrix and reconstructed matrix from node embeddings. These methods include Laplacian Eigenmaps (230), Locally Linear Embedding (231), SDNE (227), SocDim (228), GraRep (229), and OPA2Vec (232). A closer view of Figure 5 indicates that 6 matrix factorization embedding approaches method are least explored as compared to foundational word embedding methods (Word2vec, FastText) and graph-based methods.

Laplacian Eigenmaps (230) approach derives degree matrix from adjacency matrix and computes graph Laplacian matrix by computing the difference between degree matrix and adjacency matrix. Next, it computes eigen values and constructs eigenvectors corresponding to smallest non-zero eigenvalues which results in generating lower-dimensional k-mer embeddings and preserving local k-mers relationships. Another matrix representation approach graph representations (GraRep) (229) make use of adjacency (*A*_*i, j*_) and degree (*D*_*i, j*_) matrices driven from nodes and edges of graph. Equation 8 depicts mathematical expression for computing proximity matrix from *A*_*i, j*_ and *D*_*i, j*_ matrices.


(8) $$\begin{array}{l} P_{i,j}=log\left(\frac{A_{i,j}}{D_{i,j}}\right)-log\left(\frac{1}{V}\right) \end{array}$$

where V represents total number of nodes in graphs. Calculated proximity matrix is further passed to singular value decomposition approach for generating k-mer embeddings. Moreover, this approach focuses on extracting similarities between nodes by using k-step information of neighbors where levels of neighbors can be represented through k-steps. Similar to GraRep approach, SocDim (228) generates k-mer representations by incorporating social dimensions, namely, attributes and network structures. Specifically in SocDim, adjacency and degree matrices are used to compute modularity matrix by using Equation 9.


(9) $$\begin{array}{l} M_{i,j}=A-\frac{1}{2m}\left(DD^{T}\right) \end{array}$$

where m represents edges, and D represents degree matrix. Similar to GraRep, modularity matrix is passed to SVD for k-mers embeddings generation.

Moreover, structural deep network embedding (SDNE) (227) leverages deep auto-encoders to generate k-mer embeddings by determining first and second order proximities to ensure connected k-mers have similar embeddings. SDNE model architecture is trained to optimize combined loss function that incorporates both proximities and finally generates low-dimensional representations by capturing non-linear relationships between nodes and encoding structural information into embeddings. Afterward, structural embedding aims to address limitations of k-mer embeddings approaches in capturing structural and semantic information of nodes and edges in heterogeneous networks. Among structural embedding approaches, Opa2Vec (232) makes use of individual entities containing structured knowledge or characterized classes axioms and unstructured information or metadata, i.e., textual annotations and passes them semantic reasoner tool (Elk/HermiT) for generating ontology sequence which is then passed to Word2Vec model for generating representations. Locally linear embedding (LLE) (231) method identifies neighboring k-mers for each k-mer in the sequence and determines weights by employing graph Laplacian concept which linearly reconstructs each k-mer from its neighbors. Afterward, it computes sum of edges between close k-mers by using heat-kernel method which ensures weights of connected k-mers as 1 and unconnected k-mers as 0, ultimately maintaining the reconstruction relationship. These weights extract both semantic and syntactic information and maintain the reconstruction relationship. By optimizing reconstruction error and computing eigenvectors, LLE generates embeddings for each k-mer in the sequence. These embeddings represent the k-mers in a reduced-dimensional space, where similar k-mers in context are closer together.

Specifically, for DNA sequence analysis tasks, word embeddings methods are being utilized to generate pre-trained embeddings in 2 different ways: In one way, sequences are segregated into k-mers and embeddings of k-mers are generated. In second way, embeddings are generated for whole DNA sequence. Moreover, most of the DNA sequence analysis predictors follow first way to generate embeddings (21, 39, 45, 47–49, 58, 59, 64–66, 76, 78, 79, 82, 92, 105, 106, 113, 121, 122, 136–138, 151, 153, 163, 165, 171, 173, 233–235), but second way is utilized by only few tasks including gene-disease association prediction (110), pseudogene function prediction (111), promoter identification (236), essential gene prediction (107, 108), gene network reconstruction (128), and gene expression prediction (103). In this section, we have defined methods from first way perspective. A comprehensive detail about second way is available in following articles (103, 107, 108, 110, 111, 128, 236).

In a nutshell, word embedding approaches have significantly propelled 44 distinct DNA sequence analysis tasks, enriching the research community with the development of robust and precise models. Notably, conventional word embedding techniques such as Word2Vec, GloVe, and FastText excel in capturing k-mers context and sub k-mers information effectively. In contrast, innovative techniques such as Graph2Vec, Node2Vec, DeepWalk, and GraRep harness graph-based methodologies to enhance embeddings based on connectivity and proximities. In addition, SocDim and OPA2Vec offer distinctive perspectives by integrating social and ontological elements, while SDNE combines local and global structural insights through deep autoencoders. Locally linear embedding (LLE) and Laplacian eigenmaps are dedicated to preserving local geometric properties. Ultimately, each approach makes a distinctive contribution to driving significant progress in DNA sequence analysis.

### 7.2 DNA sequence representation learning using language models

In the evolving landscape of natural language processing (NLP), the inception of the Transformer model has announced a new era of advancements, setting the precedent for subsequent developments in language models (237, 238). The Transformer and distinct language models, including BERT, GPT-3, and ELECTRA, have significantly contributed to pushing the boundaries of what machines can understand and generate in terms of human language (237, 238). The importance of these models lies not only in their ability to comprehend and produce text but also in their application across different domains including genomics and proteomics sequence analysis (239). These models have found multifarious applications in genomics and proteomics sequence analysis tasks by generating highly effective representations of biological sequences (239). To facilitate DNA sequence analysis researchers, here we briefly delve into the key features, advantages, and disadvantages of commonly used eight modern sophisticated language models, namely, Transformer (102), Transformer-XL (50), XLNet (156), ULMFIT, BERT (156), ALBERT (156), ELECTRA (156), and GPT-3 (240). Table 3 presents 8 distinct language models and their variants, categorized into 4 different groups based on their architectures. These architectures include trivial LSTM-based language model, encoder-decoder architecture, encoder-only architecture, and decoder-only architecture. Table 3 also provides information about language model architecture and outlines number of layers as well as count of encoders or decoders and their respective layers.

**Table 3:** Summary of 8 contemporary language models used in DNA sequence analysis.

**Architecture type**	**Language model, release year**	**Language model variants**	**Number of layers in encoders**	**Number of layers in decoders**
Trivial LSTM based language model	ULMFiT, (243), 2018	AWD-LSTM language model	1	_
Encoder-decoder	Transformer_XL (336), 2019	Transformer_XL Large (WikiText-103)	24	24
		24L Transformer_XL (text8)	24	24
		12L Transformer (enwik8)	12	12
		18L Transformer (enwik8)	18	18
		24L Transformer (enwik8)	24	24
		Transformer_XL base (Billion Word)	12	12
		Transformer_XL large (Billion Word)	24	24
Encoder-decoder	Transformer, (241), 2017	Base	6	6
		Big	6	6
	ELECTRA, (246), 2020	Small	12	_
		Base	12	_
		Large	24	_
	ALBERT, (245), 2020	Base	12	_
		Large	24	_
		xLarge	24	_
		xxLarge	12	_
	BERT, (244), 2019	Base	12	_
		Large	24	_
	XL-Net, (242), 2019	Base	12	_
		Large	24	_
Decoder-only	GPT, 2018	GPT-1 (337)	_	12
		GPT-2 small (338)	_	12
		GPT-2 medium (338)	_	24
		GPT-2 Large (338)	_	36
		GPT-3 (247)	_	96
		GPT-4 (339)	_	120

The Transformer model, introduced in 2017 by Vaswani et al., (241) marks a significant departure from previous models that relied on recurrent or convolutional neural networks for processing sequential data. This model utilizes a unique architecture that focuses on attention mechanisms which allows to handle long-range dependencies and understand the context and semantics of sequences more effectively (102, 241). Key innovations of the Transformer include positional encoding and self-attention mechanisms (102, 241). Positional encoding assigns a unique number to each individual k-mer or group of k-mers and helps in grasping k-mers order and sequence context. The self-attention mechanism allows the model to weigh the importance of each k-mer in relation to others, enhancing its ability to process and predict scientific language patterns (102, 241). The main advantage of the Transformer is its efficiency in training and inference due to parallel processing of sequences (102, 241). However, it requires substantial computational resources, which can be a limiting factor in resource-constrained environments. Despite this, its flexibility and scalability in handling diverse genomics tasks make it a preferred choice in many advanced AI applications (102).

Transformer-XL extends the Transformer architecture to address the limitation of fixed-length context by incorporating mechanisms that capture long-range dependencies more effectively (50). This model enhances the ability to maintain context over longer sequences than standard Transformer models, which significantly improves performance in various genomics and proteomics sequence analysis tasks (50). The core innovations of Transformer-XL include the introduction of a segment-level recurrence mechanism and a novel relative positional encoding (50). These features allow the model to reuse past information and thereby extend the context window across different segments. This design enables Transformer-XL to handle longer biological sequences efficiently and provides a substantial improvement over traditional models where each segment is processed in isolation (50). One of the main advantages of Transformer-XL is its capability to learn dependencies that are significantly longer than those captured by traditional models, leading to improvements in both short and long sequence analysis tasks (50). However, the model demands more memory due to its recurrence mechanism and larger context handling, which could be a limitation in resource-constrained environments.

XLNet extends the Transformer-XL model using an autoregressive method (242). This approach allows XLNet to learn bidirectional contexts by maximizing the expected likelihood over all permutations of the input sequence order which significantly enhances its scientific language understanding capabilities (242). Primary innovation of XLNet is its permutation language modeling (PLM), which enables the model to predict the likelihood of a sequence by considering all permutations of the k-mers within it (156, 242). This method allows XLNet to capture a comprehensive bidirectional context, unlike traditional autoregressive models only consider a single direction. In addition, XLNet incorporates a two-stream self-attention mechanism which enhances its ability to manage the context more effectively during the prediction process (156, 242). One of the main advantages of XLNet is its robustness in modeling bidirectional contexts, which significantly outperforms previous models such as BERT in numerous genomics sequence analysis tasks (156, 242). However, the complexity of its training process, which involves permutation of input sequences and a two-stream attention mechanism, may pose challenges in terms of computational resources and time (156, 242).

Universal Language Model Fine-tuning (ULMFiT) has revolutionized natural language processing by introducing effective transfer learning techniques for various NLP tasks. It is developed by Jeremy Howard and Sebastian Ruder in 2018 (243), and it typically leverages a pre-trained language model which is fine-tuned on specific DNA sequence analysis tasks having minimal sequences (243). ULMFiT utilizes Average Stochastic Gradient Descent - Long Short-Term Memory (AWD-LSTM) architecture to learn the distribution and contextual relationships of k-mers in DNA sequences (57). It employs self-supervised learning that predicts the next k-mer based on the previous known k-mers and enables the model to capture the semantics and discriminative potential of the sequences (57). The core innovation of ULMFiT lies in its ability to fine-tune pre-trained language models using techniques such as discriminative fine-tuning and the slanted triangular learning rates policy. Discriminative fine-tuning considers that different layers of neural network capture different kind of information; hence, it tunes every layer with distinct learning rates (243), whereas slanted triangular learning rate describes a unique learning rate scheduler that initially increases the learning rate and afterward drops it in a linear fashion (243). The short increase stage enables the model to quickly converge to a parameter space suitable for the task, while the extended decay period allows for more effective fine-tuning (243). By adjusting the learning rate for different layers, it prevents catastrophic forgetting and stabilizes the training process across various tasks (243). ULMFiT incorporates dropout techniques to regularize learnable parameters and prevent overfitting which ensures model's generalization ability (57). Another advantage of ULMFiT is its ability to achieve high performance with significantly less data compared to traditional models. However, the complexity of fine-tuning and the need for careful calibration of learning rates can be challenging, requiring a nuanced understanding of model behavior across different layers (57).

Bidirectional Encoder Representations from Transformers (BERT) is developed by Google in 2018 (244). It is pretrained on a large corpus of text data, such as Wikipedia and books (244). It has revolutionized NLP tasks by employing a transformer-based architecture that enables the model to consider the context of k-mers from both directions simultaneously, rather than a single direction at a time (244). BERT is distinctive for its deep bidirectional nature, achieved through the application of the transformer model, specifically using mechanisms such as Masked Language Modeling (MLM) and Next Sentence Prediction (NSP) (244). This approach allows BERT to understand the context of a k-mer based on all other k-mers in a sequence, rather than just those preceding it. Specifically, it learns to capture the semantics and contextual information of the input text exceptionally well through self-supervised learning tasks such as MLP and NSP (244). In the case of DNA sequence analysis, BERT is used to transform DNA sequences into statistical feature space and then fine-tuned on specific downstream tasks, such as enhancer identification and strength prediction (156). BERT captures the semantics of DNA sequences by dynamically learning their representations through a multihead self-attention mechanism. BERT leverages transfer learning by pre-training on a large corpus and then fine-tuning on specific DNA sequence analysis task, allowing it to adapt to different applications (156). BERT uses MLM and NSP tasks during pre-training to learn the contextual relationships between k-mers in DNA sequences (156).

The primary advantages of BERT include its high accuracy and efficiency across various DNA sequence analysis tasks, due to its robust handling of context and bidirectional training (156). BERT captures both discriminative and semantical relationships of k-mers, making it effective in characterizing DNA sequences (156). BERT-based models have shown improved performance compared to traditional approaches in different DNA sequence analysis tasks such as enhancer identification and strength prediction (156). In addition, BERT can be adapted to specific application scenarios by pre-training on domain-specific custom corpora (156). BERT is a large model that requires significant computational resources for training and inference on extensive datasets. BERT performs best when trained on large and diverse datasets, which may not always be available for specific DNA sequence analysis tasks. In addition, while BERT provides state-of-the-art results in many scenarios, it requires fine-tuning for specific tasks, which can be resource-intensive. BERT performance can degrade with longer texts and the complex architecture of BERT makes it challenging to interpret the learned representations and understand the underlying biological mechanisms (156).

ALBERT, introduced by Google researchers, is a streamlined version of BERT designed to provide state-of-the-art results in NLP with significantly fewer parameters (245). This model enhances the efficiency and scalability of BERT by incorporating innovative techniques such as factorized embedding parameterization, cross-layer parameter sharing, and sentence order prediction (156, 245). Factorized embedding parameterization technique reduces the size of the embedding matrix by separating the vocabulary and hidden layer sizes, which decreases the number of parameters significantly (156, 245). In cross-layer parameter sharing, parameters are shared across all layers of the model, reducing the total parameter count and improving training efficiency (156, 245). It replaces the next sequence prediction with sequence order prediction to enhance the model's ability to understand sequence coherence without requiring task prediction, making it more effective for downstream tasks (156, 245). The primary advantage of ALBERT is its reduced parameter size, which allows for faster training times and less memory usage compared to BERT, without a significant loss in performance. However, the extensive parameter sharing might lead to a slight decrease in model flexibility, potentially affecting task-specific fine-tuning (156, 245). Efficiently Learning an Encoder that Classifies Token Replacements Accurately (ELECTRA) (156, 246) has introduced a novel pre-training method for language models. ELECTRA operates on a replaced token detection (RTD) mechanism, where it differs from traditional masked language models such as BERT (156, 246). Instead of masking k-mers, ELECTRA corrupts the input by replacing tokens or k-mers with outputs from a generator model, challenging the discriminator to identify changes (156, 246). This approach allows the model to learn from the entire input sequence, enhancing training efficiency. The primary advantage of ELECTRA lies in its efficiency, requiring less computational power and time to reach or exceed the benchmarks set by larger models (156, 246). However, the complexity of its dual-model architecture, involving both a generator and a discriminator, might pose challenges in training stability and hyperparameter tuning.^8^

GPT-3 is one of the most advanced AI language models developed by OpenAI (247). It is recognized for its ability to generate text that closely mimics human writing, making it a pivotal development in natural language processing. GPT-3 builds upon the transformer architecture, which utilizes self-attention mechanisms to process input data (247). Unlike GPT-2, which had 1.5 billion parameters, GPT-3 boasts a staggering 175 billion parameters. This exponential increase in parameters enhances its ability to generate coherent and contextually relevant text (247). GPT-3 differs from models such as BERT and XLNet by maintaining an autoregressive nature. From scientific perspective, this implies that it predicts the next k-mer in a sequence based on the previous k-mers, while BERT uses bidirectional context (240, 247). One of the innovative aspects of GPT-3 is its use of alternating dense and locally banded sparse attention patterns. Dense attention considers all input k-mers simultaneously, while sparse attention focuses on a subset of k-mers, making the model more efficient and scalable. This combination enables GPT-3 to handle long-range dependencies and maintain computational efficiency (240, 247). One of GPT-3's standout capabilities is its performance in few-shot settings. Unlike fine-tuned models that require large amounts of task-specific data, GPT-3 can perform well on new tasks with minimal sequences. This flexibility is a significant advantage over models such as BERT, which typically require extensive fine-tuning for each specific task. GPT-3 demonstrates strong performance across various tasks, often matching or exceeding that of fine-tuned models. This capability makes it a versatile tool for a wide range of applications (240, 247).

For instance, in context of cell biology, scientific researchers have used GPT-3 to learn gene and cell embeddings effectively (240). Scientific researchers have utilized the text summaries of genes from the NCBI database, which contain curated information about gene functionalities and properties. The gene text summaries are passed through the GPT-3 language model, which generates gene embeddings that capture the underlying biology described in the gene summaries (240). The gene embeddings are averaged, weighted by the expression levels of each gene in the cell. These averaged embeddings are then normalized to a unit l2 norm to generate single-cell embeddings (240). In another strategy, each cell is represented by a natural language sentence constructed based on the ranked gene expressions. The gene names are ordered by descending normalized expression levels, and this sentence representation is passed through the GPT-3 model to obtain the cell embeddings (240). Extrinsic performance analysis of GPT-3 embeddings on tasks such as classifying gene properties or cell types has shown supreme effectiveness (240). While GPT-3's capabilities are groundbreaking, it faces challenges such as potential biases in training data and high computational demands. Moreover, its “black box” nature makes it difficult to discern how decisions are made, posing ethical and operational concerns. GPT-3's massive size requires significant computational resources for both training and inference. This makes it less accessible for smaller organizations or researchers without high-end hardware.

### 7.3 Machine and deep learning predictors

Machine and deep learning algorithms need statistical vectors to extract useful patterns for specific sequence analysis task. A comprehensive literature review of 127 studies reveals that 12 word embedding and 8 large language models have been used to generate statistical vectors of raw sequences to feed 28 different algorithm available within predictive pipelines of 44 DNA sequence analysis tasks. Based on working paradigms, these algorithms are categorized into 3 different categories, namely, statistical algorithms, machine learning algorithms, and deep learning algorithms. From 28 algorithms, 3 algorithms, namely, conditional random fields (CRF) (248), k-means clustering algorithm (21), and cosine similarity algorithm (173), belong to statistical algorithms. Machine learning algorithms involves 8 algorithms, namely, support vector machine (SVM) (352), Naive Bayes (NB) (95), multilayer perceptron (MLP) (77), predictive clustering tree (PCT) (128), random forest (RF) (103), deep forest (DF) (61), XGBoost (352), and CatBoost (143). Furthermore, deep learning algorithms include convolutional neural network (CNN) (91), graph neural network (GNN) (104), temporal convolutional network (TCN) (235), graph convolutional network (GCN) (55), graph attention network (GAT) (108), long short-term memory (LSTM) (97), bidirectional long short-term memory (BiLSTM) (58), and bidirectional gated recurrent unit (BiGRU) (82). Similarly, 8 algorithms, namely, ELECTRA (89), ALBERT (249), Transformer-XL (50), XL-Net (156), Transformer (250), ULMFit (146), GPT-3 (114), and BERT (251), belong to language modeling algorithms, five algorithms, namely, LSTM + CNN, CNN + BiLSTM, CNN + BiLSTM + BiGRU, RF + CNN, and CNN + BiGRU, belong to hybrid algorithms, whereas 1 meta-predictor reaps benefits of both machine and deep learning algorithms, namely, KNN, RF, SVM, MLP, and CNN.

Statistical algorithms provide a framework for understanding DNA sequence distribution and characteristics. They offer valuable advantages in terms of interpretability by facilitating researchers to assess statistical significance of genomic features. Among three statistical algorithms, Conditional Random Fields (CRF) (248) calculate the conditional probability of class labels of sequences by using neighboring k-mers. By capturing dependencies between adjacent labels, CRF allows for more accurate predictions of sequence features by taking into account both local sequence context and broader genomic patterns. K-means clustering algorithm (21) groups sequences into k distinct clusters based on similarity. It starts by initializing k centroids and assigns each sequence to the nearest cluster by calculating Euclidean distance between sequence and centroid. These centroids are updated iteratively by averaging the sequences in each cluster until they stabilize. Cosine similarity can be advantageous in DNA sequence analysis for tasks such as similarity comparison and clustering, where measuring the similarity between sequences is essential (252). Cosine similarity can handle high-dimensional data efficiently and is suitable for tasks requiring similarity-based analysis (252). These models also have limitations, including potential difficulties in managing complex and high-dimensional data. In addition, they rely on strong assumptions about underlying data distribution, which may not always align with real-world DNA sequence analysis scenarios. Despite these challenges, statistical models remain indispensable tools in DNA sequence analysis and provide valuable insights.

From 8 different machine learning algorithms, support vector machine (SVM) operates by finding the optimal hyperplane that best separates data points into different classes. SVMs are known for their ability to handle high-dimensional data and work well in cases where the data are not linearly separable, as they can use kernel functions to transform the data into higher dimensions where separation is possible (62). However, SVMs can have limitations in terms of training time, especially with large datasets, as they need to solve a complex optimization problem to find the best hyperplane that separates the classes. Naive Bayes (NB) is a probabilistic algorithm based on Bayes' theorem with the assumption of independence between features. NB is efficient, simple to implement, and works well with high-dimensional data, making it suitable for tasks where feature independence assumptions hold (253). However, it may not always hold true in practice, especially in complex biological datasets where features are correlated.

In addition to SVM, tree-based algorithms are fundamentally built upon decision tree algorithm. Decision tree algorithm uses independent variables to construct a tree-like structure, where data are split at decision nodes into branches connected to leaf nodes to make predictions. This foundational algorithm is extended into more advanced algorithms, namely, Random Forest (RF), Deep Forest (DF), XgBoost, CatBoost, and Predictive Clustering Tree (PCT) (128). All of these advanced algorithms enhance basic decision tree by incorporating techniques such as ensembling, and boosting for improved accuracy and generalization. Random Forest (RF) algorithm is an ensemble learning method that constructs a multitude of decision trees during training and outputs the mode of the classes as the prediction. RF is known for its robustness to overfitting, feature importance estimation, and ability to handle high-dimensional data with ease (254). However, RF may not perform as well when dealing with imbalanced datasets or when there are many irrelevant features present in the data. Deep forest (DF) algorithm is another ensemble learning method that utilizes a cascade structure of multiple random forests to make predictions. DF can be advantageous in DNA sequence analysis for tasks such as clustering and species classification based on DNA barcodes (255). DFs are capable of learning hierarchical representations of data and can capture complex patterns in high-dimensional spaces effectively (255). Nonetheless, the main drawback of DF lies in its computational complexity and the need for substantial computational resources, which can limit its practicality in large-scale DNA sequence analysis projects. XGBoost combines multiple weak learners to create a strong predictive model. XGBoost can handle large datasets with high dimensionality and is known for its efficiency in boosting the performance of weak learners (256). However, XgBoost may require fine-tuning of hyperparameters to achieve optimal performance, and it could be sensitive to noisy data. CatBoost is another ensemble learning method designed to handle categorical features efficiently. CatBoost can automatically handle categorical features and is known for its robustness to overfitting and efficiency in training models with categorical data (256). Nevertheless, CatBoost's training time might be longer compared to other algorithms, especially when dealing with large genetic datasets.

Predictive clustering tree (PCT) (128) is a versatile predictor that integrates elements of both clustering and supervised learning. Unlike traditional decision trees, random forests, or support vector machines, PCTs are designed to handle hierarchical multi-label classification tasks, making them particularly effective for complex, high-dimensional data (128). PCTs operate by viewing a decision tree as a hierarchy of clusters. The root node represents a single cluster containing all training examples, which is recursively partitioned into smaller clusters as one moves down the tree. This approach allows PCTs to simultaneously perform clustering and classification, leveraging the hierarchical structure to predict multiple labels for each instance (128). One of the key strengths of PCTs is their ability to manage complex data with multiple interrelated labels. They can identify relevant features across different levels of the hierarchy, providing interpretable results that are valuable for domain experts. In addition, PCTs are capable of handling large datasets efficiently, making them suitable for various real-world applications (128). Despite their strengths, they can be computationally intensive, especially for large and deep hierarchies, and may require careful parameter tuning to avoid overfitting. In addition, while PCTs offer interpretability, the complexity of the hierarchical structure can sometimes make the results harder to interpret compared to simpler models (128). Apart from this, researchers have also designed customized meta-predictors which utilize the powers of five or more than five distinct algorithms, namely, kNN, RF, SVM, MLP, and CNN (105).

Multilayer perceptron (MLP) is composed of multiple layers of nodes that can learn complex patterns in data. MLPs are powerful algorithms for feature extraction and predictive modeling in DNA sequence analysis, capable of capturing intricate relationships in the data (252). MLPs excel in tasks requiring non-linear decision boundaries and can handle large amounts of data effectively. However, training MLPs can be computationally expensive, especially with large datasets, and they are prone to overfitting if not properly regularized.

Among all categories, deep learning algorithms are most extensively used for efficient DNA sequence analysis. A total of eight deep learning algorithms are most commonly used by scientific community for DNA sequence analysis. Convolutional neural network (CNN) is a deep learning algorithm designed to process structured grid-like data, such as images. In DNA sequence analysis, CNNs can be applied to DNA sequence analysis tasks to capture spatial dependencies in data. They are effective for tasks that require feature hierarchies and translation invariance (257). However, CNNs may struggle with capturing long-range dependencies in sequences, which can be crucial in DNA analysis where distant k-mers may interact. Graph neural network (GNN) is a type of neural network designed to operate on graph-structured data. GNNs are suitable for tasks involving relational data, such as molecular structures, making them applicable to DNA sequence analysis for tasks such as clustering (258). GNNs can effectively capture dependencies between nodes in a graph and are capable of learning representations that incorporate both local and global information (258). However, GNNs may encounter challenges in efficiently scaling to large graphs, and interpreting the learned representations in GNNs can be complex, limiting their interpretability. Temporal convolutional network (TCN) is a type of neural network designed to process sequential data efficiently. TCNs are suitable for tasks involving temporal dependencies, making them applicable to DNA sequence analysis for tasks like predicting DNA binding sites for transcription factors. TCNs can capture long-range dependencies in sequential data and are known for their parallel processing capabilities, enabling faster training times (259). However, TCNs may struggle with modeling complex temporal dynamics compared to recurrent models such as LSTMs. Graph convolutional network (GCN) is a type of neural network designed to operate on graph-structured data. GCNs can leverage graph structures to learn representations of nodes and edges, enabling tasks such as node classification and link prediction in DNA sequences (256). However, GCNs may require meticulous graph construction and preprocessing, and they can be computationally intensive, especially for large graphs, which can hinder their scalability.

Graph attention network (GAT) is a type of neural network that incorporates attention mechanisms to learn the importance of neighboring nodes in a graph. GATs are suitable for tasks involving relational data, as they can adaptively weigh the contributions of neighboring nodes, enabling more flexible and accurate learning on graph-structured data (260). However, GATs may be sensitive to noisy or sparse graphs, and designing optimal attention mechanisms can be challenging, impacting their performance in certain scenarios. Long short-term memory (LSTM) is a type of recurrent neural network designed to capture long-term dependencies in sequential data. LSTMs are effective in DNA sequence analysis for tasks such as hypersensitive DNA sequence classification (261). LSTMs can retain information over long sequences and are suitable for tasks requiring memory of past events, making them ideal for tasks such as classification of DNA sequences (261). However, LSTMs may encounter vanishing or exploding gradient problems during training, which can affect their ability to capture long-term dependencies accurately. Bidirectional long short-term memory (BiLSTM) is an extension of LSTM that processes sequences in both forward and backward directions. BiLSTMs are advantageous in DNA sequence analysis for tasks where contextual information from both past and future is essential (262). BiLSTMs can capture dependencies in both directions and are effective in tasks requiring bidirectional context understanding (262). However, BiLSTMs may be computationally intensive due to processing sequences in two directions, which can impact their training and inference speed. Bidirectional gated recurrent unit (BiGRU) is another type of recurrent neural network that combines the advantages of bidirectionality and gating mechanisms. BiGRUs can capture bidirectional dependencies efficiently and are known for their simpler architecture compared to LSTMs, making them computationally more efficient (256). However, BiGRUs may struggle with capturing very long-term dependencies compared to LSTMs, which can limit their effectiveness in tasks requiring extensive memory retention.

For different DNA sequence analysis tasks, eight contemporary language models, namely, ELECTRA, ALBERT, Transformer-XL, XLnet, Transformer, ULMFit, GPT-3, and BERT have been used in two different settings. In first setting, the addition of classification layers to these language models adapts the general-purpose language models to specific classification tasks by learning to map the rich contextual embeddings to the desired output classes. In second setting, rich contextual embeddings of these 8 language models are passed to standalone machine learning algorithms, deep learning algorithms, and ensemble or hybrid algorithms for accurate classification of DNA sequences.

A total of five hybrid algorithms combine different types of models to leverage the strengths of each component. LSTM + CNN, CNN + BiLSTM, CNN + BiLSTM + BiGRU, RF + CNN (76), and CNN + BiGRU are some of the examples of hybrid algorithms that integrate deep learning and traditional machine learning techniques to enhance predictive performance (263) for different DNA sequence analysis tasks. These hybrid models aim to capitalize on the complementary advantages of different algorithms to achieve superior results in various tasks.

## 8 Uncovering evaluation measures for DNA sequence analysis predictive pipelines

AI-driven DNA sequence analysis predictive pipelines are evaluated using two different experimental settings: (1) k-fold cross-validation (48, 78) and (2) Train-test split (108, 110). In k-fold cross-validation, dataset is splitted into k folds, where *k*−1 folds are used for training and one fold is used for testing. In next iterations, from k-folds, another fold is reserved for testing whereas remaining *k*−1 folds are used for training. In this way, pipelines are trained and tested k times on whole data. This method offers more precise assessment of model generalization capability. Specifically for deep learning models (236), an additional set, known as validation set, is created from training set which typically uses 10% of training data. This validation set is used to optimize the model's hyperparameters. In train-test split experimental setting, data are divided into two distinct sets: (a) train set and (b) test set. Train set comprises majority of data (usually 70%–80%), while test set contains remaining 20%–30%. Similar to k-fold cross-validation, validation set is also created from train set for deep learning models.

Among 127 DNA sequence analysis studies, 67 studies have utilized 5-fold cross-validation-based experimental setting. Thirty five studies have used 10-fold cross-validation-based setting and 17 studies have used train test split-based setting. Eight studies have used both k-fold cross validation and train test split-based setting. Performance and effectiveness of trained predictive pipelines highly depends on ability to handles new and unseen data. To assess effectiveness and performance of predictive pipelines from different perspectives, various evaluation measures have been proposed. Based on task type, these measures are categorized into four classes: binary (92, 153)/multi-class classification (27, 173), multi-label classification (111, 112), regression (102, 103), and clustering (21). Following subsections summarize details of all four types of evaluation measures.

### 8.1 Binary or multi-class classification evaluation criteria

**Figure 6:**
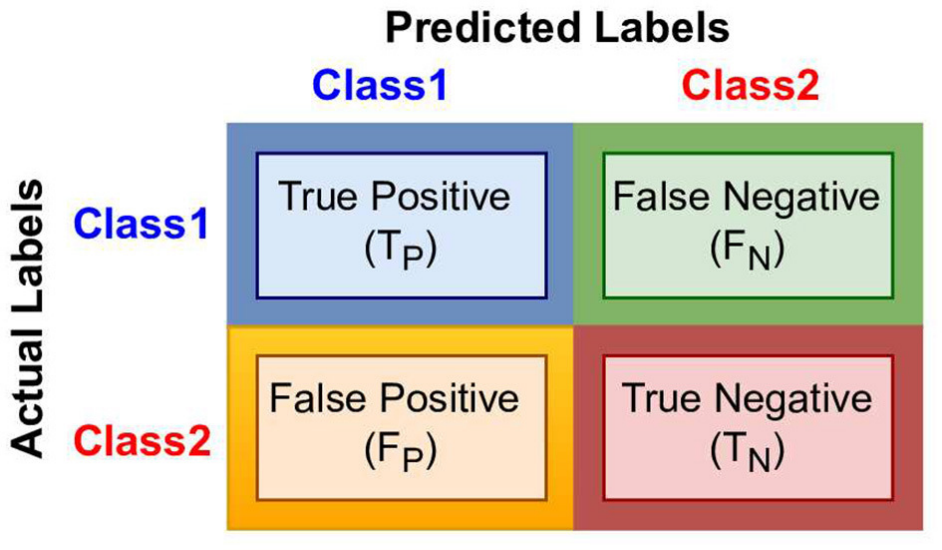
Overview of confusion matrix.

Most commonly used evaluation measures in this category are accuracy (152, 153, 264), precision (137, 152), recall (152, 153), specificity (137, 153), F1 Score (137, 152), and MCC (137, 153). These measures are typically calculated using confusion matrix, which consists of four entities: true positives (*T*_*P*_), false positives (*F*_*P*_), true negatives (*T*_*N*_), and false negatives (*F*_*N*_) (265). Figure 6 makes use of aforementioned four entities to compute distinct evaluation measures.

It can be seen in Figure 6 that *T*_*P*_ and *T*_*N*_ indicate correct positive and negative predictions, while *F*_*P*_ and *F*_*N*_ signify incorrect positive and negative predictions. Equation 10 embodies mathematical expressions to compute aforementioned measures.


(10) $$f{\left(x\right)}_{balanced}=\{\begin{array}{c} Accuracy\;(Acc)=\frac{T_{P}+T_{N}}{T_{P}+F_{P}+T_{N}+F_{N}}\\ Precision\;(PR)=\frac{T_{P}}{T_{P}+F_{P}}\\ Recall\;(REC)=\frac{T_{P}}{T_{P}+F_{N}}\\ F1-Score=\frac{2*Pr*R}{Pr+R}\\ Specificity\;(Sp)=\frac{T_{N}}{T_{N}+F_{P}}\\ MCC=\frac{\left(T_{P}\times T_{N})-(F_{P}\times F_{N}\right)}{\sqrt{\left(T_{P}+F_{P})(T_{P}+F_{N})(T_{N}+F_{P})(T_{N}+F_{N}\right)}} \end{array}$$

An in-depth assessment of existing DNA sequence analysis predictive pipelines reveals that most widely used evaluation measures for balanced datasets are F1-score, precision, accuracy, recall, specificity, and Matthews correlation coefficient (MCC). However, for imbalanced datasets, micro, macro, and weighted versions of these measures are used. To address class imbalance issue, weighted precision (266) considers both precision and relative weight of each class. Precision of a class is ratio of true positives to total number of positives for that class, while relative weight is proportion of samples of that class relative to total number of samples. Similarly, weighted recall (266) and weighted F1-score (27) are calculated by determining weights, recall, and F1-score for each class. Macro precision (146) calculates precision for each class independently and then averages these values. Macro recall (146) and macro F1-score (146) average recall and F1-score across all classes by considering each class equally regardless of size. In contrast, micro precision (146) calculates precision globally by considering all true positives and false positives across all classes together. Micro recall (146) and micro F1-score (146) aggregate *T*_*P*_, *F*_*P*_, and *F*_*N*_ across all classes and provides a fair and balanced evaluation of predictor performance. Equation 11 provides mathematical expressions for computing these measures.


(11) $$f{\left(x\right)}_{imbalanced}=\{\begin{array}{c} Micro\;Precision=\frac{\sum_{i=1}^{n}T_{P}^{\;i}}{\sum_{i=1}^{n}\left(T_{P}^{\;i}+F_{P}^{\;i}\right)}\\ Micro\;Recall=\frac{\sum_{i=1}^{n}T_{P}^{\;i}}{\sum_{i=1}^{n}\left(T_{P}^{\;i}+F_{N}^{\;i}\right)}\\ Micro\;F1-Score=\frac{\sum_{i=1}^{n}2.T_{P}^{\;i}}{\sum_{i=1}^{n}\left(2.T_{P}^{\;i}+F_{P}^{\;i}+F_{N}^{\;i}\right)}\\ Macro\;Precision=\frac{1}{n}\sum_{i=1}^{n}Pr^{i}\\ Macro\;Recall=\frac{1}{n}\sum_{i=1}^{n}R^{i}\\ Macro\;F1-Score=\frac{1}{n}\sum_{i=1}^{n}F1-Score^{i}\\ Weighted\;Precision=\frac{\sum_{i=1}nPr^{i}.w^{i}}{\sum_{i=1}^{n}w^{i}}\\ Weighted\;Recall=\frac{\sum_{i=1}nR^{i}.w^{i}}{\sum_{i=1}^{n}w^{i}}\\ Weighted\;F1-Score=\frac{\sum_{i=1}^{n}F1-score^{i}.w^{i}}{\sum_{i=1}^{n}w^{i}} \end{array}$$

where ${T_{P}}^{i}$, ${F_{P}}^{i}$, and ${F_{N}}^{i}$ refer to true positives, false positive, and false negatives in class i, respectively. *Pr*^*i*^, *R*^*i*^, and *F*1−*score*^*i*^ signify precision, recall, and F1-score of class *i*. *w*_*i*_ indicates relative weight of class i, and *i* refers to *i*^*th*^ class among *n* classes.

### 8.2 Multi-label classification evaluation measures

Performance evaluation of multi-label classification predictive pipelines is more challenging compared to binary and multi-class classification predictive pipelines (267). In binary or multi-class classification, each sample is assigned to only one class at a time, so predicted class label will be either true or false. Contrarily, in multi-label classification, a sample belongs to two or more labels simultaneously and predictive pipelines predicts multiple labels (267). Among predicted labels, some labels can be correct, some labels can be incorrect, or all predicted labels can be correct or incorrect. This partial correctness introduces complexity. To address this problem, researchers have developed various evaluation measures, namely, accuracy (268), precision (268), recall (268), and hamming loss (269).

Equation 12 illustrates the mathematical expressions for these evaluation measures.


(12) $$f{\left(x\right)}_{multi-label}=\{\begin{array}{c} Accuracy\;(Acc)=\frac{1}{N}\sum_{i=1}^{N}\left|\frac{A^{i}\wedge P^{i}}{A^{i}\vee P^{i}}\right|\\ Recall\;(REC)=\frac{1}{N}\sum_{i=1}^{N}\frac{\left|A^{i}\wedge P^{i}\right|}{\left|A^{i}\right|}\\ Precision\;(PR)=\frac{1}{N}\sum_{i=1}^{N}\frac{\left|A^{i}\wedge P^{i}\right|}{\left|P^{i}\right|}\\ F1-Score=\frac{1}{N}\sum_{i=1}^{N}\frac{2*\left|Pre(n^{i})*Rec(n^{i})\right|}{\left|Pre(n^{i})+Rec(n^{i})\right|}\\ HammingLoss=\frac{1}{Nl}\sum_{i=1}^{N}\sum_{j=1}^{l}\left[\left|(A_{j}^{\;i}\neq P_{j}^{\;i})\right|\right] \end{array}$$

In these equations, *N* represents total number of samples, *n*^*i*^ denotes *i*^*th*^ sample out of *N* samples, *A*^*i*^ is actual class label, and *P*^*i*^ is the predicted label for *n*^*i*^ sample. *l* represents sample length, *j* denotes class index, ∨ signifies logical OR operator, and ∧ represents logical AND operator. Similar to evaluation measures in binary or multi-class classification and includes micro precision, micro recall, micro F1-score, macro precision, macro recall, macro F1-score, weighted precision, weighted recall, and weighted F1-score. A rigorous analysis of existing literature on multi-label classification tasks in DNA sequence analysis reveals that most widely used evaluation metrics are accuracy, precision, recall, F1-score, MCC, sensitivity, and specificity.

### 8.3 Regression evaluation criteria

Regression tasks differ fundamentally from classification tasks where model predicts continuous numerical values rather than discrete class labels. Regression task-related predictive pipelines are evaluated using distinct evaluation measures including mean squared error (MSE) (270), mean absolute error (MAE) (271), mean bias error (MBE) (272), mean absolute percentage error (MAPE) (273), root mean square error (RMSE) (271), *R*^2^ (274), relative mean absolute error (rMAE) (275), relative mean square error (rMSE) (275), relative root mean square error (rRMSE) (275), and relative mean bias error (rMBE) (275).

MAE assesses predictor performance by measuring absolute difference between predicted and actual values (271). MSE quantifies deviation by averaging squared differences between actual and predicted values (270). Similarly, RMSE calculates standard deviation of prediction errors and demonstrates how tightly data points cluster around regression line (271). MBE assesses predictor performance in terms of under and overfitting by enumerating average difference between predicted and actual value (272). MAPE calculates percentage variation between predicted and actual values (273). Smaller the values of MAE, MBE, MSE, and MAPE, better will be predictor performance. Higher value of *R*^2^ score signifies promising predictor performance as it measures proportion of variance in predicted dependent variable explained by independent variable to determine strength of relationship.

MAE, MSE, RMSE, and MAPE measures compute average error value for N number of data points. Relative performance evaluation can improve quality of performance evaluation by reducing the noise from data. For relative performance evaluation, the percentage error of each metric is computed relative to the average of actual values (275). It facilitates in controlling factors that influence predictor performance by relatively calculating ratio of particular error with average of actual values (275). Since data continuously vary and produce varying predicted values at different time intervals, an overall percentage error is computed to obtain relative error of all data points. Equation 13 embodies mathematical expressions for aforementioned evaluation metrics.


(13) $$f{\left(x\right)}_{regression}=\{\begin{array}{c} MAE=\frac{1}{M}\sum_{i=1}^{M}\left|P^{i}-A^{i}\right|\\ MSE=\frac{1}{M}\sum_{i=1}^{M}{\left(A^{i}-P^{i}\right)}^{2}\\ RMSE=\sqrt{\frac{1}{M}\sum_{i=1}^{M}{\left(A^{i}-P^{i}\right)}^{2}}\\ MBE=\frac{1}{M}\sum_{i=1}^{M}\left(P^{i}-A^{i}\right)\\ MAPE=\frac{1}{m}\sum_{i=1}^{M}\left|\frac{P^{i}-A^{i}}{A^{i}}\right|\times 100\\ R^{2}Score=1-\frac{\sum_{i=1}^{M}{\left(P-A\right)}^{2}}{\sum_{i=1}^{M}{\left(A-avg\left(A\right)\right)}^{2}}\\ rMAE=\frac{MAE}{\bar{A}}\times 100\\ rMSE=\frac{MSE}{\bar{A}}\times 100\\ rMBE=\frac{MBE}{\bar{A}}\times 100\\ rRMSE=\frac{RMSE}{\bar{A}}\times 100 \end{array}$$

In Equation 13, *M* denotes total number of samples, *A*^*i*^ represents actual value, and *P*^*i*^ is predicted value where i denotes the sample number and Ā is the average of total actual values.

### 8.4 Clustering evaluation measures

In contrast to first three categories explained, clustering tasks aim to group similar samples based on their features without predefined class labels. In this task, prime objective is to use clustering algorithms and identify inherent patterns or structures within data. In these tasks, clusters of data samples with similar features are created, and predictors assign new data points to appropriate clusters (276). A higher similarity to a cluster indicates that data sample belongs to that cluster (276). To assess clustering predictive pipeline performance, researchers have introduced various evaluation measures including accuracy (277), normalized mutual information (NMI) (277), silhouette score (SS) (278), dunn index (DI) (279), and Davies-Bouldin index (DBI) (280).

Accuracy (277) is the proportion of correctly predicted samples to total number of samples. NMI (277) quantifies quality of predictor by measuring mutual information between predicted clusters and actual clusters. Mutual information refers to computed joint probability between predicted clusters and actual clusters. Silhouette score (278) measures how similar data samples are within a cluster compared to other clusters. BDI (280) evaluates average similarity ratio of each cluster with its most similar cluster. DI (279) computes ratio of minimum inter-cluster distance to maximum intra-cluster distance. Equation 14 embodies mathematical expressions for these evaluations measures.


(14) $$f{\left(x\right)}_{clustering}=\{\begin{array}{c} Accuracy=\underset{max}{m}\frac{\sum_{i=1}^{n}1\left\{y_{i}=m(c_{i})\right\}}{n}\\ NMI=\frac{I(y_{i},c_{i})}{\frac{1}{2}\left[H(y_{i})+H(c_{i})\right]}\\ SS=\frac{min\left\{d(y_{i})\right\}-a(y_{i})}{max\left\{min\left\{d(y_{i})\right\},a(y_{i})\right\}}\\ DBI=\frac{1}{n}\sum_{i=1}^{n}\underset{j\neq i}{max}(\frac{\bar{S_{i}}+\bar{S_{j}}}{d(c_{i},c_{j})})\\ DI=\frac{min_{1\leq i<j\leq n}d(c_{i},c_{j})}{max_{1\leq k\leq n}d^{\prime }(c)} \end{array}$$

In Equation 14, *y*_*i*_ refers to predicted cluster, *c*_*i*_ and *c*_*j*_ indicate *i*^*th*^ and *j*^*th*^ clusters among *n* clusters. Moreover, *I*(*y*_*i*_, *c*_*i*_) signifies mutual information, *H*(*y*_*i*_) and *H*(*c*_*i*_) show entropy of predicted and actual clusters. *d*(*y*_*i*_) is the average distance from *y*_*i*_ to all points in other clusters, and *a*(*y*_*i*_) is the average distance of *y*_*i*_ to all points in that clusters. *d*(*c*_*i*_, *c*_*j*_) represents inter-cluster distance between cluster i and cluster j, $\bar{S_{i}}$ represents mean distance from cluster mean for all observations in cluster i, while $\bar{S_{i}}$ denotes mean distance from cluster median for all observations in cluster j. An extensive analysis of existing literature reveals that most commonly used evaluation measures are accuracy and normalized mutual information.

## 9 Open-source DNA sequence analysis predictive models

The public availability of predictor source codes, pretrained language models, and word embeddings significantly benefits researchers by preventing the need to reinvent the wheel. These resources enable researchers to build on existing work, utilizing pre-trained models and complete predictive pipelines to develop new, enhanced applications. By integrating new strategies into these established pipelines, they can create more powerful predictors. In addition, open-source access to these codes allows for the reproduction of predictor performance, fostering transparency and reliability in research. To expedite the establishment of more precise, robust, reliable, and efficient AI models for DNA sequence analysis and ultimately accelerate advancements in genomics and bioinformatics research, this section provides a summary of open source predictive pipelines developed using two representation learning approaches: word embeddings and large language models for 44 different DNA sequence analysis tasks.

Our analysis reveals that, out of 39 existing word embedding based DNA sequence analysis studies, only **25** studies have made the source codes of their predictive pipelines publicly accessible. In addition, source code of only 38 studies is publicly available out of 67 existing DNA sequence analysis studies based on large language models. Tables 4, 5 offer details on open-source codes for DNA sequence analysis predictive pipelines based on word embeddings and large language models, respectively. They also provide a summary of the representation learning methods and machine/deep learning predictors employed, along with links to the corresponding source codes.

**Table 4:** Summary of open-source word embedding based models in existing studies.

**References**	**Task**	**Embedding approach**	**Classifier**	**Source Code**
Ratajczak et al. (340)	Disease genes identification	Node2Vec	MLP	https://github.com/fratajcz/speos
Pan et al. (177)	Phage-host interactions prediction	SDNE, Word2Vec	MLP	https://github.com/NWUJiePan/Code
Chen et al. (341)	Chromatin accessibility prediction	Graph2Vec	NA	https://github.com/pinellolab/simba
Han et al. (342)	Nucleosome position detection	Word2Vec	CNN + BiLSTM + BiGRU	https://github.com/lliqi-echo/Nucleosome-positioning-based-on
				-DNA-sequence-word-vector-and-deep-learning
Liao et al. (58)	Enhancer identification	Word2Vec	CNN + BiLSTM	https://github.com/WamesM/iEnhancer-DCLA
Inayat et al. (59)	Enhancer identification	FastText	MLP	https://github.com/salman-khan-mrd/IEnhancer-DFH
Zhang et al. (76)	Promoter identification	Word2Vec	RF + CNN	https://github.com/HaoWuLab-Bioinformatics/iPro-WAEL
Le et al. (78)	Promoter identification	FastText	CNN	https://github.com/khanhlee/deepPromoter
Hong et al. (82)	Enhancer-promoter interactions prediction	Word2Vec	CNN + BiGRU	https://github.com/hzy95/EPIVAN
Min et al. (49)	Enhancer-promoter interactions prediction	Word2Vec	CNN + BiGRU + Matching Heauristic	https://github.com/Xzenglab/EPI-DLMH
Dao et al. (45)	YY1-mediated chromatin loops prediction	Word2Vec	CNN	http://lin-group.cn/server/DeepYY1
Tran et al. (153)	Methylcytosine sites prediction	FastText	XGBoost	https://github.com/khucnam/5mC_Pred
Zulfiqar et al. (136)	Methylcytosine sites prediction	Word2Vec	CNN	https://github.com/linDing-groups/Deep-4mCW2V
Fang et al. (137)	Methylcytosine sites prediction	Word2Vec	CNN	https://github.com/mat310/W2VC
Khanal et al. (138)	Methylcytosine sites prediction	Word2Vec	CNN	http://nsclbio.jbnu.ac.kr/tools/4mC-w2vec/
Huang et al. (151)	Methyladenine sites prediction	Word2Vec	BiLSTM	http://39.100.246.211:5004/6mA_Pred/
Le et al. (105)	Essential genes identification	FastText	Ensemble (kNN + RF + SVM + MLP + CNN)	https://github.com/khanhlee/eDNN-EG
Zhang et al. (106)	Essential genes identification	Node2Vec	MLP	https://github.com/xzhang2016/DeepHE
Nunes et al. (110)	Disease genes prediction	OPA2Vec	RF	https://github.com/liseda-lab/KGE_Predictions_GD
Fan et al. (111)	Pseudogene function prediction	Node2Vec	GCN	https://github.com/yanzhanglab/Pseudo2GO
Yilmaz et al. (173)	Mutation susceptibility analysis	Word2Vec	Cosine similarity	https://github.com/alperyilmaz/dna2vec_snp
Arango et al. (113)	Target gene classification	FastText	MLP	https://bitbucket.org/gaarangoa/metamlp/src/master
Shi et al. (122)	Gene taxonomy classification	LSH + FastText	MLP	https://github.com/Lizhen0909/LSHVec
Li et al. (23)	DNA-binding proteins binding sites identification	Word2Vec, FastText, GloVe	CNN, DCNN, CNN + BiLSTM, DCNN + BiLSTM	http://bliulab.net/BioSeq-BLM/
Do et al. (165)	Recombination spots identification	FastText	SVM	https://github.com/khanhlee/fastspot

**Table 5:** Summary of open-source language model-based models in existing studies.

**References**	**Task name**	**Language model**	**Classifier**	**Pre-train/Self-train**	**Source code**
Zhang et al. (31)	Chromatin accessibility prediction	Transformer	Transformer	Self-train	https://github.com/ykzhang0126/semantic-CAP
Nguyen et al. (44)	Species classification, chromatin accessibility prediction	Transformer	Transformer	Self-train	https://github.com/HazyResearch/hyena-dna
Luo et al. (96)	Protein-DNA binding sites prediction	BERT	BERT	Self-train	https://github.com/lhy0322/TFBert
Liu et al. (53)	Protein-DNA binding sites prediction	BERT	CNN	Pretrained	https://github.com/YAndrewL/clape
Gao et al. (36)	Long-range chromatin interaction prediction, prediction of context-specific functional impact of genetic variants	Transformer	Transformer	Self-train	https://github.com/ZjGaothu/EpiGePT
Ni et al. (83)	Enhancer-promoter interaction prediction	Transformer	Transformer	Self-train	https://github.com/NWAFUniyu/EPI-Mind
Reddy et al. (102)	Gene expression prediction	Transformer	CNN	Self-train	https://github.com/anikethjr/promoter-models
Fishman et al. (32)	Gene expression prediction	Transformer	Transformer	Self-train	https://github.com/AIRI-Institute/GENA_LM
Osseni et al. (180)	Tumor type prediction	Transformer	Transformer	Self-train	https://github.com/dizam92/multiomic-predictions
Le et al. (281)	Methyladenine sites prediction	BERT	CNN	Self-train	https://github.com/khanhlee/bert-dna
Tsukiyama et al. (144)	Methyladenine sites prediction	BERT	CNN + BiLSTM	Self-train	https://github.com/kuratahiroyuki/BERT-6mA.git
Wang et al. (158)	Methylation sites prediction	Transformer	Transformer	Self-train	https://github.com/sb111169/tf-5mc
Zhou et al. (250)	Methylation sites prediction	Transformer	Transformer	Self-train	https://github.com/LieberInstitute/INTERACT
Jeong et al. (159)	Methylation sites prediction	BERT	BERT	Self-train	https://github.com/CompEpigen/methyl-seq_simulation
Huson et al. (248)	Methylation sites prediction	BERT	CRF	Self-train	https://github.com/husonlab/MR-DNA
Yu et al. (154)	Methylation sites prediction	BERT	BERT	Pretrained	https://github.com/YUYING07/iDNA_ABT
Zhou et al. (155)	Methylation sites prediction	BERT	BERT	Pretrained	https://github.com/wrab12/StableDNAm
Jin et al. (157)	Methylation sites prediction	BERT	FGM	Pretrained	https://github.com/FakeEnd/iDNA_ABF
Stanojevic et al. (152)	Methylcytosine sites prediction	Transformer	Transformer	Pretrained	https://github.com/lbcb-sci/rockfish
Wang et al. (282)	Methylcytosine site prediction	BERT	BERT	Self-train	https://zenodo.org/records/10143217
Yang et al. (143)	Methylcytosine sites prediction	BERT	CatBoost	Pretrained	https://github.com/abcair/4mCBERT
Yang et al. (249)	Conserved non-coding element classification	Transformer, ALBERT	Transformer, ALBERT	Self-train	https://github.com/melobio/LOGO
Fazeel et al. (29)	Nucleosome positioning prediction	BERT	LSTM	Self-train	https://github.com/FAhtisham/Nucleosome-position-prediction
Dalla et al. (100)	Promoter identification, enhancers identification, splice site identification, chromatin accessibility prediction	Transformer	Transformer	Self-train	https://github.com/instadeepai/nucleotide-transformer
Ji et al. (93)	Promoter prediction, splice sites prediction, transcription factor binding sites prediction	BERT	BERT	Self-train	https://github.com/jerryji1993/DNABERT
Xu et al. (52)	Transcription factor binding affinity prediction	BERT	BERT	Self-train	https://github.com/ericcombiolab/TRAFICA
Kabir et al. (94)	Transcription factor binding affinity prediction	BERT	CNN	Pretrained	https://github.com/lanl/EPBD-BERT
Chen et al. (343)	Transcription factor binding affinity prediction	GPT	RF	Pretrained	https://github.com/yiqunchen/GenePT
Clauwaert et al. (50)	Transcription sites prediction, translation initiation sites, methylation sites prediction	Transformer-XL	CNN	Self-train	https://github.com/jdcla/DNA-transformer
Zhang et al. (344)	Conserved non-coding element classification	GPT	GPT	Self-train	TencentAILabHealthcare/DNAGPT(github.com)
Zhang et al. (344)	Translation initiation sites identification	GPT	GPT	Self-train	https://github.com/TencentAILabHealth-care/DNAGPT
Wang et al. (61)	DNA replication origins prediction	BERT	BERT	Pretrained	https://github.com/CongWang3/PLANNER
Le et al. (60)	Enhancer identification	BERT	CNN	Pretrained	https://github.com/khanhlee/bert-enhancer
Wang et al. (61)	Enhancer identification	BERT	DF	Pretrained	https://github.com/no-banana/SMFM-master
Clauwaert et al. (91)	Gene functions prediction	Transformer	CNN	Pretrained	https://github.com/jdcla/DNA-transformer
Martinek et al. (283)	Enhancers identification, promoter identification	BERT	BERT	Pretrained	https://github.com/ML-Bioinfo-CEITEC/
Li et al. (284)	Protein-DNA interface hotspots prediction	BERT	RF	Pretrained	https://github.com/lixiangli01/PDH-EH
Toufiq et al. (114)	Candidate gene prioritization and selection	GPT	GPT	Pretrained	https://github.com/Drinchai/A37_LLM

A close look at Table 4 reveals that 25 AI-driven predictive pipelines are developed for **16** unique DNA sequence analysis tasks. These tasks include disease gene identification, phage-host interactions prediction, nucleosome position detection, enhancer identification, promoter identification, enhancer-promoter interactions prediction, YY1-mediated chromatin loop prediction, methylcytosine site prediction, methyladenine site prediction, essential gene identification, disease gene prediction, pseudogene function prediction, mutation susceptibility analysis, target gene classification, gene taxonomy classification, protein-DNA binding sites identification, and recombination spots identification. In addition, a high-level overview of Table 4 illustrates that a total of 2 node2vec and OPA2Vec word embedding approaches along with MLP and RF classifiers have made their source code publicly available for disease gene identification. In addition, source code of 4 Word2vec and FastText word embedding approach-based predictive pipelines is publicly available for promoter and enhancer identification tasks. Furthermore, two open-source FastText and node2vec word embedding approach-based predictive pipelines are developed for essential gene identification. Moreover, four Word2vec and one FastText word embeddings based predictive pipelines are developed for DNA methylation modification predictive pipelines.

Overall, Table 4 encompasses source codes of **7** unique word embedding approaches (Word2Vec, FastText, GloVe, Node2Vec, OPA2Vec, Graph2Vec, SDNE). Furthermore, a total of **3** machine learning classifiers, namely, RF, SVM, and XGBoost, **4** standalone deep learning classifiers, namely, MLP, CNN, GCN, and BiLSTM, and **6** hybrid deep learning models are used for the development of 26 predictive pipelines for 19 distinct DNA sequence analysis tasks.

Analysis of Table 5 demonstrates that 38 predictive pipelines are developed using **4** unique large language models, namely, BERT, ALBERT, GPT, and Transformer, and **9** unique classifiers, namely, RF, CatBoost, DF, MLP, CRF, CNN, LSTM, FGM, and hybrid (CNN, BiLSTM). Overall, these 38 large language models based predictive models are evaluated across **24** unique DNA sequence analysis tasks. These 24 tasks include chromatin accessibility prediction, species classification, protein-DNA binding site prediction, long-range chromatin interaction prediction, prediction of context-specific functional impact of genetic variants, enhancer-promoter interaction prediction, gene expression prediction, tumor type prediction, methyladenine modification prediction, methylation modification prediction, methylcytosine modification prediction, conserved non-coding element classification, nucleosome position prediction, promoter identification, splice site prediction, transcription factor binding site prediction, transcription factor binding affinity prediction, transcription site prediction, translation initiation site prediction, dna replication origin prediction, enhancer identification, gene function prediction, protein-dna interface hotspots prediction, and candidate gene prioritization and selection. A high level overview of Table 5 reveals that a total of two open-source chromatin accessibility predictive pipelines and two open-source gene expression prediction pipelines use transformers. In contrast, two open-source protein-DNA binding site identification pipelines and two open-source transcription factor binding site identification pipelines use BERT. In addition, three open-source transcription factor binding affinity prediction pipelines use GPT and BERT language models, whereas 8 methyl-adenine and 4 methyl-cytosine modification prediction pipelines use BERT and transformers.

Predictive pipelines can use language models in two different ways: (1) training a language model from scratch (self-training) on a large corpus and (2) leveraging a pre-trained open-source language model and fine-tuning it for specific downstream tasks. Overall, a critical analysis of existing studies reveals that source codes of 20 BERT, 13 Transformer, 4 GPT, and 1 Transformer-XL based predictive pipelines are publicly available. A holistic view of Table 5 reveals that 23 open-source predictive pipelines perform self-training of different language models from scratch for 20 tasks, whereas 15 open-source predictive pipelines have used pre-trained language models for 11 different tasks.

Specifically in 20 BERT-based predictive pipelines, 9 BERT models are self-trained from scratch for nine different tasks, namely, protein-DNA binding site prediction (96), 6mA-methyl adenine modification prediction (144, 281), DNA methylation modification prediction (159, 248), 5mC-methyl cytosine modification prediction (282), nucleosome positioning prediction (29), promoter prediction (93), splice site prediction (93), transcription factor binding site prediction (93), and transcription factor binding affinity prediction (52). In contrast, 11 pre-trained BERT models are utilized to perform 7 downstream tasks, namely, protein-DNA binding site prediction (53), DNA methylation modification (154, 155, 157), 4mC-methyl cytosine modification prediction (143), transcription factor binding affinity prediction (94), DNA replication origin prediction (25), enhancer identification (60, 61, 283), and protein-DNA interface hotspots prediction (284). To facilitate readers, we have summarized uniquely pre-trained language models along with pre-training data for DNA sequence analysis tasks in Table 6.

**Table 6:** Summary of uniquely pre-trained language models along with pre-training data for DNA sequence analysis tasks.

**Language model**	**Pre-trained data**	**Language model**	**Pre-trained data**	**Language model**	**Pre-trained data**
Zhang et al., Transformer (31)	Human reference Genome Sequences GRCh37 data	Stanojevic et al., Transformer (152)	893k sequences from ONT GM24385 Dataset	Elnaggar et al. BERT (345)	UniRef100 and BFD-100 datasets
Nguyen et al., Transformer (44)	Human reference Genome Sequences GRCh37 data	Clauwaert et al., Transformer-XL (50)	2.7M Genome Data for TSSs, TISs, MethSMRT for the 4mC-Methylations	Devlin et al., BERT (244)	BooksCorpus (800M words), English Wikipedia (2,500M words)
Gao et al., Transformer (36)	EpiGenomic data	Luo et al., BERT (96)	690 ChIP-Seq Dataset (20,464,149 Samples)	Lai et al., BERT (346)	PubMed abstracts
Ni et al., Transformer (83)	Human reference Genome Sequences	Le at al., BERT (281)	Cased Text in the top 104 Languages with the Largest Corpus	Yang et al., Transformer, ALBERT (249)	Human reference Genome Sequences hg19 data
Reddy et al., Transformer (102)	MPRA data	Tsukiyama et al., BERT (144)	*R. chinensis*	Zhang et al., GPT (344)	Genomes from 9 species: (*Arabidopsis thaliana, Caenorhabditis elegans, Bos taurus, Danio rerio, Drosophila melanogaster, Escherichia coli gca 001721525, Homo sapiens, Mus musculus, Saccharomyces cerevisiae*)
Fishman et al., Transformer (32)	Human T2T v2 Genome	Jeong et al., BERT (159)	HG19, MM10	Zhang et al., GPT (344)	(a) approx.10B bps (b) approx. 200B bps
Osseni et al., Transformer (180)	Omics dataset	Huson et al., BERT (248)	DNA Methylation and taxonomy Data	Cui et al., GPT (347)	NCBI text descriptions of individual genes
Wang et al., Transformer (158)	WGBS dataset	Wang et al., BERT (282)	1,825,095 Promoter Sequences	Toufiq et al., GPT 4 (114)	Co–expression gene set (M9.2) from the BloodGen3 repertoire associated with circulating erythroid cells
Zhou et al., Transformer (250)	WGBS dataset	Fazeel et al., BERT (29)	Human reference Genome Sequences with the length of sequences between 5 and 510 with 3-mer	Toufiq et al., Claud (114)	Co-expression gene set (M9.2) from the BloodGen3 repertoire associated with circulating erythroid cells
Dalla et al., Transformer (100)	A total of 850 species, whose Genomes add up to 174B nucleotides	Ji et al., BERT (93)	Human reference Genome Sequences	_	_
Clauwaert et al., Transformer (91)	9 283 204 full genome sequence	Xu et al., BERT (52)	ATAC-Seq dataset with over 13M nucleotide sequences	_	_

## 10 DNA sequence analysis predictive pipeline performance analysis

This section facilitates AI researchers by providing details of performance figures achieved over diverse benchmark datasets for all three kinds of predictive pipelines, namely, word embedding, language models, and nucleotide compositional and positional information across 44 distinct DNA sequence analysis tasks. To assist researchers for developing novel predictive pipelines, we have thoroughly analyzed literature and identified the current state-of-the-art predictor for each task. Section 3 provides categorization of 44 DNA sequence analysis tasks into 8 different categories. In this section, we have summarized the performance values of the predictive pipelines for these 44 tasks into 7 different Tables. Each Table corresponds to the predictive pipelines of tasks within one category, except for 1 Table that includes predictive pipelines related to tasks from 3 different categories. Within these Tables, highlighted predictors represent state-of-the-art performance values on public datasets across each task. Furthermore, this section also facilitates crucial information that which of the tasks of every goal offer more room for improvement through the development of more robust and effective predictive pipelines.

Table 7 summarizes the crucial details of seven DNA sequence analysis tasks classified under the hood of genome structure and stability. Overall, for genome structure and stability goal, four unique representation learning methods, namely, BERT, Transformer, Word2vec, and multi-scale convolution, in conjunction with bi-directional gated recurrent unit methods are used across seven different tasks. Similarly, six unique classifiers, namely, BERT, LSTM, Transformer, CNN+LSTM, CapsNet, and CNN, are used in seven different task predictive pipelines. Most commonly used representation learning scheme for this goal is BERT followed by Transformer. BERT is most commonly used with a self-classifier for three different tasks and used with LSTM classifier for one task. Transformer is used with only self-classifier for three different tasks. Word2vec potential is explored with CNN-based classifiers for two different tasks and multi-scale convolution in conjunction with bi-directional gated recurrent unit method is only explored with CNN classifier for one task. Overall, among all predictive pipelines, BERT with self-classifier or LSTM classifier manages to achieve top performance figures as compared to transformer-based predictive pipelines. Among all 7 tasks, genome structure analysis and long range chromatin interaction prediction tasks provide a lot of room for improvement as the performance of their predictive models fall below 70%. BERT or Transformer with CapsNet classifier-based predictive pipeline can potentially enhance the performance on either or both of these tasks.

**Table 7:** Genome structure and stability related 10 distinct DNA sequence analysis task predictive pipeline performance.

**Task type**	**Task name**	**References**	**Dataset**	**Representation learning**	**Classifier**	**Performance evaluation**
Binary classification	DNA replication origins prediction	**(** **25** **)**	**Gao et al. Dataset (** ***A. thaliana*** **)**	**BERT**	_	***A. thaliana*** **: AUROC = 0.9811**
		(329)	Wu et al. Datasets (*S. cerevisiae* Dataset, *S. pombe* Dataset, *K. lactis* Dataset, *P. pastoris* Dataset)	Word2Vec	CNN	Accuracy [*S. cerevisiae* (S1): 0.975, *S. pombe* (S2): 0.765, *K. lactis* (S3): 0.885, *P. pastoris* (S4): 0.967]; MCC [*S. cerevisiae* (S1): 0.940, *S. pombe* (S2): 0.530, *K. lactis* (S3): 0.771, *P. pastoris* (S4): 0.934]; AUC [*S. cerevisiae* (S1): 0.975, *S. pombe* (S2): 0.800, *K. lactis* (S3): 0.888, *P. pastoris* (S4): 0.981]
	Nucleosome position detection	**(** **29** **)**	**Gangi et al. Dataset 1 [a. C. elegans (CE), b. D. melanogester (DM), c. S. cerevisiae (YS), d**. ***H. sapiens*** **(HM)], Gangi et al. Dataset 2 (DM-5U, DM-PM, DM-LC, HM-5U, HM-PM, HM-LC, YS-PM, YS-WG)**	**BERT**	**LSTM**	**Dataset 1 a. CE: Acc = 90.5, Sn = 91.8, Sp = 92.1, Precision = 91.8, MCC = 80.5, AUROC = 95.8 b. DM: Acc = 85.1, Sn = 84.8, Sp = 85.6, Precision = 85.3, MCC = 70.5, AUROC = 92.4 c. YS: Acc = 100, Sn = 100, Sp = 99.8, Precision = 99.8, MCC = 99.82, AUROC = 100 d. HM: Acc = 88.3, Sn = 88.3, Sp = 88.4, Precision = 88.5, MCC = 76.8, AUROC = 94.4 Dataset 2 a. DM-5U: Acc = 69.5, Sn = 41.1, Sp = 85.8, Precision = 63.8, MCC = 30.8, AUROC = 68.3 b. DM-PM: Acc = 73.6, Sn = 40.1, Sp = 93.6, Precision = 80.4, MCC = 42.0, AUROC = 73.7 c. DM-LC: Acc = 71.3, Sn = 43.1, Sp = 90.0, Precision = 75.2, MCC = 38.7, AUROC = 72.0 d. HM-5U: Acc = 81.8, Sn = 51.6, Sp = 94.3, Precision = 80.0, MCC = 53.4, AUROC = 80.2 e. HM-LC: Acc = 91.1, Sn = 83.7, Sp = 96.1, Precision = 93.7, MCC = 81.7, AUROC = 95.1 f. HM-PM: Acc = 85.1, Sn = 75.8, Sp = 92.4, Precision = 89.1, MCC = 70.1, AUROC = 90.4 g. YS-PM: Acc = 92.4, Sn = 63.1, Sp = 97.2, Precision = 79.6, MCC = 66.3, AUROC = 93.5 h. YS-WG: Acc = 94.3, Sn = 60.3, Sp = 98.4, Precision = 94.3, MCC = 67.2, AUROC = 94.5**
	Chromatin accessibility prediction	**(** **32** **)**	**DeepSEA dataset (TF & DHS)**	**BERT**	_	**TF: AUROC = 96.81** **±** **0.1 DHS: AUROC = 92.8** **±** **0.03**
		**(** **31** **)**	**DNase-Seq experiment Data**	**Transformer**	_	**Average AUROC = 0.8977 Average AUPRC = 0.8983**
Binary classification	YY1-mediated chromatin loops prediction	(39)	1. HCT116 2. K562	Word2Vec	CNN+LSTM	K562: AUROC = 98.2, Acc = 92.9 HCT116: AUROC = 95.7, Acc = 88.5
		**(** **38** **)**	**1. HCT116 2. K562**	**MSC+BiGRU**	**CapsNet**	**10-fold cross-validation HCT116: Acc = 0.9544, AUROC = 0.9886 K562: Acc = 0.9680, AUROC = 0.9924** Independent test setting HCT116: Acc = 0.9622, AUROC = 0.9913, AUPRC = 0.9917, F1-score = 0.9564 K562: Acc = 0.9560, AUROC = 0.9912, AUPRC = 0.9920, F1-score = 0.9583
		(45)	1. HCT116 2. K562	Word2Vec	CNN	1. AUROC = 0.93 2. AUROC = 0.93
Multi-class classification	Genome structure analysis	(27)	NCycDB	BERT	_	Macro F1-score = 61.8, Weighted F1-score = 65.4
	Chromatin feature prediction	(34)	Yang et al. Datasets 1. LOGO-919, 2. LOGO-2002 (TF & DHS), LOGO-3357 (TF & DHS)	Transformer	_	LOGO-919: AUROC = 0.703 LOGO-2002: TF: AUROC = 0.954, DHSs: AUROC = 0.913, HM: AUROC = 0.883 LOGO-3357: TF: AUROC = 0.926, DHSs: AUROC = 0.928, HM: AUROC = 0.883
Interaction	Long range chromatin interaction prediction	**(** **36** **)**	**ChIP-seq Dataset**	**Transformer**	_	**AUPRC = 0.647**

Bold values highlight top performers on unique datasets related to distinct tasks.

Furthermore, Table 8 provides a high-level overview of the performance achieved by 48 predictors for 9 DNA sequence analysis tasks classified under the hood of gene expression regulation. Overall, for gene expression regulation goal, 10 unique representation learning methods, namely, ULMFiT, BERT, One-hot encoding, Word2vec, FastText, C2+NCP, Node2vec+SocDim+Grarep, ELECTRA, ALBERT, and Transformer, are used across 9 different tasks. Overall, 14 unique classifiers are employed in these predictive pipelines, namely, CNN, MLP, DF, CNN+BiLSTM, CNN+LSTM, SVM, Siamese network, DenseNet, CatBoost, TCN, XGBoost, RF+CNN, LSTM, BiGRU, and CNN+BiGRU.

**Table 8:** Gene expression regulation related 48 distinct DNA sequence analysis task predictive pipeline performance.

**Task type**	**Task name**	**References**	**Dataset**	**Representation learning**	**Classifier**	**Performance evaluation**
Binary classification	Enhancer identification	**(** **57** **)**	**Liu et al. dataset (1, 2)**	**ULMFiT**	**CNN**	1. Enhancer/ Non-Enhancer Cross-Validation: Acc = 0.946, Sn = 0.946, Sp = 0.949, MCC = 0.892 Independent: Acc = 0.843, Sn = 0.842, Sp = 0.87, MCC = 0.686 **2. Weak/ Strong-Enhancer Cross-Validation: Acc = 0.90, Sn = 0.90, Sp = 0.896, MCC = 0.8 Independent: Acc = 0.875, Sn = 0.873, Sp = 0.75, MCC = 0.774**
		**(** **56** **)**	**Liu et al. dataset 1**	**BERT**	**CNN**	**Sn = 1.00, Sp = 1.00, Acc = 1.00, MCC = 1.00, AUROC = 1.00**
		**(** **55** **)**	**1. DiseaseEnhancer 2. EnDisease 3. CancerEnD**	**One-hot Encoding**	**MLP**	**1. AUROC = 0.9645** **±** **0.0057, AUPRC = 0.9647** **±** **0.0043, Acc = 0.8986** **±** **0.0169, Precision = 0.8765** **±** **0.0266, Recall = 0.9290** **±** **0.0132, F1-score = 0.9018** **±** **0.0169 2. AUROC = 0.9546** **±** **0.0036, AUPRC = 0.9474** **±** **0.0118, Acc = 0.8959** **±** **0.0141, Precision = 0.8583** **±** **0.0261, Recall = 0.9469** **±** **0.0103, F1-score = 0.9003** **±** **0.0182 3. AUROC = 0.9755** **±** **0.0026, AUPRC = 0.9673** **±** **0.0047, Acc = 0.9306** **±** **0.0053, Precision = 0.9373** **±** **0.0051, Recall = 0.9261** **±** **0.0085, F1-score = 0.9317** **±** **0.0053**
		**(** **67** **)**	Liu et al. dataset 1, **Basith et al. dataset (HEK293, NHEK, K652, GN12878, HMEC, HSMM, NHLF, HUVEC)**	**BERT**	_	(Liu et al. dataset 1) Acc = 0.8300, Sn = 0.8000, Sp = 0.8600, MCC = 0.6612, AUROC = 0.8560 **(Basith's dataset) HEK293: Acc = 0.8732, Sn = 0.8666, Sp = 0.8798, MCC = 0.7283, AUROC = 0.9443 NHEK: Acc = 0.7766, Sn = 0.7229, Sp = 0.8303, MCC = 0.5453, AUROC = 0.8716 K652: Acc = 0.7974, Sn = 0.8180, Sp = 0.7767, MCC = 0.5679, AUROC = 0.8712 GM12878: Acc = 0.8222, Sn = 0.7564, Sp = 0.8879, MCC = 0.6475, AUROC = 0.9179 HMEC: Acc = 0.7645, Sn = 0.7638, Sp = 0.7652, MCC = 0.5068, AUROC = 0.8631 HSMM: Acc = 0.7193, Sn = 0.7191, Sp = 0.7194, MCC = 0.4179, AUROC = 0.7948 NHLF: Acc = 0.7884, Sn = 0.8236, Sp = 0.7532, MCC = 0.5479, AUROC = 0.8623 HUVEC: Acc = 0.7334, Sn = 0.7691, Sp = 0.6977, MCC = 0.4417, AUROC = 0.8045**
		(61)	Liu et al. dataset	BERT	DF	AUROC = 0.808, Acc = 0.822, MCC = 0.655, Sn = 0.834, Sp = 0.810
		(58)	Liu et al. dataset (1, 2)	Word2Vec	CNN + BiLSTM	1: Acc = 83.32, Sn = 84.18, Sp = 82.45, MCC = 0.6668 2: Acc = 83.30, Sn = 89.27, Sp = 77.33, MCC = 0.6736
		(65)	Liu et al. dataset (1, 2)	FastText	LSTM + CNN	Enhancer Prediction: Acc = 0.7525, MCC = 0.5051; Enhancer type Prediction: Acc = 0.6972, MCC = 0.3954;
		(59)	Liu et al. dataset (1, 2)	FastText	MLP	1: Sn = 85.81, Sp = 86.35, Acc = 86.07, MCC = 0.722; 2: Sn = 69.90, Sp = 69.32, Acc = 69.59, MCC = 0.392
		(66)	Liu et al. dataset (1, 2)	Word2Vec	CNN	1: Acc = 0.784, Sn = 0.811, Sp = 0.758, MCC = 0.567; 2: Acc = 0.749, Sn = 0.961, Sp = 0.537, MCC = 0.505
		(60)	Liu et al. dataset	BERT	CNN	Sn = 80, Sp = 71.2, Acc = 75.6, MCC = 0.514
		(47)	Liu et al. dataset (1, 2)	Word2Vec	CNN	1: Sn = 75.88, Sp = 88.88, Acc = 80.63, MCC = 0.6929, AUROC = 0.8957 2: Sn = 73.64, Sp = 76.80, Acc = 76.43, MCC = 0.4505, AUROC = 0.8109
		(64)	Liu et al. dataset (1, 2)	FastText	SVM	Cross-validation 1: Sn = 81.1, Sp = 83.5, Acc = 82.3, MCC = 0.65 2: Sn = 75.3, Sp = 60.8, Acc = 68.1, MCC = 0.37 independent 1: Sn = 82, Sp = 76, Acc = 79, MCC = 0.58 2: Sn = 74, Sp = 53, Acc = 63.5, MCC = 0.28
	Promoter identification	**(** **77** **)**	**Yang et al. dataset**	**One-hot encoding**	**Siamese network**	**Acc = 96.80, Sn = 95.08, Sp = 98.56, MCC = 0.9367**
		(251)	Xiao et al. dataset 1	BERT	_	MCC = 0.81, AUPRC = 0.98
		**(** **331** **)**	**Xiao et al. dataset (1, 2)**	***C***2+***NCP***	**DenseNet**	**1: Sn = 0.9389** **±** **0.0106, Sp = 0.9471** **±** **0.0117, Acc = 0.9429** **±** **0.0038, MCC = 0.8865** **±** **0.0076, AUROC = 0.9774** **±** **0.0017, F1-score = 0.9432** **±** **0.0040 2: Sn = 0.8711** **±** **0.0168, Sp = 0.9211** **±** **0.0088, Acc = 0.8967** **±** **0.0086, MCC = 0.7947** **±** **0.0165, AUROC = 0.9353** **±** **0.0042, F1-score = 0.8880** **±** **0.0117**
		**(** **236** **)**	**Wang et al., Datasets (1-8)**	***Node*2*Vec*+** ***SocDim*+** **GraRep**	**CatBoost**	**1**. ***H. Sapiens*****-I: Sn = 0.9565, Sp = 0.9782, Acc = 0.9673, MCC = 0.9350, Precision = 0.9772, F1-score = 0.9670, AUROC = 0.9952 2**. ***H. Sapiens*****-II: Sn = 0.8646, Sp = 0.9849, Acc = 0.9248, MCC = 0.8558, Precision = 0.9829, F1-score = 0.9199, AUROC = 0.9844 3**. ***R. Norvegicus*****-I: Sn = 0.9113, Sp = 0.9757, Acc = 0.9424, MCC = 0.8908, Precision = 0.9761, F1-score = 0.9425, AUROC = 0.9975 4**. ***R. Norvegicus*****-II: Sn = 0.8905, Sp = 0.9849, Acc = 0.9377, MCC = 0.8793, Precision = 0.9833, F1-score = 0.9346, AUROC = 0.9832 5**. ***D. melanogaster*****-I: Sn = 0.9350, Sp = 0.9670, Acc = 0.9560, MCC = 0.9123, Precision = 0.9662, F1-score = 0.9555, AUROC = 0.9923 6**. ***D. melanogaster*****-II: Sn = 0.9425, Sp = 0.7819, Acc = 0.8596, MCC = 0.7281, Precision = 0.8112, F1-score = 0.8697, AUROC = 0.9448 7**. ***Z. mays*****-I: Sn = 0.934, Sp = 0.9450, Acc = 0.9395, MCC = 0.8791, Precision = 0.9444, F1-score = 0.9392, AUROC = 0.9841 8**. ***Z. mays*****-II: Sn = 0.9516, Sp = 0.7806, Acc = 0.8661, MCC = 0.7433, Precision = 0.8127, F1-score = 0.8767, AUROC = 0.9485**
		(235)	Xiao et al. dataset 1	Word2Vec	TCN	Acc = 91.86, Sn = 92.74, Sp = 91
		**(** **348** **)**	**Ji et al. Dataset**	**BERT**	_	**Acc = 0.8613, AUROC = 0.9354, MCC = 0.7226, Precision = 0.8569, Recall = 0.8624**
		**(** **349** **)**	**Liu et al. dataset (1, 2)**	**BERT**	**XGBoost**	**1. Promoter Identification: Sn = 84.34, Sp = 86.56, Acc = 85.45 2. Promoter strength classification: Sn = 70.85, Sp = 81.63, Acc = 76.92**
		(89)	Human dataset	ELECTRA	_	Acc = 0.862, AUROC = 0.935, F1-score = 0.862, MCC = 0.725, Precision = 0.863, Recall = 0.862
		**(** **76** **)**	**1. K562 2. GM12878 3. HeLa-S3 4. HUVEC**	**Word2Vec**	**RF + CNN**	**1. AUROC = 0.9809, Acc = 0.9415, MCC = 0.8831 2. AUROC = 0.9783, Acc = 0.9334, MCC = 0.8668 3. AUROC = 0.9824, Acc = 0.9374, MCC = 0.8749 4. AUROC = 0.9847, Acc = 0.9481, MCC = 0.8963**
		(48)	Xiao et al. dataset	Word2Vec	LSTM	Acc = 90.59, MCC = 0.8114, Sn = 90.28, Sp = 90.94
		(249)	Yang et al. dataset	ALBERT	_	AUROC = 0.743
		(90)	Ji et al. dataset	BERT	CNN	Precision = 0.805, Recall = 0.803, Acc = 0.894
		(90)	Ji et al. dataset	BERT	_	Precision = 0.805, Recall = 0.803, Acc = 0.894
		(34)	Yang et al. dataset	Transformer	_	Recall = 0.921, Precision = 0.940, F1-score = 0.933
		(93)	Human dataset	BERT	_	AUROC = 0.981, AUPRC = 0.982
		(79)	Xiao et al. dataset (1, 2)	Word2Vec	CNN	1: Acc = 91.42 2: Acc = 82.42
		(78)	Xiao et al. dataset (1, 2)	FastText	CNN	1: Acc = 85.41 2: Acc = 73.1
	Transcription sites prediction	**(** **50** **)**	**Clauwaert et al. Dataeset**	**Transformer-XL**	**CNN**	**AUROC = 0.977**
	Transcription factor binding sites prediction	**(** **94** **)**	**ChIP-Seq Dataset**	**BERT**	**CNN**	**AUROC = 0.949, AUPRC = 0.326**
		**(** **89** **)**	**690 ChIP-Seq dataset**	**ELECTRA**	_	**Acc = 0.856, AUROC = 0.935, F1-score = 0.851, MCC = 0.727, Precision = 0.859, Recall = 0.856**
		**(** **90** **)**	**TF ChIP-Seq dataset**	**BERT**	_	**Precision = 0.937, Recall = 0.935, Acc = 0.989**
		**(** **91** **)**	**TSSs dataset**	**Transformer**	**CNN**	**AUROC = 0.981. AUPRC = 0.141**
		**(** **93** **)**	**497 TF ChIP-Seq dataset**	**BERT**	_	**Mean Acc = 0.903, Mean AUROC = 0.954, Mean F1-score = 0.901, Mean MCC = 0.807, Mean Precision = 0.898, Mean Recall = 0.909**
		(92)	1. A549 dataset 2. MCF-7 dataset 3. H1-HESC dataset 4. HUVEC dataset	Word2Vec	BiGRU	HUVEC: Average Precision = 0.9618, Average AUROC = 0.9608 MCF7: Average Precision = 0.9653, Average AUROC = 0.9643 A549: Average Precision = 0.9608, Average AUROC = 0.9593 H1-HESC: Average Precision = 0.9528, Average AUROC = 0.9524
	Splice sites prediction	(97)	Splice-junction Gene sequence dataset	_	LSTM	Acc = 0.98, Precision = 0.98, Recall = 0.99, F1-score = 0.98
		**(** **98** **)**	**Degroeve et al. Dataset**	_	**CNN**	**F1-score = 0.9187** **±** **0.0070, MCC = 0.9028** **±** **0.0085**
		**(** **99** **)**	**Liu et al. Dataset**	**One-hot Encoding**	**MLP**	**Donor: Acc = 96.57 Acceptor: Acc = 95.82**
		**(** **100** **)**	**Wang et al. Dataset**	**Transformer**	_	**AUPRC = 0.984**
		**(** **93** **)**	**Ji et al. Dataset**	**BERT**	_	**Acc = 0.939, AUROC = 0.992, F1-score = 0.937, MCC = 0.903, Precision = 0.961, Recall = 0.919**
	Translation initiation site prediction	(101)	Kalkatawi et al. TIS dataset	k-mer Embedding	Bi-GRU	Acc = 96.40, AUROC = 96.40, AUPRC = 94.87
		**(** **50** **)**	**TIS dataset**	**Transformer-XL**	**CNN**	**AUROC = 0.998**
Interaction	Enhancer-promoter interactions prediction	**(** **350** **)**	**Whalen et al. datasets (GM12878, HUVEC, HeLa-S3, IMR90, K562, NHEK)**	**GCN, K-mer**	**GCN**	**F1-score: GM12878 = 0.8679, HUVEC = 0.8954, HeLa-S3 = 0.9175, IMR90 = 0.7949, NHEK = 0.6085**
		(233)	Whalen et al. datasets (HMEC, IMR90, K562, NHEK)	Transformer	Multi-Scale CNN	HMEC: AUROC = 0.9344, AUPRC = 0.6852, IMR90 AUROC = 0.8936, AUPRC = 0.5878, K562 AUROC = 0.8513, AUPRC = 0.2101, NHEK AUROC = 0.8243, AUPRC = 0.4760
		(332)	Zheng et al. Datasets (GM12878, HeLa)	Deeptools	RF	1. Sn = 0.578, Sp = 0.964, Precision = 0.799, Acc = 0.887, AUROC = 0.919, AUPRC = 0.773, 2. Sn = 0.363, Sp = 0.953, Precision = 0.660, Acc = 0.836, AUROC = 0.831, AUPRC = 0.601
		(177)	ESKAPE dataset	SDNE, Word2Vec	MLP	Acc = 86.65 ± 1.55, Sn = 88.40 ± 1.81, Sp = 84.91 ± 1.96, Precision = 85.43 ± 1.74, F1-score = 86.88 ± 1.53, AUROC = 0.9208 ± 0.0119
		**(** **249** **)**	**Yang et al. Datasets (FoeT, Mon, nCD4, tB, tCD4, tCD8)**	**Transformer**	**MLP**	**FoeT: AUPRC = 0.9447, Mon: AUPRC = 0.9414, nCD4: AUPRC = 0.9457, tB: AUPRC = 0.9474, tCD4: AUPRC = 0.9475, tCD8: AUPRC = 0.9387**
		(82)	Whalen et al. Datasets (GM12878, HUVEC, HeLa-S3, IMR90, K562, NHEK)	Word2Vec	CNN + BiGRU	GM12878: AUROC = 0.965, AUPRC = 0.819, HUVEC: AUROC = 0.950, AUPRC = 0.773, HeLa-S3: AUROC = 0.960, AUPRC = 0.820, IMR90: AUROC = 0.962, AUPRC = 0.801, K562: AUROC = 0.959, AUPRC = 0.814, NHEK: AUROC = 0.985, AUPRC = 0.899
		(49)	Whalen et al. Datasets (GM12878, HUVEC, HeLa-S3, IMR90, K562, NHEK)	Word2Vec	CNN+BiGRU	GM12878: AUROC = 0.949, AUPRC = 0.819, F1-score = 0.766, HUVEC: AUROC = 0.948, AUPRC = 0.720, F1-score = 0.649, HeLa-S3: AUROC = 0.952, AUPRC = 0.824, F1-score = 0.78, IMR90: AUROC = 0.948, AUPRC = 0.818, F1-score = 0.778, K562: AUROC = 0.955, AUPRC = 0.826, F1-score = 0.795, NHEK: AUROC = 0.977, AUPRC = 0.893, F1-score = 0.861
	Protein-DNA binding sites prediction	**(** **53** **)**	**Patiyal et al. dataset (dataset 1) Xia et al. dataset (dataset 2)**	**BERT**	**CNN**	**dataset 1: TE46 Sp = 0.835, Recall = 0.747, Precision = 0.306, F1-score = 0.434, MCC = 0.401, AUROC = 0.871 dataset 2: TE129 Sp = 0.955, Recall = 0.464, Precision = 0.396, F1-score = 0.427, MCC = 0.389, AUROC = 0.881**
		(96)	**690 ChIP-Seq Dataset**	**BERT**	_	**AUROC = 0.947** **±** **0.041, Acc = 0.880** **±** **0.062, Precision = 0.882** **±** **0.061, Recall = 0.880** **±** **0.062, F1-score = 0.880** **±** **0.062, MCC = 0.762** **±** **0.122**
		**(** **95** **)**	**Liu et al., Dataset, Tian et al., Dataset**	**BERT**	**RF**	**dataset 1: Sp = 0.529, Precision = 0.106, Recall = 0.574, F1-score = 0.179, AUROC = 0.551, MCC = 0.025 dataset 2: Sp = 0.724, Precision = 0.119, Recall = 0.536, F1-score = 0.194, AUROC = 0.630, MCC = 0.067**
Regression	Transcription factor binding affinity prediction	**(** **52** **)**	**1. Weirauch et al. dataset, 2. Jolma et al. dataset**	**BERT**	_	**1: Average PCC = 0.649 2: Average R2 = 0.977**

Bold values highlight top performers on unique datasets related to distinct tasks.

For this goal, most commonly used representation learning approach is BERT. BERT is used with five different classifiers across six unique tasks. BERT with self-classifier is evaluated across all six unique tasks, whereas BERT with other four classifiers is evaluated on some of these six tasks. Specifically, BERT with CNN classifier is evaluated on four common tasks, namely, enhancer identification, promoter identification, protein-DNA binding site prediction, and transcription factor binding site prediction. BERT with DF, RF, and XGBoost is evaluated on one common task each including enhancer identification, protein-DNA binding site prediction, and promoter identification. Among all BERT-based predictive pipelines, BERT achieves the best performance with CNN classifier on two tasks, namely, transcription factor binding site prediction and protein-DNA binding site prediction. Second most common representation learning approach for this goal is Word2vec that is explored with seven different classifiers for four different tasks. Specifically, Word2Vec with CNN and CNN+BiLSTM is used for one task, with LSTM, TCN, and RF+CNN for one task, with BiGRU for one task, and with CNN+BiGRU for one task. Among all Word2vec-based predictive pipelines, Word2Vec achieves the best performance with CNN+BiGRU classifier on enhancer-promoter interaction prediction task. From two most common approaches, BERT with CNN and self-classifiers manages to yield top performance values as compared to Word2vec-based predictive pipelines. Beyond BERT and Word2vec, transformer-XL is used with CNN for two different tasks, ALBERT and ELECTRA are used with self-classifiers for a single task, and ULMFiT is used with CNN for a single task. In addition, potential of transformer is explored with CNN for one task and with a self-classifier for two tasks. FastText-based representation learning is used with MLP and SVM classifiers for a single task and potential of three graph embedding, namely, Node2Vec, SocDim, and GraRep, is explored with CatBoost classifier for a single task. Overall, among all approaches, ULMFiT manages to achieve best performance with CNN classifier on enhancer identification task. From all nine tasks, protein-DNA binding site prediction and protein-DNA binding affinity prediction have some room for improvement. Considering the promising performance trends for this goal, Word2vec potential can be explored with CNN+BiGRU classifier and ULMFiT potential can be explored with standalone CNN as well as ensemble CNN+BiGRU classifier to enhance the performance of under-performing tasks.

In addition, Table 9 summarizes predictive models developed for seven different DNA sequence analysis tasks classified under the hood of gene analysis. For gene analysis goal, 12 unique representation learning methods are used that include Gapped K-mer Encoding, ESM-2, Flux Sampling, Node2Vec, FastText, OPA2Vec, Transformer, Laplacian eigenmaps + Locally linear Embedding + DeepWalk + Node2Vec, and GPT. Overall, eight unique classifiers, namely, GCNN, GNN, GAT, kNN, RF, SVM, CNN, and MLP, are used in different predictive pipelines. Most commonly used representation learning scheme for this goal is Node2vec followed by FastText. Node2vec is used with GCNN classifier for three different tasks and with GAT and MLP classifiers for two different tasks, whereas FastText is used with an ensemble and MLP classifiers for two different tasks. From most commonly used approaches, Node2Vec performs better in the majority of tasks and achieve top performance with GAT classifier as compared to FastText approach. Apart from Node2Vec and FastText, potential of ESM-2 along with a self-classifier and flux sampling with GNN is explored for a single task, transformer with CNN is evaluated for two different tasks, and GPT is used with a self-classifier for two tasks. In addition, OPA2Vec is used with RF for one task, Gapped K-mer encoding with GCN classifier is used for one task, and potential of Laplacian eigenmaps, Locally linear Embedding, DeepWalk, and Node2Vec is explored with RF for one task. In the holistic view, among all approaches, Gapped K-mer Encoding approach with GCNN classifier achieves the best performance for essential gene identification task. Among all seven distinct tasks, multi-label classification task, namely, gene function prediction provides a lot of room for improvement as the performance of its respective predictive pipeline based on Node2vec and GCNN classifier fall below 60%. Considering the promising performance of Gapped K-mer Encoding method, Gapped K-mer Encoding method and GCNN-based predictive pipeline can prove fruitful for various low performance tasks such as gene function prediction.

**Table 9:** Gene analysis related 15 distinct DNA sequence analysis task predictive pipeline performance.

**Task type**	**Task name**	**References**	**dataset**	**Representation learning**	**Classifier**	**Performance evaluation**
Binary classification	Essential genes identification	**(** **333** **)**	**Hu et al. dataset (D. melanogaster, M. maripaludis**, ***H. sapiens, C. elegans)***	**Gapped K-mer encoding**	**GCNN**	**1. Sn = 0.8333, Sp = 0.9939, Acc = 0.9847, MCC = 0.8545, AUROC = 0.8283 2. Sn = 0.9052, Sp = 0.9304, Acc = 0.9221, MCC = 0.8265, AUROC = 0.8422 3. Sn = 0.9048, Sp = 0.9566, Acc = 0.9501, MCC = 0.7961, AUROC = 0.8655 4. Sn = 0.8362, Sp = 0.9368, Acc = 0.9242, MCC = 0.6983, AUROC = 0.7834**
		(109)	Ma et al. dataset (*S.cerevisiae, E.coli, H.sapiens, D.melanogaster*)	ESM-2	_	_
		**(** **104** **)**	**Campos et al. dataset**	**Flux sampling**	**GNN**	**Acc = 0.871** **±** **0.012, Precision = 0.769** **±** **0.030, Recall = 0.718** **±** **0.037, F1-score = 0.743** **±** **0.023**
		**(** **108** **)**	**Ma et al. dataset (*****S. cerevisiae, E. coli***, ***H. sapiens***, ***D. melanogaster*****)**	**Node2Vec**	**GAT**	**AUROC S. cerevisiae: (DIP: 76.57** **±** **0.74, BioGrid: 87.66** **±** **2.58, STRING: 90.13** **±** **1.08); E.coli: (DIP: 79.96** **±** **2.20, BioGrid: 92.35** **±** **1.15, STRING: 97.02** **±** **0.50); H.sapiens: (DIP: 75.61** **±** **0.90, BioGrid: 88.39** **±** **0.52, STRING: 90.95** **±** **0.54); D.melanogaster: (DIP: 32.90** **±** **5.34, BioGrid: 78.78** **±** **4.24, STRING: 77.82** **±** **1.95)**
		(105)	Hu et al. dataset	FastText	kNN + RF + SVM + CNN	Sn = 60.2, Sp = 84.6, Acc = 76.3, MCC = 0.449, AUROC = 0.814
		(106)	Zhang et al. dataset	Node2Vec	MLP	AUROC = 94.15, Sp = 94.75, Average Precision = 90.64, Acc = 90.88
		(107)	Xiao et al. dataset	Node2Vec	MLP	Average AUROC = 95.17, AUPRC = 92.21, Acc = 91.59, F1-score = 78.71
Binary classification	Target gene classification	(113)	Argoty et al. dataset	FastText	MLP	Precision = 0.99, Recall = 0.99
Binary classification	Disease genes prediction	(110)	Nunes et al. dataset	OPA2Vec	RF	Median WAF score = 0.768
Multi-label classification	Gene function prediction	**(** **112** **)**	**(1). 1,000 human gene set from gene ontology, (2). 100 omic gene set**	**GPT**	**_**	**Semantic similarity = 0.50, Gene covered = 30**
Multi-class classification	Gene expression prediction	**(** **102** **)**	**Reddy et al. dataset (Jurkat, K-562, THP-1)**	**Transformer**	**CNN**	**(1). Jurkat PCC = 0.6389** **±** **0.0036, SRCC = 0.5996** **±** **0.0093 (2). K-562 PCC = 0.6152** **±** **0.0082, SRCC = 0.6043** **±** **0.0045 (3). THP-1 PCC = 0.5672** **±** **0.0131, SRCC = 0.4742** **±** **0.0136**
		(103)	Al Taweraqi et al. dataset	Laplacian eigenmaps + Locally linear Embedding + DeepWalk + Node2Vec	RF	_
Multi-class classification	Pseudo-gene function prediction	(111)	Fan et al. dataset (CC, MF, BP)	Node2Vec	GCN	CC: (AUPRC = 0.587 ± 0.02, F1-score = 0.380 ± 0.01) MF: (AUPRC = 0.463 ± 0.02, F1-score = 0.319 ± 0.01) BP: (AUPRC = 0.362 ± 0.01, F1-score = 0.193 ± 0.01)
Multi-class classification	Candidate gene prioritization & Selection	(114)	Toufiq et al. dataset	GPT	_	_

Bold values highlight top performers on unique datasets related to distinct tasks.

Moreover, Table 10 summarizes eight DNA sequence analysis tasks classified across three unique biological goals, namely, DNA modification prediction, environmental and microbial genomics, and gene network analysis. For DNA modification prediction goal, across 4 DNA sequence analysis tasks, 27 predictors are developed. In the predictive pipelines, overall 10 unique representation learning methods are used which include PSeKNC, BERT, nucleotide physico-chemical properties and occurrence frequency based encoder, Transformer-XL, Word2Vec, One-hot encoding, FastText, ULMFIT, and BERT+ ALBERT+XLNet+ ELECTRA. Similarly, nine unique classifiers, namely, Structural Sparse Regularized Random Vector Functional Link Network, CatBoost, KNN, CNN, BiLSTM, CNN+BiLSTM, SVM, XGBoost, and FGM, are employed in different predictive pipelines. For DNA modification prediction goal, most commonly used representation learning approach is BERT. BERT is used with five different classifiers for all four tasks. Specifically, BERT with a self-classifier is evaluated for three tasks, namely, 4mC-methyl cytosine, 5mC-methyl cytosine, and DNA methylation modification prediction. BERT is also used with two other classifiers, namely, CatBoost, and FGM, for two common tasks, namely, 4mC-methyl cytosine modification prediction, and DNA methylation modification prediction. In addition, BERT is used with CNN and CNN+BiLSTM classifier for one task, namely, 6mA-methyl adenine modification prediction. Among all BERT-based predictive pipelines, BERT with a self-classifier showed top performance values for two tasks, namely, 5mC-methyl cytosine modification prediction, and DNA methylation modification prediction.

**Table 10:** Distinct predictive pipeline performance related to DNA modification, environmental and microbial genomics tasks, and gene network analysis.

**Task type**	**Task name**	**References**	**Dataset**	**Representation learning**	**Classifier**	**Performance evaluation**
**Goal: DNA modification prediction**						
Binary classification	4-Methylcytosine (4mc) modification prediction	**(** **134** **)**	**Chen et al. datasets (1-6)**	**PseKNC**	**Structural Sparse Regularized Random Vector Functional Link Network**	**1. Acc = 0.8761, Sn = 0.8630, Sp = 0.8893, MCC = 0.7530 2. Acc = 0.8753, Sn = 0.8739, Sp = 0.8768, MCC = 0.7512 3. Acc = 0.8278, Sn = 0.8256, Sp = 0.8301, MCC = 0.6566 4. Acc = 0.9601, Sn = 0.8641, Sp = 0.9562, MCC = 0.9210 5. Acc = 0.9011, Sn = 0.8895, Sp = 0.9127, MCC = 0.8031 6. Acc = 0.9139, Sn = 0.9087, Sp = 0.9190, MCC = 0.8289**
		**(** **143** **)**	**Yang et al. dataset (** ***A. thaliana, C.elegans, D. melanogaster, E. coli, G. pickeringii, G. subterraneous*** **)**	**BERT**	**CatBoost**	**A. thaliana: MCC = 0.6954, F1-score = 0.8521 C. elegans: MCC = 0.8326, F1-score = 0.9183 D. melanogaster: MCC = 0.7924, F1-score = 0.8986 E. coli: MCC = 0.9356, F1-score = 0.9679 G. pickeringii: MCC = 0.8904, F1-score = 0.9431 G. subterraneous: MCC = 0.8796, F1-score = 0.9363**
		(351)	Chen et al. dataset	BERT	_	AUROC = 0.897, AUPRC = 0.907
		**(** **135** **)**	**Khanal et al. dataset (** ***C. elegans, D. melanogaster, A. thaliana, E. coli, G. subterraneus, G. pickeringi, F. vesca, R. chinensis*** **)**	**Number of codons, Number of occurrences of each codon, Proportion of each codon, Number of Nucleotides, Average number of Nucleotides per codon, Percentage of GC, Percentage of purines AG, Percentage of pyrimidines CT, Percentage of AT, Molecular weight of the sequence, Melting temperature, Proportion of Nucleotide DNA sequence, Protein sequence from DNA sequence, Number of amino acids, Percentage of amino acids, Aromaticity, Instability index, Isoelectric point, Molecular weight of Portion, Gravy**	**KNN**	***C.elegans*****: Acc = 92.20, AUROC = 91.99, Precision = 89.47, Recall = 95.67** ***D. melanogaster*****: Acc = 92.79, AUROC = 92.80, Precision = 88.54, Recall = 98.30** ***A. thaliana*****: Acc = 90.27, AUROC = 90.28, Precision = 87.52, Recall = 93.93** ***E. coli*****: Acc = 91.02, AUROC = 91.03, Precision = 86.36, Recall = 97.43** ***G. subterraneus*****: Acc = 93.09, AUROC = 93.09, Precision = 91.48, Recall = 95.02** ***G. pickeringi*****: Acc = 90.78, AUROC = 90.79, Precision = 87.20, Recall = 95.61 F. vesca: Acc = 90.67, AUROC = 90.68, Precision = 85.31, Recall = 98.26** ***R. chinensis*****: Acc = 91.87, AUROC = 91.88, Precision = 87.35, Recall = 97.93**
		**(** **136** **)**	**Zulifiqar et al. dataset**	**Word2Vec**	**CNN**	**Acc = 0.946, Sn = 0.938, Sp = 0.881, MCC = 0.778, AUROC = 0.989**
		**(** **50** **)**	**Clauwaert et al. dataset**	**Transformer-XL**	**CNN**	**AUROC = 0.985**
		(138)	Khanal et al. dataset (*F. vesca, R. chinensis*)	Word2Vec	CNN	F. vesca: Sn = 0.8976, Sp = 0.8417, Acc = 0.8697, MCC = 0.7407, AUROC = 0.9400 R. chinensis: Sn = 0.8219, Sp = 0.8854, Acc = 0.8541, MCC = 0.7093, AUROC = 0.9370
		**(** **137** **)**	**Zeng et al. dataset**	**Word2Vec**	**CNN**	**Acc = 0.9321, MCC = 0.8559, Sn = 0.9508, Sp = 0.9161, AUROC = 0.9712**
	Methyladenine (6ma) modification Prediction	**(** **149** **)**	**1**. ***A. thaliana*** **2**. ***D. melanogaster*** **3. 6mA-rice-Chen 4. 6mA-rice-Lv 5. Rosaceae**	**One-hot Encoding**	**BiLSTM**	**1. Sn = 0.896, Sp = 0.935, Acc = 0.915, MCC = 0.831, AUROC = 0.967 2. Sn = 0.903, Sp = 0.952, Acc = 0.927, MCC = 0.855, AUROC = 0.963 3. Sn = 0.850, Sp = 0.917, Acc = 0.882, MCC = 0.763, AUROC = 0.947 4. Sn = 0.947, Sp = 0.930, Acc = 0.938, MCC = 0.877, AUROC = 0.976 5. Sn = 0.962, Sp = 0.961, Acc = 0.962, MCC = 0.924, AUROC = 0.990**
		**(** **150** **)**	**1. Homo sapiens dataset (Train, Independent) 2. Mus musculus dataset**	**Transformer**	**CNN**	**Homo sapiens (Train): Acc = 96.5 Homo sapiens (Independent): Acc = 93.75 Mus musculus: Acc = 96.86**
		(144)	Lv et al. dataset (*A. thaliana, C. elegans, C. equisetispolia, D. melanogaster, F. vesva, H. sapiens, R. chinensis, S. cerevisiae, T. thermophilus*, Ts. SUP5-1, Xoc. BLS256)	BERT	CNN + BiLSTM	*A. thaliana*: AUROC = 0.927 C. elegans: AUROC = 0.962 *C. equisetifpolia*: AUROC = 0.800 *D. melanogaster*: AUROC = 0.967 *F. vesca*: AUROC = 0.976 *H. sapiens*: AUROC = 0.963 *R. chinensis*: AUROC = 0.876 *S. cerevisiae*: AUROC = 0.892 *T. thermophilus*: AUROC = 0.938 Ts. SUP5-1: AUROC = 0.829 Xoc. BLS256: AUROC = 0.937
		**(** **281** **)**	**DNA 6 mA dataset**	**BERT**	**CNN**	**Cross-Validation Sn = 86.4, Sp = 68.8, Acc = 77.6, MCC = 0.651 Independent Sn = 84.3, Sp = 73.1, Acc = 79.3, MCC = 0.580**
		(145)	*A. thaliana* dataset	Transformer	_	Acc = 0.9633, Sn = 0.9655, Sp = 0.9611, MCC = 0.9266
		(151)	1. Rice dataset 2. Mus musculus dataset	Word2Vec	BiLSTM	Rice dataset: Sn = 95.66, Sp = 92.38, Acc = 94.02, MCC = 0.88, AUROC = 0.981 Mus musculus: Sn = 93.28, Sp = 100, Acc = 96.73, MCC = 0.93
		(234)	Chen et al. dataset	FastText	SVM	Sn = 86.48, Sp = 89.09, Acc = 87.78, MCC = 0.756
	5-methylcytosine (5mc) modification prediction	**(** **282** **)**	**Wang et al. dataset**	**BERT**	_	**Acc = 0.932, MCC = 0.653, AUROC = 0.966**
		(153)	Wang et al. dataset	FastText	XGBoost	Acc = 91.8, Sp = 92.0, Sn = 89.9, MCC = 0.626, AUROC = 0.962
		**(** **152** **)**	**Stanojevic et al. datasets (GM24385, NA12878, NA19240, HIESc, K562, HX1)**	**Transformer**	_	**GM24385: Acc = 0.9988, Precision = 0.9990, Recall = 0.9988, FPR = 0.0011, F1-score = 0.9989 NA12878: Acc = 0.9936, Precision = 0.9921, Recall = 0.9953, FPR = 0.0081, F1-score = 0.9937 NA19240: Acc = 0.9872, Precision = 0.9678, Recall = 0.9765, FPR = 0.0096, F1-score = 0.9721 H1ESc: Acc = 0.9938, Precision = 0.9995, Recall = 0.9935, FPR = 0.0040, F1-score = 0.9965 K562: Acc = 0.9972, Precision = 0.9512, Recall = 0.9964, FPR = 0.0028, F1-score = 0.9733 HX1: Acc = 0.9950, Precision = 0.9993, Recall = 0.9951, FPR = 0.0057, F1-score = 0.9972**
	Methylation modification prediction	(159)	Jeong et al. dataset	BERT	_	Precision = 0.98
		(351)	Yu et al. dataset	BERT	_	AUROC = 0.897, AUPRC = 0.907
		(155)	Lv et al. datasets (5hmc: *M. musculus, H. sapiens*, 4mc: *C. equisetifolia, F. vesca, S. cerevisiae, Tolypocladium*, 6mA: *A. thaliana, C. elegans, C. equisetifolia, D. melanogaster, F. vesca, H. sapiens, R. chinensis, S. cerevisiae, T. thermophile, Tolypocladium*, XocBLS256)	BERT	_	5hmC_*M. sapiens*: Acc = 0.949, AUROC = 0.967, MCC = 0.900 5hmC_*M. musculus*: Acc = 0.968, AUROC = 0.981, MCC = 0.936 4mC_*C. equisetifolia*: Acc = 0.853, AUROC = 0.896, MCC = 0.706 4mC_*F. vesca*: Acc = 0.853, AUROC = 0.928, MCC = 0.706 4mC_*S. cerevisiae*: Acc = 0.710, AUROC = 0.776, MCC = 0.423 4mC_Tolypocladium: Acc = 0.743, AUROC = 0.819, MCC = 0.487 6mA_*A. thaliana*: Acc = 0.861, AUROC = 0.934, MCC = 0.722 6mA_*C. elegans*: Acc = 0.909, AUROC = 0.966, MCC = 0.818 6mA_*C. equisetifolia*: Acc = 0.745, AUROC = 0.816, MCC = 0.494 6mA_*D. melanogaster*: Acc = 0.923, AUROC = 0.971, MCC = 0.846 6mA_*F. vesca*: Acc = 0.939, AUROC = 0.981, MCC = 0.878 6mA_*H. sapiens*: Acc = 0.907, AUROC = 0.969, MCC = 0.815 6mA_*R. chinensis*: Acc = 0.818, AUROC = 0.881, MCC = 0.635 6mA_*S. cerevisiae*: Acc = 0.827, AUROC = 0.905, MCC = 0.654 6mA_*T. thermophile*: Acc = 0.882, AUROC = 0.944, MCC = 0.772 6mA_*Tolypocladium*: Acc = 0.768, AUROC = 0.845, MCC = 0.538 6mA_XocBLS256: Acc = 0.877, AUROC = 0.949, MCC = 0.756
		(158)	CCLE dataset	Transformer	_	Sn = 0.831, Sp = 0.991, Acc = 0.978, MCC = 0.871, AUROC = 0.989
		**(** **156** **)**	**Lv et al. datasets (5hmc:** ***M. musculus***, ***H. sapiens*****, 4mc:** ***C. equisetifolia, F. vesca, S. cerevisiae, Tolypocladium*****, 6mA:** ***A. thaliana, C. elegans, C. equisetifolia, D. melanogaster, F. vesca***, ***H. sapiens***, ***R. chinensis, S. cerevisiae**,* ***T. thermophile***, Tolypocladium, XocBLS256	**BERT + ALBERT + XLNet + ELECTRA**	_	**(6mA)** ***T. thermophile*****: AUROC = 0.9467, Acc = 0.8840, F1-score = 0.8923, Recall = 0.9611, AUPRC = 0.9321 A. thaliana: AUROC = 0.9378, Acc = 0.8649, F1-score = 0.8615, Recall = 0.8401, AUPRC = 0.9423** ***H. sapiens*****: AUROC = 0.9687, Acc = 0.9077, F1-score = 0.9068, Recall = 0.8975**, **AUPRC = 0.9721 Xoc. BLS256: AUROC = 0.9446, Acc = 0.8742, F1-score = 0.8712, Recall = 0.8511, AUPRC = 0.9421** ***D. melanogaster*****: AUROC = 0.9730, Acc = 0.9276, F1-score = 0.9275, Recall = 0.9258, AUPRC = 0.9761 C. elegans: AUROC = 0.9684, Acc = 0.9131, F1-score = 0.9138, Recall = 0.9219, AUPRC = 0.9674 C. equisetifolia: AUROC = 0.8350, Acc = 0.7590, F1-score = 0.7481, Recall = 0.7158, AUPRC = 0.8492 S. cerevisiae: AUROC = 0.9082, Acc = 0.8325, F1-score = 0.8233, Recall = 0.7802, AUPRC = 0.9198 Tolypocladium: AUROC = 0.8669, Acc = 0.7895, F1-score = 0.7824, Recall = 0.7567, AUPRC = 0.8730 F. vesca: AUROC = 0.9821, Acc = 0.9407, F1-score = 0.9403, Recall = 0.9336, AUPRC = 0.9831 R. chinensis: AUROC = 0.9654, Acc = 0.9164, F1-score = 0.9167, Recall = 0.9197, AUPRC = 0.9691 (4mC) C. equisetifolia: AUROC = 0.9108, Acc = 0.8333, F1-score = 0.8272, Recall = 0.7978, AUPRC = 0.9221 F. vesca: AUROC = 0.9256, Acc = 0.8522, F1-score = 0.8554, Recall = 0.8739, AUPRC = 0.9144 S. cerevisiae: AUROC = 0.8064, Acc = 0.7376, F1-score = 0.7253, Recall = 0.6926, AUPRC = 0.8215 Tolypocladium: AUROC = 0.8149, Acc = 0.7380, F1-score = 0.7285, Recall = 0.7031, AUPRC = 0.80889 (5hmC)** ***M. musculus*****: AUROC = 0.9817, Acc = 0.9649, F1-score = 0.9651, Recall = 0.9685, AUPRC = 0.9782** ***H. sapiens*****: AUROC = 0.9680, Acc = 0.9484, F1-score = 0.9500, Recall = 0.9787, AUPRC = 0.9485**
		(157)	Lv et al. datasets (5hmc: *M. musculus, H. sapiens*, 4mc: *C. equisetifolia, F. vesca, S. cerevisiae, Tolypocladium*, 6mA: *A. thaliana, C. elegans, C. equisetifolia, D. melanogaster, F. vesca, H. sapiens*, R. chinensis, S. cerevisiae, T. thermophile, Tolypocladium, XocBLS256)	BERT	FGM	5hmC_*H. sapiens*: Acc = 0.9501, Sn = 0.9838, Sp = 0.9164, AUROC = 0.9501, MCC = 0.9022 5hmC_*M. musculus*: Acc = 0.9679, Sn = 0.969, Sp = 0.9668, AUROC = 0.9679, MCC = 0.9358 4mC_C. equisetifolia: Acc = 0.8579, Sn = 0.8743, Sp = 0.8415, AUROC = 0.8579, MCC = 0.7162 4mC_F. vesca: Acc = 0.8524, Sn = 0.8535, Sp = 0.8512, AUROC = 0.8524, MCC = 0.7047 4mC_S. cerevisiae: Acc = 0.723, Sn = 0.6876, Sp = 0.7583, AUROC = 0.723, MCC = 0.447 4mC_Tolypocladium: Acc = 0.7434, Sn = 0.7385, Sp = 0.7483, AUROC = 0.7434, MCC = 0.4868 6mA_A. thaliana: Acc = 0.8603, Sn = 0.8264, Sp = 0.8942, AUROC = 0.8603, MCC = 0.7223 6mA_C. elegans: Acc = 0.9138, Sn = 0.9256, Sp = 0.902, AUROC = 0.9138, MCC = 0.8279 6mA_C. equisetifolia: Acc = 0.7399, Sn = 0.6713, Sp = 0.8084, AUROC = 0.7399, MCC = 0.4843 6mA_*D. melanogaster*: Acc = 0.9228, Sn = 0.9301, Sp = 0.9155, AUROC = 0.9228, MCC = 0.8457 6mA_F. vesca: Acc = 0.9413, Sn = 0.9452, Sp = 0.9375, AUROC = 0.9413, MCC = 0.8827 6mA_R. chinensis: Acc = 0.8629, Sn = 0.8328, Sp = 0.893, AUROC = 0.8629, MCC = 0.7271 6mA_S. cerevisiae: Acc = 0.8278, Sn = 0.7966, Sp = 0.859, AUROC = 0.8278, MCC = 0.6569 6mA_T. thermophile: Acc = 0.8804, Sn = 0.9442, Sp = 0.8167, AUROC = 0.8804, MCC = 0.7671 6mA_Tolypocladium: Acc = 0.7771, Sn = 0.7649, Sp = 0.7892, AUROC = 0.7771, MCC = 0.5543 6mA_Xoc BLS256: Acc = 0.8817, Sn = 0.8808, Sp = 0.8827, AUROC = 0.8817, MCC = 0.7634
		(250)	DNAm dataset (Brain, Blood, Buccal, Saliva)	Transformer	_	SRCC: (Brain: Mean = 0.82, SD = 0.004) (Blood: Mean = 0.78, SD = 0.005) (Buccal: Mean = 0.79, SD = 0.007) (Saliva: Mean = 0.79, SD = 0.010) Mean squared error: (Brain: Mean = 0.030, SD = 0.0026) (Blood: Mean = 0.043, SD = 0.0023) (Buccal: Mean = 0.049, SD = 0.0080) (Saliva: Mean = 0.040, SD = 0.055)
		(146)	1. Maize 5mC dataset 2. Nipponbare 5mC dataset 3. 6mA Chen dataset 4. 6mA Lv dataset	ULMFiT	_	1. Maize 5mC Acc = 0.9524, AUNP = 0.97, AUNU = 0.95, Macro precision = 0.9378, Min precision = 0.9524, Macro Sn = 0.9188, Micro Sn = 0.9524, Macro F1-score = 0.9269, Min F1-score = 0.9524 2. Nipponbare 5mC Acc = 0.8106, AUNP = 0.88, AUNU = 0.88, Macro precision = 80.74, Min precision = 81.93, Macro Sn = 81.93, Micro Sn = 81.93, Macro F1-score = 80.32, Min F1-score = 81.93 3. 6mA Chen Sn = 0.9303, Sp = 0.9225, Acc = 0.9265, MCC = 0.85, AUROC = 0.93 4. 6mA Lv Sn = 0.9605, Sp = 0.9248, Acc = 0.9426, MCC = 0.89, AUROC = 0.94
		(154)	Lv et al. datasets (5hmc: *M. musculus, H. sapiens*, 4mc: *C. equisetifolia, F. vesca, S. cerevisiae, Tolypocladium*, 6mA: *A. thaliana, C. elegans, C. equisetifolia, D. melanogaster, F. vesca, H. sapiens, R. chinensis, S. cerevisiae, T. thermophile, Tolypocladium*, XocBLS256)	BERT	_	5hmC_*H. sapiens*: Acc = 94.92, Sn = 98.63, Sp = 91.21, MCC = 90.09, AUROC = 95.53, F1-score = 95.1 5hmC_*M. musculus*: Acc = 96.85, Sn = 97.06, Sp = 96.63, MCC = 93.69, AUROC = 97.57, F1-score = 96.85 4mC_*C. equisetifolia*: Acc = 82.51, Sn = 79.23, Sp = 85.79, MCC = 65.17, AUROC = 85.55, F1-score = 81.92 4mC_F. vesca: Acc = 84.2, Sn = 85.2, Sp = 83.21, MCC = 68.42, AUROC = 90.7, F1-score = 84.36 4mC_*S. cerevisiae*: Acc = 70.27, Sn = 66.94, Sp = 73.61, MCC = 40.64, AUROC = 75.37, F1-score = 69.25 4mC_*Tolypocladium*: Acc = 73.83, Sn = 72.16, Sp = 75.49, MCC = 47.68, AUROC = 80.57, F1-score = 73.39 6mA_A. thaliana: Acc = 85.38, Sn = 82.33, Sp = 88.42, MCC = 70.88, AUROC = 91.84, F1-score = 84.92 6mA_*C. elegans*: Acc = 89.03, Sn = 88.17, Sp = 89.9, MCC = 78.08, AUROC = 94.33, F1-score = 88.94 6mA_*C. equisetifolia*: Acc = 73.28, Sn = 68.91, Sp = 77.65, MCC = 46.73, AUROC = 79.02, F1-score = 72.06 6mA_*D. melanogaster*: Acc = 91.22, Sn = 90.38, Sp = 92.05, MCC = 82.44, AUROC = 95.44, F1-score = 91.14 6mA_*F. vesca*: Acc = 92.68, Sn = 92.33, Sp = 93.04, MCC = 82.44, AUROC = 95.44, F1-score = 92.66 6mA_*H. sapiens*: Acc = 89.8, Sn = 89.4, Sp = 90.2, MCC = 79.6, AUROC = 95.1, F1-score = 89.76 6mA_*R. chinensis*: Acc = 82.61, Sn = 80.94, Sp = 84.28, MCC = 65.25, AUROC = 87.89, F1-score = 82.31 6mA_*S. cerevisiae*: Acc = 80.11, Sn = 72. 37, Sp = 87.85, MCC = 60.96, AUROC = 87.09, F1-score = 78.44 6mA_*T. thermophile*: Acc = 87.4, Sn = 93.34, Sp = 81.54, MCC = 75.4, AUROC = 93.1, F1-score = 88.14 6mA_*Tolypocladium*: Acc = 77.38, Sn = 71.76, Sp = 83.01, MCC = 55.12, AUROC = 83.61, F1-score = 76.04 6mA_Xoc BLS256: Acc = 86.94, Sn = 88.9, Sp = 84.92, MCC = 73.94, AUROC = 92.61, F1-score = 87.2
**Goal: environmental and microbial genomics tasks**						
Multi-class classification	Nitrogen cycle prediction	(27)	NCycDB	BERT	_	Macro F1-score = 99.5, Weighted F1-score = 99.2
**Goal: gene network analysis**						
Multi-class classification	Gene taxonomy classification	(121)	Verma et al. dataset	FastText	MLP	Macro F1-score = 0.92 ± 0.0054
		**(** **123** **)**	**Mock et al. dataset (Superkingdom, Phylum)**	**BERT**	_	**Superkingdom: Acc = 94.78 Phylum: Acc = 85.55**
		(122)	ActinoMock dataset (LSH, Decimal, FNV)	LSH+FastText	MLP	LSH: Acc = 1.00, Precision = 1.00, Recall = 1.00, F1-score = 0.99 Decimal: Acc = 0.93, Precision = 0.94, Recall = 0.93, F1-score = 0.91 FNV: Acc = 0.937, Precision = 0.94, Recall = 0.94, F1-score = 0.92
Binary classification	Gene network reconstruction	(127)	DREAM4 10, DREAM4 100, E. coli cold	_	LightGBM	DREAM4 10: AUROC = 0.956, AUPRC = 0.891 DREAM4 100: AUROC = 0.909, AUPRC = 0.445 E. coli cold: AUROC = 0.602, AUPRC = 0.030
		(126)	Pio et al. dataset	Metabolic feature encoding	Clustering	_
		(128)	Ceci et al. datasets	Node2Vec	PCT	_

Bold values highlight top performers on unique datasets related to distinct tasks.

Second most commonly used representation learning approach in this goal is Word2vec which is explored with two unique classifiers for two different tasks. Specifically, Word2vec with CNN classifier is used for one task, namely, 4mC-cytosine modification prediction, and with BiLSTM for one task, namely, 6mA-methyl adenine modification prediction. From most common approaches, BERT manages to achieve best performance with a self-classifier as compared to Word2vec-based predictive pipeline. Apart from BERT and Word2vec, Transformer with a self-classifier is used for three tasks, namely, 5mC-methycyctosine modification prediction, 6mA-methyl adenine modification prediction, and DNA methylation modification prediction. Transformer is also used with CNN classifier on one of the common task, namely, 6mA-methyl adenine modification prediction. Furthermore, potential of transformer-XL is explored with CNN classifier for one task, ULMFiT and hybrid encoding scheme (BERT+ALBERT+XLNet+ELECTRA) with self-classifier for one task, FastText with SVM classifier for one task and FastText with XGBoost classifier for one task. Overall, among all approaches, PseKNC encoding approach with structural sparse regularized random vector functional link network classifier manages to achieve best predictive performance on 4mC-methylcytosine modification prediction. Among all four tasks, DNA methylation modification prediction and 5mC-methyl cytosine modification prediction have some room for improvement. Building on the performance trends of predictive pipelines developed for different tasks of this goal, potential of BERT or PseKNC representation learning approach with structural sparse regularized random vector functional link network classifier can be explored to enhance the performance of under-performing tasks.

For environmental and microbial genomics goal, only potential of BERT representation learning is explored with a self-classifier. However, the potential of neural word embeddings and domain specific encoders based predictive pipelines remains unexplored.

For gene network analysis goal, across two different tasks, four unique representation learning approaches, namely, FastText, Node2vec, BERT, and metabolic encoding, along with four unique classifiers, namely, MLP, LightGBM, Clustering algorithm, and PCT, are used by six predictors. FastText representation learning is most commonly used among all approaches. Specifically, FastText along with MLP classifier is used for gene taxonomy classification task. Second most common representation learning approach is BERT that is used with a self-classifier for same gene taxonomy classification task. Apart from this, Node2Vec representation is explored with PCT classifier and metabolic feature encoding is explored with clustering algorithm for gene network reconstruction task. Among all approaches, FastText and MLP classifier-based predictive pipeline manages to achieve best performance for gene taxonomy classification task. Among all tasks of this goal, gene taxonomy classification offers some room for improvement. Building on promising performance achieved by contemporary language models for different sequence analysis tasks, hierarchical graph transformer and sophisticated machine or deep learning-based ensemble classifier can further raise the predictive performance on gene taxonomy classification task. Furthermore, advanced graph-based representation learning methods, GraRep, HOPE, and LINE with deep classifiers can also potentially raise the predictive performance on gene taxonomy classification task.

In addition, Table 11 provides an overview of six DNA sequence analysis tasks classified under the goal of DNA functional analysis. For this goal, four unique representation learning methods, namely, Word2Vec, Transformer, BERT, and FastText, are used in conjunction with three different predictors, namely, LogR, SVM, and cosine similarity. Among all four representation learning methods, Transformer is most commonly used followed by Word2vec and BERT. Transformer is used in three different tasks with self-classifier, Word2vec, and BERT are used with LogR and self-classifier in two different tasks. In addition, potential of FastText is explored with SVM for 1 task. Among all representation learning methods, Transformer with self-classifier manages to achieve top performance for tumor type prediction. Among all six tasks, disease risk estimation task offers a room for improvement as its respective BERT and self-classifier-based predictive pipeline accuracy falls approximately 56%. Hybrid approaches combining the powers of Transformer, BERT, and Word2vec with sophisticated machine learning classifier such as deep forest or deep learning classifiers such as CNN and CNN+BiGRU can potentially enhance the performance on under-performing tasks. Furthermore, except for two tasks, namely, species classification and functional prioritization, of non-coding variants, all other four tasks are evaluated on a single benchmark dataset. Considering deep learning models require huge amount of data to achieve promising performance, development, and utilization of more datasets in model building and validation can also prove fruitful for enhancing the predictive performance.

**Table 11:** DNA functional analysis task predictive pipeline performance.

**Task type**	**Task name**	**References**	**Dataset**	**Representation learning**	**Classifier**	**Performance evaluation**
Binary classification	Conserved non-coding elements classification	(163)	Polychronopoulos et al. dataset (D1, D2, D3)	Word2Vec	LogR	D1: F1-score = 83.0, D2: F1-score = 85.5, D3: F1-score = 77.4
Multi-class classification	Functional prioritization of non-coding variant	(34)	Yang et al. dataset (LOGO-919, LOGO-2002, LOGO-3357)	Transformer	_	_
Binary classification	Exon and intron region classification	**(** **164** **)**	**Akalin et al. dataset**	**BERT**	_	**Precision = 100, Sn = 75, Sp = 100, Acc = 88.88, F1-score = 85.71**
Binary classification	Recombination spots identification	**(** **165** **)**	**Liu et al. dataset**	**FastText**	**SVM**	**Sn = 90, Sp = 94.76, Acc = 92.6, MCC = 0.851**
Multi-class classification	Species classification	(44)	1. Mouse enhancers dataset 2. Coding vs. intergenomic dataset 3. Human vs. worm dataset 4. Human enhancers cohn dataset 5. Human enhancers ensembl dataset 6. Human regulatory dataset 7. Human nontata promoter dataset 8. Human OCR ensembl dataset	Transformer	_	Mouse enhancers: Acc = 85.1 Coding vs. Intergenomic: Acc = 91.3 human vs. worm: Acc = 96.6 human enhancers cohn: Acc = 74.2 human enhancers ensembl: Acc = 89.2 human regulatory: Acc = 93.8 human nontata promoter: Acc = 96.6 human OCR ensembl: Acc = 80.9
Interaction	Prediction of context-specific functional impact of genetic variants	**(** **36** **)**	**eQTLs dataset**	**Transformer**	_	**AUPRC = 0.922**

Bold values highlight top performers on unique datasets related to distinct tasks.

Finally, Table 12 summarizes predictive models developed for seven unique DNA sequence analysis tasks categorized under the goal of disease analysis. For this goal, seven unique representation learning methods, namely, Node2Vec, Graph2Vec, BERT, Graph Embedding, SDNE, Word2Vec, and Transformer, and five predictors, namely, MLP, RF, cosine similarity, clustering, and BERT self-classifier, are used in different tasks. Among all representation learning approaches, BERT and Word2vec are most commonly used. BERT is used with self-classifier on two different tasks, and word2vec is used with cosine similarity for a multi-class classification task, namely, mutation susceptibility analysis and with clustering algorithm for an only clustering task, namely, phylogenetic analysis. Apart from BERT and Word2vec, other representation learning methods Node2vec+Graph2vec, Graph Embedding, SDNE+Word2vec, and Transformer are used on one classification task each with MLP and self-classifiers. Overall, among all approaches, Transformer with self-classifier-based predictive pipelines manages to achieve best performance on tumor type prediction task. Among all seven tasks, disease risk estimation task offers a lot of room for improvement as its respective BERT with self-classifier-based predictive pipeline performance falls approximately 56%. Taking the transformer performance trends into account, latest sophisticated language models such as hierarchical graph transformer, ELECTRA, and GPT-4 along with ensemble machine or deep learning predictors can achieve significance performance rise in under-performing classification and clustering tasks.

**Table 12:** Disease analysis task predictive pipeline performance.

**Task type**	**Task name**	**References**	**Dataset**	**Representation learning**	**Classifier**	**Performance evaluation**
Binary classification	Pathogen signatures identification	(171)	DS500 dataset, DS5000 dataset	Node2Vec+Graph2Vec	MLP	1. Acc = 73.49, 2. Acc = 89.7
	Disease risks estimation	**(** **90** **)**	**HSCR-RET, HSCR-RET-Long**	**BERT**	_	**HSCR-RET: Precision = 0.770, Recall = 0.519, Acc = 0.562; HSCR-RET-Long: Precision = 0.768, Recall = 0.513, Acc = 0.541**
Interaction	Phage-host interactions prediction	**(** **175** **)**	**Qiu et al. dataset**	_	**RF**	**Acc = 0.801, Recall = 0.801, Sp = 0.801, Precision = 0.803, F1-score = 0.801, AUROC = 0.801**
		(176)	Wang et al. dataset	Graph Embedding	MLP	AUROC = 0.88317
		(177)	ESKAPE dataset	SDNE+Word2Vec	**MLP**	Acc = 86.65 ± 1.55, Sn = 88.40 ± 1.81, Sp = 84.91 ± 1.96, Precision = 85.43 ± 1.74, F1-score = 86.88 ± 1.53, AUROC = 0.9208 ± 0.0119
Multi-class classification	Mutation susceptibility analysis	**(** **173** **)**	**Yilmaz et al. dataset (Human, Mouse)**	**Word2Vec**	**Cosine similarity**	**Human data: Acc = 0.7974, mouse data: Acc = 0.8322**
	Tumor type prediction	**(** **180** **)**	**TCGA pan-cancer dataset**	**Transformer**	_	**Acc = 98.4, Precision = 98.50, Recall = 98.4, F1-score = 98.37**
	Pathogenicity potential assessment	(27)	E-K12, CARD-A, CARD-D, CARD-R, VFDB, ENZYME, PATRIC, NCycDB	BERT	_	E-K12: Macro F1-score = 61.8, Weighted F1-score = 65.4; CARD-A AMR: Macro F1-score = 78.6, Weighted F1-score = 90.1; CARD-D: Macro F1-score = 57.4, Weighted F1-score = 85.2; CARD-R: Macro F1-score = 69.4, Weighted F1-score = 91.4; VFDB: Macro F1-score = 75.7, Weighted F1-score = 90.2; ENZYME: Macro F1-score = 99.1, Weighted F1-score = 98.8; PATRIC: Macro F1-score = 99.3, Weighted F1-score = 99.0; NCycDB: Macro F1-score = 99.5, Weighted F1-score = 99.2
Clustering	Phylogenetic analysis	(21)	Ren et al. dataset	Word2Vec	Clustering	Acc = 0.84

Bold values highlight top performers on unique datasets related to distinct tasks.

In a nutshell, a comprehensive analysis of state-of-the-art predictive pipelines developed using word embeddings, language models, and nucleotide compositional and positional information-based encoders reveals interesting trends. From 44 DNA sequence analysis tasks classified under the hood of 8 major biological goals, 24 tasks belong to binary classification, 4 belong to interaction prediction, 11 belong to multi-class classification, only 3 belong to multi-label classification, 1 belong to regression, and 1 belong to clustering. Overall, 25 unique representation learning methods and 28 predictors are explored for developing robust predictive pipelines for 44 DNA sequence analysis tasks classified under the hood of 8 major biological goals. Across all eight goals, language model-based representation learning approaches and deep learning classifiers are achieving better performance across majority of the tasks. Researchers can explore the performance potential of latest transformer-based language models such as Hierarchical graph transformer, GPT-4, and hybrid representation learning methods along with sophisticated ensemble machine learning or deep learning predictors for different classification, regression, and clustering tasks.

## 11 Publisher and journal-wise distribution of research articles

This section provides an overview of 44 distinct DNA sequence analysis task-related articles distribution across conferences, journals, and publishers. Before paper submission, identification of relevant journals for a study publication in the interdisciplinary field of AI applications in DNA sequence analysis is an important task. There are three types of journals in AI and DNA sequence analysis fields: (1) Journals focusing on core AI algorithms, (2) Journals dedicated to core biological findings, (3) Hybrid journals that publish research integrating both AI algorithms and biological data. Researchers often face desk rejections when submitting to core AI or biology journals. Instead, they should target hybrid journals. While many tools exist to find suitable journals, this comprehensive guide provides detailed information to help researchers to identify journals where applications using word embeddings and large language models for DNA sequence analysis are published.

**Figure 7:**
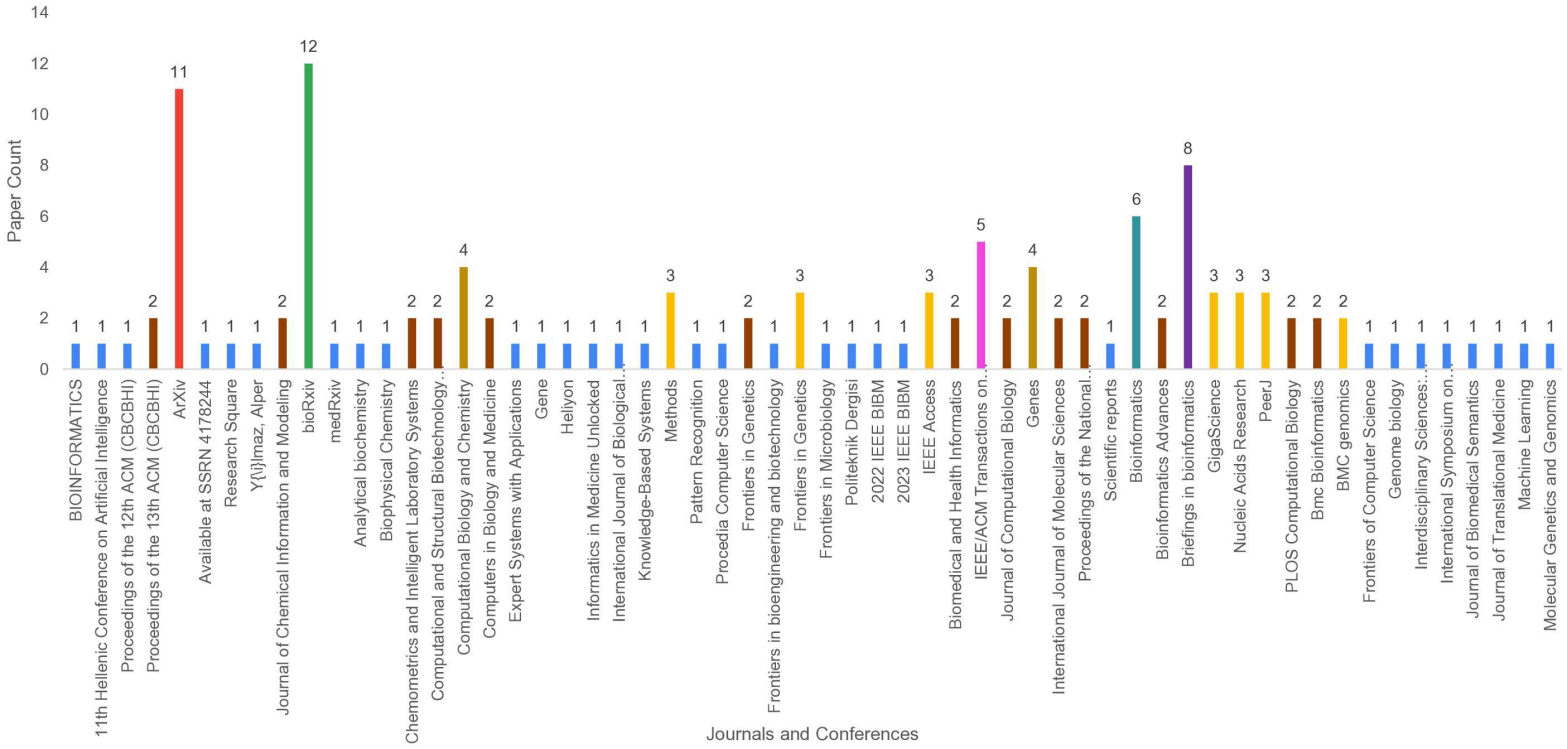
Publication distribution of DNA sequence analysis literature across diverse journals and conferences from 2018 to 2024.

Figure 7 graphically depicts distribution of 127 studies across 53 journals, 1 transactions, 3 conferences, and 2 pre-print repositories. Among all journals, more studies are published in Briefings in Bioinformatics followed by Bioinformatics, Computational Biology, and Chemistry, and International Journal of Molecular Sciences. Similarly, among all conferences, more studies are published in the International Conference on Bioinformatics and Biomedicine (BIBM) followed by the 11^*th*^ Hellenic Conference on Artificial Intelligence, Proceedings of the 12th and 13^*th*^ ACM International Conference on Bioinformatics, Computational Biology, and Health Informatics. Moreover, 5 studies are published in ACM transaction of computational biology. In the light of rapid development in research findings, researchers have also published 24 studies in bioRxiv, ArXiV, and MedXiv platforms. However, researchers generally prefer journal publications for their sustained impact.

**Figure 8:**
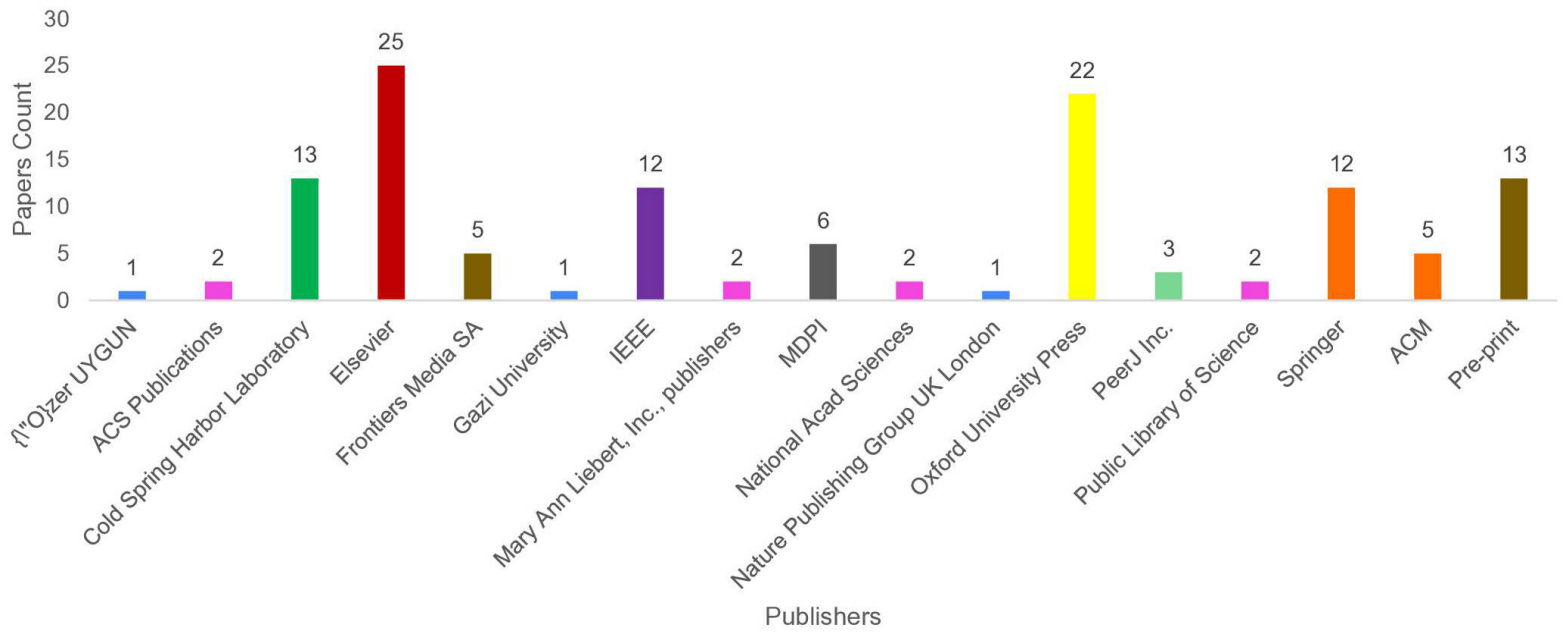
Distribution of publishers involved in the publication of DNA sequence analysis literature from 2018 to 2024.

Furthermore, Figure 8 illustrate that 127 DNA sequence analysis studies are published by 17 different publishers, namely, Springer,^9^ Elsevier (see text footnote ^3^), Oxford University Press,^10^ Cold Spring Harbor Laboratory,^11^ IEEE,^12^ Ozer UYGUN,^13^ ACS Publication,^14^ Frontier Media SA,^15^ Gazi University,^16^ Marry Ann Liebert,^17^ MDPI,^18^ National Acad Sciences,^19^ Nature Publishing Group UK London,^20^ PeerJ Inc.,^21^ Public Library of science,^22^ ACM (see text footnote ^2^), and pre-prints.^23^ Notably, approximately 60 out of 127 DNA sequence analysis studies are published by Oxford University Press, Elsevier, and Cold Spring Harbor Laboratory. In addition, IEEE, Springer, and MDPI have contributed 30 relevant papers. Furthermore, 32 DNA sequence analysis research articles are published by ACS Publications, Frontiers Media SA, Mary Ann Liebert, Inc., National Acad Sciences, Nature Publishing Group UK London, Public Library of Science, PeerJ Inc, and others. Collectively, 96 are journal publications, 6 are conference papers, 1 is transaction articles, and 24 are pre-prints out of 127 DNA sequence analysis studies published by 21 different publishers. This comprehensive analysis across various journals, conferences, transactions, and pre-print repositories highlights diverse and extensive research landscape in DNA sequence analysis.

## 12 Discussion

We acknowledge that “DNA sequence analysis” encompasses a much broader range of bioinformatics applications than covered in this review. Our focus is specifically on AI-based approaches that analyze raw DNA sequence data to predict biological functions and features. Other important areas of bioinformatics such as genome assembly, comprehensive variant analysis, phylogenomics, and many aspects of population genetics utilize different computational approaches and would benefit from separate dedicated reviews. A comprehensive review of existing literature on AI-driven DNA sequence analysis tasks reveals a significant inconsistency in the evaluation of predictive pipelines across similar datasets. Researchers have developed numerous datasets tailored to specific tasks, and most of the researchers have evaluated their proposed predictors solely on their own datasets.

The creation of new datasets is essential because public databases are frequently updated with new sequence information. These new datasets can incorporate the most recent sequence data alongside existing information. Moreover, existing datasets tend to be smaller, while deep learning models perform better with larger datasets. To address performance comparison inconsistency, there is an urgent need to standardize dataset utilization. One potential solution is to benchmark existing predictors on newly developed datasets and compare the performance of proposed predictors against these benchmarks. This approach would provide a more objective evaluation of proposed predictor performance. However, in this context, a significant challenge is the limited availability of source codes for existing predictors. Many studies make their source code private, hindering the reproducibility of results and direct comparison with other methods. To streamline the integration of innovative methods and ensure methodological advancement, it is crucial to analyze task-specific datasets and create standardized datasets with detailed descriptions. By benchmarking the performance of existing predictors on these standardized datasets, researchers can establish a common ground for comparison and facilitate more accurate evaluations of models. This approach would enhance transparency and reproducibility in DNA sequence analysis studies.

The development of AI-driven predictive pipelines for DNA sequence analysis relies heavily on effective sequence representation learning methods and appropriate machine or deep learning models. Machine and deep learning models inherently depend on statistical vectors and cannot process raw DNA sequences directly. Therefore, the role of representation learning methods in these pipelines is crucial. These methods are responsible for transforming raw DNA sequences into statistical vectors by capturing and encoding the most informative nucleotide patterns.

In the current landscape of AI-driven DNA sequence analysis, researchers have employed a variety of representation learning methods, including 12 distinct word embedding techniques and 8 language models. However, when it comes to other genetic molecules such as RNA and proteins, researchers have explored an additional set of 17 word embedding methods and 13 language models that have not yet been applied to DNA sequence analysis. These unexplored word embedding methods include DANE (285), ELMo (286–288), GATNE (289), GEMSEC (290), MetaGraph2Vec (291), HAKE (292), HIN2Vec (293), HOPE (294, 295), LINE (296–298), Mashup (299, 300), Random Watcher-Walker (RW2) (301), RotatE (292, 302, 303), RWR (304), Struc2Vec (305, 306), SVD (307, 308), Topo2Vec (309), and TransE (310), while the unexplored language models include AlphaFold (311–315), AlphaFold2 (316, 317), BigBird (318), ESM-1 (315, 319, 320), ESM-2 (109, 286, 316, 320), Graph Transformer Network (321), Heterogeneous Graph Transformer (322), IgFold (323), LongFormer (318), RoBERTa (324, 325), T5 (320, 326–328), and Vision Transformer (288). Integrating these advanced word embedding techniques and large language models into AI-driven DNA sequence analysis pipelines could potentially enhance their performance and robustness.

Within 127 AI-driven DNA sequence analysis predictive pipelines, researchers have utilized 18 machine and deep learning algorithms at the predictor level. In some cases, they have developed meta-predictors by combining multiple machine learning and deep learning algorithms to enhance predictive performance. However, similar to the representation learning stage, there are 24 distinct methods at the predictor level that have not yet been explored, representing untapped potential for improving the accuracy and robustness of these AI-driven pipelines.

Our categorization of 44 distinct tasks into 8 biological goals provides a structured framework that serves as a valuable starting taxonomy for both computer scientists and life scientists. This organization is informed by both computational and biological literature and creates a common reference point that bridges these disciplines while facilitating interdisciplinary communication. We recognize the inherent complexity of biological systems and the interconnected nature of many of these tasks. For example, enhancer identification categorized under gene expression regulation shares biological connections with chromatin accessibility prediction categorized under genome structure and stability. Nevertheless, this framework offers a practical organizing principle that will naturally evolve and be refined over time. The taxonomy presented here lays groundwork that future collaborative efforts between AI researchers and domain specialists in genomics can build upon. We anticipate gradual development into a more nuanced framework that maintains practical utility while better reflecting biological realities. Similar to many scientific classification systems, we expect this taxonomy to mature through iterative refinement as the field advances.

## 13 Conclusion

This review serves as a comprehensive resource for researchers working at the intersection of AI and DNA sequence analysis. It provides a structured foundation for future innovations in the rapidly evolving field of computational genomics. It bridges the critical gap between molecular biology and artificial intelligence by systematically analyzing 44 different DNA sequence analysis tasks, their associated databases, datasets, and AI methodologies. It identifies 36 biological databases and 140 benchmark datasets that provide a robust foundation for developing and evaluating AI predictors. Furthermore, our examination of existing predictive pipelines demonstrates the successful application of 39 word embeddings and 67 language models across various DNA sequence analysis tasks. Our analysis reveals that while significant progress has been made in developing AI-driven predictive pipelines for DNA sequence analysis, several challenges and opportunities remain unexplored. Several promising directions emerge for the advancement of this field. First, the integration of 17 unexplored word embedding methods and 13 language models (currently utilized only for RNA and protein analysis) could significantly enhance DNA sequence analysis capabilities. Second, the development of standardized benchmark datasets and evaluation protocols would facilitate fair comparisons between different predictive models and accelerate progress in the field. Third, the adoption of 24 untapped machine learning and deep learning algorithms at the predictor level presents an opportunity to improve prediction accuracy and robustness.

Future research should focus on developing multi-task learning frameworks that can simultaneously handle multiple DNA sequence analysis tasks, thereby improving computational efficiency and leveraging shared biological features. Furthermore, ensuring public accessibility of source codes and detailed documentation of predictive pipelines would foster reproducibility and collaborative advancement in the field. The establishment of standardized performance metrics and evaluation protocols across different DNA sequence analysis tasks would enable more meaningful comparisons between various approaches and guide future developments. As DNA sequence data continue to grow exponentially, the integration of more sophisticated AI architectures, particularly those capable of handling large-scale genomic data efficiently, will become increasingly important. Our categorization of 44 tasks reflects common AI applications in DNA sequence analysis literature and provides a starting point for interdisciplinary discourse. We recognize that deeper collaboration between AI researchers and life scientists would further strengthen the biological relevance of this framework. A greater amount of input from geneticists and bioinformaticians would be essential to develop a more comprehensive and biologically relevant tasks taxonomy.

## Footnotes

^1^ https://scholar.google.com/

^2^ https://dl.acm.org/

^3^ https://www.elsevier.com/

^4^ https://ieeexplore.ieee.org/

^5^ https://www.wiley.com/en-us

^6^ https://www.springer.com/gp

^7^ https://www.sciencedirect.com/

^8^ https://research.google/blog/more-efficient-nlp-model-pre-training-with-electra/

^9^ https://www.springer.com/in

^10^ https://global.oup.com/academic/

^11^ https://www.cshlpress.com/

^12^ https://www.ieee.org/

^13^ https://dergipark.org.tr/en/pub/@ozeruygun

^14^ https://pubs.acs.org/

^15^ https://www.frontiersin.org/

^16^ https://gazi.edu.tr/

^17^ https://www.liebertpub.com/

^18^ https://www.mdpi.com/

^19^ https://www.nasonline.org/

^20^ https://www.nature.com/

^21^ https://peerj.com/

^22^ https://plos.org/

^23^ https://arxiv.org/
